# Refining the chronology of North America’s copper using traditions: A macroscalar approach via Bayesian modeling

**DOI:** 10.1371/journal.pone.0266908

**Published:** 2022-04-26

**Authors:** Michelle R. Bebber, Briggs Buchanan, Jacob Holland-Lulewicz

**Affiliations:** 1 Department of Anthropology, Kent State University, Kent, Ohio, United States of America; 2 Department of Anthropology, University of Tulsa, Tulsa, Oklahoma, United States of America; 3 Department of Anthropology, Washington University in St. Louis, St. Louis, Missouri, United States of America; New York State Museum, UNITED STATES

## Abstract

North America’s ancient copper use, predicted to originate as early as 9000 cal BP, represents the earliest use of native copper for utilitarian tool production in the world. Although recent work has focused on establishing the first use of copper in the western Great Lakes region, little attention has been paid to determining the age ranges of subsequent copper using groups or to the identification of broader trends in copper use during the Archaic Period (10,000–3000 RCYBP). Here we address this issue by applying Bayesian modeling to a comprehensive suite of 76 radiocarbon dates directly associated with copper use. Our results identified two distinct peaks in copper usage, ca. 5500 cal BP and ca. 3300 cal BP. Age ranges for the three Archaic Period traditions and practices associated with copper use of the western Great Lakes are revised using modern calibration curves. Bayesian revisions of age ranges from sites where copper tools and/or production debris have been found provide insight into the historical relationships between, and cultural interactions among, these early copper using groups. This study provides an updated, refined chronology based on the most recent calibration curve (IntCal20) for the varied cultural contexts of copper use across the western Great Lakes.

## Introduction

North America’s ancient copper use, predicted to originate as early as 9000 cal BP [1], represents the earliest use of native copper for utilitarian tool production in the world. However, although recent work has focused on establishing the “first” use of copper in the western Great Lakes region, little attention has been paid to addressing the temporalities of subsequent copper using traditions and practices. Here we address this issue by applying Bayesian modeling to a comprehensive suite of 76 radiocarbon dates, directly associated with copper use, to identify temporal patterns over the Archaic Period (10,000–3000 RCYBP). Age ranges for the three Archaic Period traditions and practices associated with copper use of the Great Lakes—traditionally defined by the culture-historical monikers Old Copper, Burnt Rollways, and Red Ocher—are revised using modern calibration curves. We recognize the heuristic nature of these essentialized groupings, though we also recognize that each of these are associated with particular traditions and practices that give varying social, cultural, and historical context to the long-term use of copper in the region. As such, through the refinement of the age ranges associated with these traditions, we build towards a more chronologically sound and temporally complex landscape of copper using societies, traditions, and histories that defined the western Great Lakes region between ca. 10,000–3000 RCYBP. In particular, we attempt to resolve issues related to the interactions of diverse copper-using societies, to the varied historical trajectories of copper-using traditions, and to the potential movements, evolutions, and transformations of particular practices across a complex sociocultural matrix of groups living around the western Great Lakes. To further this goal, we include 12 dates from Archaic Period copper sites in the Ottawa River Valley, Canada, which are often excluded from broader discussions of early copper use in the Americas. In this regard, this research program is situated within a broad, macroscalar perspective to better temporally situate the peoples involved in the production, use, and exchange of copper materials at geographic scales that are not often considered.

### The Archaic Period

The onset of the Archaic Period in North America varies by region, beginning earlier in the southern areas of the Eastern Woodlands (ca. 10,000 BP [2]) compared to regions surrounding the Upper Great Lakes, where certain Paleoindian technologies continue until at least 8000 BP and possibly as late as 7545 BP [3, 4]. Indeed, archaeological evidence suggests overlap in material culture, namely stone projectiles, associated with both Late Paleoindian and Early Archaic groups [5–12]. Whether these data signal a co-mingling between discrete peoples, or a historical relationship between Late Paleoindian and Early Archaic groups is a topic not fully understood. This problem generates uncertainty regarding the onset of the traditions associated with the Archaic Period in the Upper Great Lakes.

Given the long duration of the Archaic Period (10,000–3000 BP), it is commonly discussed in terms of three subphases, termed simply Early (10,000–8000 BP), Middle (8000–5000 BP), and Late (5000–3000 BP) Archaic. However, as with the onset and termination of the Archaic Period in general, the precise timing of these subphases varies by region, with diagnostic characteristics occurring earlier in the more southerly regions and expanding northward in a time-transgressive fashion. Broadly, the onset of the Early Archaic is signaled by the presence of stemmed and notched bifacial flint tools. Surprisingly little is known about human lifeways during this era due to a paucity of archaeological sites, but it is generally accepted that groups were small, dispersed, highly mobile, and occasionally used rockshelters [13–16]. Aquatic and wetland resources were heavily exploited in the early Holocene as the climate of the Upper Great Lakes was wetter and cooler than in the Middle Archaic when the climate became warmer and began to stabilize [17, 18]. During the Middle Archaic archeological materials increase, and the first mortuary focused sites appear in North America. Based on assemblage data, researchers have inferred that Archaic groups were broad-spectrum foragers who would often seasonally aggregate to share resources and exchange items. In the western Great Lakes, fishing and aquatic resources were important, along with a wide variety of woodland game, fowl, nuts, and wild plants [13, 19].

Of central interest here are the temporalities of the various traditions, practices, and histories of groups who inhabited this region and were the direct users of native copper as tools or ornaments. Despite sustained interest in establishing the timing for copper usage via radiocarbon dating throughout the second half of the twentieth century, this topic has received little attention in the past decade. Early research on North American Archaic Period copper use focused on broad topics of cultural origins and delineation, primarily using excavation data from cemeteries [20–23] and analysis of artifacts collected from the surface or other non-mortuary archaeological contexts [24–27]. Later studies focused on the role of copper in Archaic Period societies [28], the nature of tool use [29, 30], tool production techniques [31–33], and copper sourcing [34–38]. Recent research has focused on the nature of exchange-based relationships [39, 40], population dynamics and changing social structure [41], as well as experimental research on copper tool functional efficiency and the interrelated factors affecting technological change [42–45].

Indeed, although North American copper usage has been a topic of interest for centuries, the chronological relationships and temporalities for the various copper using groups are still relatively uncertain, mainly due to small sample sizes and the vagaries of dating [46]. Here we use Bayesian modeling to establish updated chronologies for three distinct cultural phenomenon differentially inscribed across the western Great Lakes landscape, 1) Middle Archaic mortuary sites located far from copper sources, referred to herein as “Old Copper” traditions, 2) Late Archaic mortuary sites located nearer to copper sources referred to herein as “Red Ocher” traditions, and 3) Late Archaic production sites located near copper sources, referred to herein as “Burnt Rollways” traditions. In contrast to extant work that treats these traditions as separate “cultures” or “peoples,” we instead conceptualize these as merely three different kinds of archaeological sites that represent different and varied cultural expressions and practices. These differences, described in detail below, are primarily defined by distinct mortuary practices, unique uses of copper, and the presence or absence of associated habitations and tool-working areas. The goal is to model age ranges for each of these site types, which have only to this point used the “eye ball” approach, masking important temporalities and chronological relationships between distinct social, cultural, and economic practices [47]. Once revised age ranges have been established, we can move beyond simple associations of the archaeological record with distinct “cultures” to better determine the historical relationships, as well as clarify when/if there were opportunities for interrelationships to have existed between the peoples who frequented these functionally distinct sites. Here we provide a Bayesian revision for the age ranges for the three main copper using cultural phenomena of the Archaic Period in the Great Lakes region in order to better clarify their temporal position and to identify broader patterns in native copper tool occurrence.

### Copper-using groups of the Great Lakes

#### “Old Copper”

North America’s copper-using peoples of the Archaic Period were initially classified together as one broad technological phenomenon that was referred to by a variety of designations such as the “Old Copper Culture” [21, 48, 49] the “Old Copper Complex” [23, 50], or the “Old Copper Industry” [51, 52]. Regardless of naming convention, the “Old Copper” phenomenon was formally defined by the presence of large utilitarian copper tools which were forged from native copper and found in the Great Lakes region [15].

In recent decades, researchers have delineated other culturally and historically distinct uses of copper and now generally consider Old Copper to be the first cultural manifestation of groups linked by copper tool technology, both utilitarian and decorative. The age range for Old Copper is generally thought to extend from approximately 6780–3100 cal BP [15] (dates [14] calibrated using IntCal20). This suggests that the Old Copper traditions and the earliest copper use in the mortuary contexts began in the Middle Archaic (ca. 8000–5000 BP) and may have extended into the Late Archaic (ca. 3000–1000 BP). The Old Copper temporal range was initially defined based on evidence from Reigh [21, 53], Osceola [20, 21] Oconto [21, 54], and Price III [55]. These four sites represent the type sites for early investigations of copper use in the western Great Lakes and provided the basis for the definition of Old Copper [23] as a specific set of early practices.

#### “Red Ocher”

Red Ocher sites are defined as a set of mortuary practices that are both more complex and found nearer to copper sources than earlier Old Copper mortuary practices. These sites are located throughout the Midwest, spanning Iowa and the Upper Mississippi valley in the west, across southern Michigan, and eastward into southern Ontario and Ohio. Red Ocher traditions were defined largely on a suite of burial practices that are in many ways similar to those of Old Copper sites but exhibit increased mortuary complexity. Specifically, Red Ocher cemeteries contain remains that have been covered with hematite powder, representing a distinctly new practice associated with societies using copper. These burials often include caches of burnt ceremonial bifacial blades and in some instances contain marine shell beads [41, 56]. In the upper western Great Lakes, Red Ocher mortuary contexts contain copper materials as grave offerings. Of interest is that these Red Ocher burial sites evince a shift in copper goods away from the large utilitarian tools that were regularly associated with Old Copper burials, towards smaller copper items that are interpreted as objects of personal ornamentation. In addition to the shift in copper forms, there was an overall increase in prestige goods, exemplified by the presence of worked marine shell, exotic cherts, and obsidian [41].

The Early Woodland tradition—delineated by the presence of burials in human-made mounds, the use of pottery, and the adoption of horticulture—appeared around 3000 BP in regions south of the Great Lakes. However, such patterns do not appear in the Upper Great Lakes until sometime after 2000 BP. Given the time-transgressive nature of material culture traditions, there is ambiguity in precisely how the Red Ocher practices fit into the Late Archaic-Woodland transition of the Upper Great Lakes and how these traditions articulated with those further south. Indeed, in some instances, late Red Ocher sites contain pottery and burials in human-made mounds [57, 58] both formal characteristics of Woodland traditions [15]. However, the majority of Red Ocher sites do not have ceramic materials or human-made mounds. As with the preceding Old Copper tradition, little is known regarding Red Ocher social organization or subsistence strategies as no habitation sites have been excavated. However, mortuary data has provided insight into emerging social complexity during this time as mortuary centers include a greater frequency of exotic goods and personal ornaments, many of which occur with infant and young adult female burials [41].

#### “Burnt Rollways”

The Burnt Rollways expression was first defined by Salzer [11] and has been further clarified with subsequent investigations [39, 59–61]. In contrast to other copper using traditions, which were defined primarily through their associations with particular mortuary programs, Burnt Rollways sites frequently contain evidence of copper tool production along with the associated debris [11, 39, 59].

**Fig 1:**
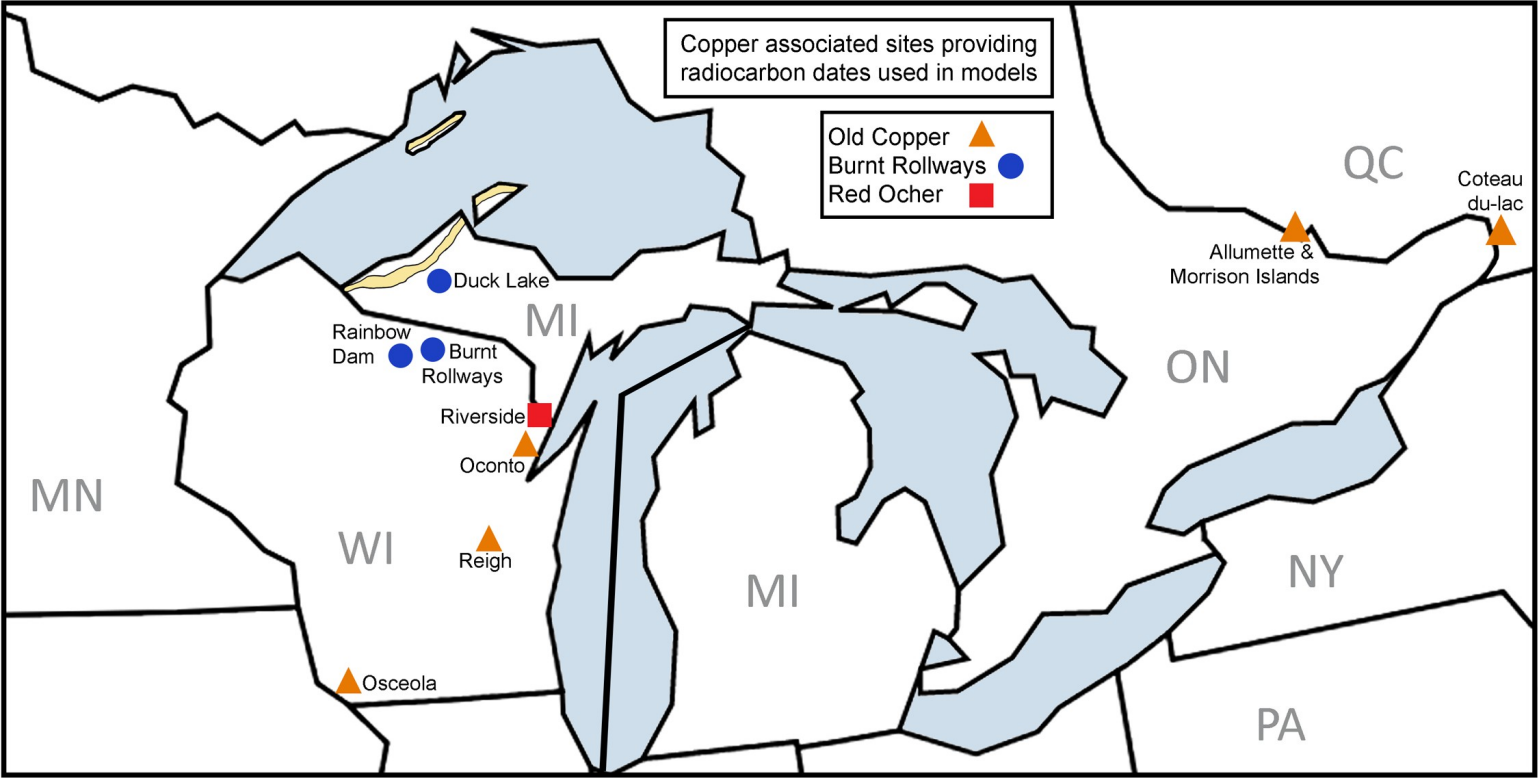
Map showing Archaic Period copper sites and the Lake Superior copper mining district. Radiocarbon dates from the archaeological sites shown here were used to generate age ranges for three Archaic Period copper-using traditions—Old Copper, Burnt Rollways, and Red Ocher. Republished from MapChart.net under a CC BY license, with permission from Minas Giannekas, original copyright 2021. Modified by MRB using Adobe Photoshop version 13 x64.

Although not nearly as well-known as the Old Copper or Red Ocher traditions, Burnt Rollways sites are vital for understanding the trajectory of cultural change and interaction among copper-using groups, as Burnt Rollways sites are located nearer the copper sources in the southern Lake Superior Basin (Fig 1). Likewise, in contrast to sites from Old Copper and Red Ocher, the Burnt Rollways sites are associated with evidence for habitation that provides insight into the lifeways of copper producers during the Late Archaic period–data that are lacking for the earlier Middle Archaic Old Copper components, where most data come from mortuary contexts several hundred kilometers away from the copper deposits. Burnt Rollways sites were situated farther north than both Old Copper and Red Ocher sites with many of the sites located near the headwaters of the Wisconsin, Wolf, Menominee, and Ontonagon Rivers [40].

Therefore, given the proximity of Burnt Rollways sites to copper sources, and their temporal position which may have overlapped with both the Old Copper and Red Ocher mortuary complexes, it is possible that the Burnt Rollways sites functioned as the distributional hub for copper during the Late Archaic to Woodland transition. However, to fully understand the relationships between copper procurement in the mining region and possible distribution to the southerly ceremonial centers, contemporaneity between these different kinds of sites must be established.

### Bayesian chronological modelling

Bayesian statistics are uniquely suited for the analysis of radiocarbon data because of their focus on probabilities. As extensive overviews of Bayesian analysis of radiocarbon dates have been published elsewhere [62–67] only a brief introduction is provided here and below. A more thorough review of the methodology, concepts, and terminology are also included with the S1 File. The results of “scientific dating are always interpreted contextually” and Bayesian statistics “provide an explicit, quantitative method which can combine raw dates with other prior information included in a model to produce formal statistical date estimates which combine both sets of evidence” [63]. In the following case, radiocarbon determinations represent likelihoods. The association of radiocarbon dates with each of the three distinct kinds of copper-bearing archaeological sites represent our prior beliefs. The critical assumption made here by grouping radiocarbon dates by particular practices (identified through their associations with particular tool technologies, settlement patterns, and mortuary traditions) is that these resulting groups can be used to then model the starts, ends, and spans of these associated traditions and for each of the three kinds of copper-bearing sites. Bayesian techniques allow us to formally produce such temporal estimations in place of “eye-balling” large radiocarbon datasets. Importantly, we employ formal techniques to assess the order and contemporaneity of temporal events (e.g., the starts and ends of the three copper-using traditions) rather than through less rigorous, visual assessments of radiocarbon datasets.

## Data and methods

The dataset used here (Table 1) includes available radiocarbon dates associated with three distinct kinds of copper-bearing sites or traditions associated with the use of copper: Old Copper (n = 21), Burnt Rollways (n = 5), and Red Ocher (n = 16). A total of 42 dates from 11 sites associated with these three traditions are used to build a series of chronological models. Radiocarbon dates were used only from sites that contained copper artifacts or evidence of copper working. Additionally, 34 radiocarbon dates from 21 sites associated with copper, but not specifically associated with any of three sets of traditions explored here, were also used to investigate the total dataset of copper-associated radiocarbon dates from the region. Of the 76 measurements, 32 were made on charcoal, 17 on unidentified wood or wood bark, 13 on human bone, 4 on unidentified animal bone, 4 on cordage or plant fiber, 3 on unidentified charred wood, 2 on leather, and 1 on subcarbonized organic material. Of the 76 available dates, 9 dates were excluded for having error ranges over 150 years. This is an arbitrarily defined cut-off, but most of the dates excluded exceeded error values between 250 and 600. Though these dates were excluded from inclusion in the primary models, all models were also run with these dates and are included as alternative models in the S1 File.

**Table 1:** Radiocarbon dates used in this study. All dates come from contexts directly associated with copper.

Lab ID #	Affiliation	Site Name	RCYBP	Range +/-	Context of material dated	Material Dated	Primary Source
AA19677/ WG2403 [41]	Red Ocher	Riverside	2780	65	Burial (Feature 14S: bark fragment directly associated with an obsidian grave offering and copper beads)	Wood (bark fragment)	Pleger 2000
AA19678/ WG2404 [41]	Old Copper	Oconto	6020	60	Embedded (Feature 1; cordage; string fragment adhered to copper crescent in direct association with burial)	Cordage (plant fibers)	Pleger 2000
AA19679/ WG2405 [41]	Red Ocher	Riverside	2960	50	Burial (Feature 29H; cremation pit; copper beads in burial pit)	Charcoal	Pleger 2000
AA19680/ WG2406 [41]	Red Ocher	Riverside	2790	50	Burial (Feature 13H; bundle burial; copper celt and beads in burial pit)	Charcoal	Pleger 2000
AA19681/ WG2407 [41]	Red Ocher	Riverside	2380	50	Burial (Feature 37; cremation burial pit; copper celt found in burial feature)	Charcoal and bark	Pleger 2000
AA19682/ WG2408 [41]	Red Ocher	Riverside	2605	45	Burial (Feature 45H; flexed burial; 2 copper awls in burial pit)	Carbonized organic material	Pleger 2000
AA19683/ WG2409 [41]	Red Ocher	Riverside	2605	50	Burial (Feature 3H; burial pit; cremation and bundle burial together in same feature; copper beads found with cremation; birch bark mat under the bundle burial)	Wood (bark fragment)	Pleger 2000
AA19684/ WG2410 [41]	Red Ocher	Riverside	2710	50	Burial (Feature 31H; burial pit with multiple burials and charred wood; copper beads found in pit)	Charcoal	Pleger 2000
AA19685/ WG2411 [41]	Red Ocher	Riverside	2850	50	Burial (Feature 27H; flexed burial; copper pike with burial)	Charcoal	Pleger 2000
AA19686/ WG2412 [41]	Red Ocher	Riverside	2690	60	Burial (Feature 41H; flexed burial; copper fragments with burial)	Charcoal	Pleger 2000
AA20281/WG2413 [41]	Old Copper	Oconto	5250	110	Burial (Feature 10; extended burial; copper awl adjacent to burila; copper artifacts associated with burial pits and throughout mortuary area)	Charcoal	Pleger 2000
AA20282/ WG2414 [41]	Red Ocher	Riverside	2495	65	Embedded (Feature 14S; leather strip used to string copper beads; found in burial pit with obsidian and copper grave goods)	Cordage (leather string)	Pleger 2000
Beta-099777 [39]	Burnt Rollways	Duck Lake	3420	50	Stratigraphic (Feature A; charcoal from 24 cm below surface; hearth containing worked copper nuggets and tools)	Charcoal	Hill 2006
Beta-124454 [39]	Burnt Rollways	Duck Lake	3400	110	Stratigraphic (charcoal from 17 cm below surface; feature containing copper scraps and anvil; associated with copper manufacturing)	Charcoal	Hill 2006
Beta-134256 [85]	NA	Eagle Lake	7690	40	Embedded (wood; shaft preserved inside copper conical point; isolated find; found 4–6 inches below surface on shore of Eagle Lake)	Wood	Reardon 2014
Beta-141985 [86]	Old Copper	Allumette Island	5440	80	Burial (burial pit 4; found in unit 12E-F; copper conical point in copper cache; grave goods with burial)	Bone (human)	Clermont, Chapdelaine, & Cinq-Mars 2003
Beta-141986 [86]	Old Copper	Allumette Island	5270	40	Burial (burial pit 7; found in unit 11A-B; copper conical point in copper cache; grave goods with burial)	Bone (human)	Clermont, Chapdelaine, & Cinq-Mars 2003
Beta-141987 [86]	Old Copper	Allumette Island	4680	40	Burial (burial pit 11; found in unit 12M; copper conical point in copper cache; grave goods with burial)	Bone (human)	Clermont, Chapdelaine, & Cinq-Mars 2003
Beta-215300 [87]	Old Copper	Morrison Island—6	4820	40	Burial (burial pit 11; extended burial with copper axe; faunal remains found at bottom of burial pit)	Bone (faunal)	Pilon and Young 2009
Beta-215301 [87]	Old Copper	Morrison Island—6	4210	40	Burial (burial pit 7; located in unit 6-A; extended burial with copper knife, awl, and gouge; faunal remains found at bottom of burial pit)	Bone (faunal)	Pilon and Young 2009
Beta-215302 [87]	Old Copper	Morrison Island—6	4730	40	Burial (burial pit 19; burial with copper bracelets; faunal remains found at bottom of burial pit)	Bone (faunal)	Pilon and Young 2009
Beta-232440 [61]	Burnt Rollways	Burnt Rollways	2280	40	Stratigraphic (Test pit 5; level B; copper artifacts at site)	Charcoal	Hill 2011
Beta-24330/88-3-51 [81]	NA	20KE20	1570	100	Embedded (Test unit 4, Feature 1, 15–25 cm below surface; Leather in direct contact with copper mass)	Leather	Martin 1993
Beta-243582 [61]	NA	Burnt Rollways	1540	40	Stratigraphic (Test pit 4; Level B; copper artifacts at site)	Charcoal	Hill 2011
Beta-247459 [61]	Old Copper	Reigh	4490	40	Embedded (wood; shaft material preserved inside copper conical point; found with Reigh burial 26)	Wood (embedded)	Hill 2011
Beta-29787/88-3-49 [81]	NA	20KE20	3300	60	Stratigraphic (Test unit 10, Level 2, 10–20 cm below surface in firepit)	Charcoal	Martin 1993
Beta-29788/88-3-56 [81]	NA	20KE20	7870	350	Stratigraphic (Test unit 12, Feature 1, 31cm below surface, worked copper at same level as charcoal sample)	Charcoal	Martin 1993
Beta-29789/88-3-93 [81]	NA	20KE20	3260	70	Stratigraphic (Test unit 50, Level 2, 15 cm below surface)	Charcoal	Martin 1993
Beta-343669 [88]	NA	Sandy Lake Dam Site	5690	30	Stratigraphic (Feature 10; hearth/refuse pit; contained 6 pieces of copper)	Bone (faunal)	Bradford 2017
Beta-370311 [82]	NA	Grace Peninsula	3730	30	Stratigraphic (charcoal found in association with worked copper)	Charcoal	Pompeani et al. 2021
Beta-485561 [89]	NA	Big Bend Site	3350	30	Embedded (wood; shaft material preserved inside copper socketed tang projectile point; isolate found in Rusk Co, WI)	Wood	Morris and Steinbring 2020
Beta-492176 [89]	NA	Taylor 1	5730	30	Embedded (wood; shaft material preserved inside copper socketed tang projectile point; isolate found in Rusk Co, WI)	Wood	Morris and Steinbring 2020
Beta-511973 [82]	NA	Pine River	7310	30	Embedded (wood; shaft material preserved inside copper conical point; isolate found in Florence Co, WI)	Wood	Pompeani et al. 2021
Beta-511974 [82]	NA	Wisconsin River	1770	30	Embedded (wood; shaft material preserved inside copper conical point; isolate found in Oneida Co, WI)	Wood	Pompeani et al. 2021
Beta-511975 [82]	NA	Wisconsin River	6380	30	Embedded (wood; shaft material preserved inside copper conical point; isolate found in Vilas Co, WI)	Wood	Pompeani et al. 2021
Beta-511976 [82]	NA	Lac Vieux Desert	6900	30	Embedded (wood; shaft material preserved inside copper conical point; isolate found near North Shore of Lac Vieux Desert, MI)	Wood	Pompeani et al. 2021
Beta-511977 [82]	NA	Lake Michigamme	3680	30	Embedded (wood; shaft material preserved inside copper conical point; isolate found on North Shore of Lake Michigamme)	Wood	Pompeani et al. 2021
Beta-88725 [90]	Old Copper	Morrison Island—6	4620	40	Burial (burial pit 7; located in unit 6-A; extended burial with copper knife, awl, and gouge)	Bone (human	Clermont and Chapdelaine 1998
Beta-88851 [90]	Old Copper	Morrison Island—6	4860	50	Burial (burial pit 11; extended burial with copper axe)	Bone (human)	Clermont and Chapdelaine 1998
Beta-88852 [90]	Old Copper	Morrison Island—6	4630	40	Burial (burial pit 19; burial with copper bracelets)	Bone (human)	Clermont and Chapdelaine 1998
C-836 [91]	Old Copper	Oconto	5600	600	Burial (Feature 1, Area 2; cremation pit; copper spatula with burial)	Charcoal	Libby 1954
C-837/C-839 [91]	Old Copper	Oconto	7510	600	Burial (Features 3 & 11, Area 1; burial pit containing flexed, bundled, and extended burials; copper artifacts associated with burial pits and mortuary area)	Charcoal	Libby 1954
CAMS-174540 [82]	NA	Kane Tool	4345	30	Embedded (cordage attached to copper conical point; isolate found in Vilas Co, WI)	Cordage	Pompeani et al. 2021
GAK [92]	Old Copper	Oconto	4540	400	Burial (Feature 15; flexed burial; human bone sample; copper artifacts associated with burial pits and mortuary area)	Bone (human)	Ritzenthaler 1970
GSC-162 [93]	Old Copper	Morrison Island—6	4700	150	Burial (burial pit 17; contained two copper bracelets and a copper knife)	Charcoal	Kennedy 1967
M-1275c [94]	NA	Lookout Mine	2800	120	Stratigraphic (Mining pit 56; charcoal from prehistoric copper mining pit; found between 3.2–3.4 feet below surface; located on Greenstone Ridge, Isle Royale, MI)	Charcoal	Crane and Griffin 1964
M-1275d,e,f,g[94]	NA	Lookout Mine	4110	130	Stratigraphic (Mining pit 56; charcoal from prehistoric copper mining pit; found between 3.4–5.1 feet below surface; located on Greenstone Ridge, Isle Royale, MI)	Wood	Crane and Griffin 1964
M-1384 [95]	NA	Minong Mine	4420	150	Stratigraphic (Mining pit, Trench 2; charcoal found with hammerstones 5.0–5.5 feet below surface; Prehistoric copper mine on Isle Royale, MI)	Wood	Crane and Griffin 1965
M-1385 [95]	NA	Minong Mine	3360	130	Stratigraphic (Mining pit 76; charcoal from 8 feet below surface in prehistoric copper mining pit; found with hammerstones; pit located near McCargoe Cove, Isle Royale, MI)	Charcoal	Crane and Griffin 1965
M-1386 [95]	NA	Siskiwit Mine	3370	130	Stratigraphic (Mining pit 66; charcoal from prehistoric copper mining pit; found with small grooved hammerstones; pit located on Isle Royale, MI)	Charcoal	Crane and Griffin 1965
M-1387 [95]	NA	Minong Mine	3220	130	Stratigraphic (Mining pit 77; charcoal from thin dark stratum 4.3 feet below surface in prehistoric copper mining pit; located near McCargoe Cove, Isle Royale, MI)	Charcoal	Crane and Griffin 1965
M-1388 [95]	NA	Minong Mine	3460	130	Stratigraphic (Mining pit 77; charcoal found with hammerstones 3.0–4.3 feet below surface; Prehistoric copper mine on Isle Royale, MI)	Charcoal	Crane and Griffin 1965
M-1389 [95]	NA	Minong Mine	3310	130	Stratigraphic (Mining pit 74; charcoal from 1.5 feet below surface in prehistoric copper mining pit; located near McCargoe Cove, Isle Royale, MI)	Charcoal	Crane and Griffin 1965
M-1390 [95]	NA	Minong Mine	4400	150	Stratigraphic (Mining pit, Trench 4; charcoal found with hammerstones between 2.6 to 6.8 feet below surface; prehistoric copper mine on Isle Royale)	Charcoal	Crane and Griffin 1965
M-1715 [96]	Red Ocher	Riverside	1949	130	Burial (Feature 37; burial pit; charcoal from burnt logs; >500 copper beads with flexed burial and copper celt)	Charcoal	Hruska 1967
M-1716 [96]	Red Ocher	Riverside	2050	130	Burial (Feature 46; possible burial pit; copper awl fragment found 58 inches below surface; pit contained stratifed sand and six projectile points; charred logs were present in pit)	Charcoal	Hruska 1967
M-1717 [96]	Red Ocher	Riverside	2190	140	Burial (Feature 27; burial pit containing flexed burial a copper pike; charred birch bark covering over burial)	Charred wood (bark)	Hruska 1967
M-1718 [96]	Red Ocher	Riverside	2080	140	Burial (Feature 30; burial pit containing 3 flexed burials and a cache of 13 hornstone blades; adjacent to Feature 21 containing 4 copper points, 2 copper crescents, and 2 copper awls)	Charred wood	Hruska 1967
M-1719 [96]	Red Ocher	Riverside	2460	140	Burial (Feature 31; burial pit containing multiple burials and charred wood; copper beads found in pit)	Charcoal	Hruska 1967
M-320 [97]	NA	Minong Mine	3000	350	Stratigraphic (Mining pit; charred spruce; found 70 inches below surface in a prehistoric copper mining pit near McCargoe Cove on Isle Royale, MI)	Charred wood	Crane 1956
M-371e [97]	NA	Minong Mine	3800	500	Stratigraphic (Mining pit; charred log; found between 11–12 feet below surface in a prehistoric copper mining pit near McCargoe Cove, Isle Royale)	Charcoal	Crane 1956
M-643 [98, 99]	Old Copper	Osceola	3450	250	Burial (human bone from bundle burial; copper artifacts directly associated with burial; mortuary layer was 2.5–5 ft below surface)	Bone (human)	Crane and Griffin 1959; Ritzenthaler 1958
M-644 [98]	Old Copper	Reigh	3660	250	Burial (human bone from burial in gravel layer; copper artifacts found throughout gravel in mortuary area)	Bone (human)	Crane and Griffin 1959
M-658 [100]	Red Ocher	Riverside	3040	150	Burial (Feature 6; burial pit contained flexed burial along with 3 copper projectile points)	Bone (human)	Papworth 1967
S-1263 [101]	Old Copper	Coteau-du-Lac	5000	80	Burial (burial pit 9G49B; contained a flexed burial with a copper filament and copper fragments)	Bone (human)	Marois 1987
S-509 [102]	Old Copper	Allumette Island	5240	80	Burial (burial pit AX-6; located in unit 12W; 5 copper artifact in unit)	Bone (human)	Kennedy 1970
TO-2213 [103]	NA	Renshaw #1	4590	50	Embedded (plant fiber; bark binding adhered to barbed copper harpoon point; isolate from the Renshaw site)	Cordage (plant fiber-bark)	Beukens et al. 1992
TO-2215 [103]	NA	Renshaw #3	4630	60	Embedded (plant fiber; grass-based twined cordage adhered to copper toggle harpoon; isolate found at the Renshaw site)	Cordage (plant fiber-grass)	Beukens et al. 1992
TO-2216 [103]	NA	Anderson #1	5940	90	Embedded (wood; splinter from shaft material preserved inside copper projectile point tang; isolate found near South Fowl Lake, MN)	Wood	Beukens et al. 1992
TO-2441 [103]	NA	Renshaw #4	4420	60	Embedded (wood; shaft material preserved inside copper projectile point tang; isolate found at the Renshaw site)	Wood	Beukens et al. 1992
UCIAMS-190517 [82]	NA	N. Washington Harbor	2235	15	Embedded (wood shaft inside conical copper point; isolate found on Isle Royale)	Wood	Pompeani et al. 2021
W-291 [104]	NA	Minong Mine	3310	200	Stratigraphic (Mining pit; charred log; found 13 feet below surface in a prehistoric copper mining pit near on Isle Royale, MI)	Charcoal	Rubin and Suess 1956
WG613 [85]	NA	Eagle Lake	7305	60	Embedded (wood shaft of copper conical point; isolated find; found 4–6 inches below surface on shore of Wisconsin River)	Wood	Reardon 2014
WIS-1706 [99, 105]	Old Copper	Osceola	4080	70	Burial (charred human bone; possibly from a partial cremation; copper artifacts found throughout burial layer 2.5–5 ft below surface)	Bone (human)	Steventon & Kutzbach 1986; Ritzenthaler 1958
WIS-2269 [59]	Burnt Rollways	Rainbow Dam East	3630	60	Stratigraphic (Feature 3, Unit A, Level 4; basin shaped pit containing 1 copper awl and 3 copper scraps)	Charcoal	Moffat and Speth 1999
WIS-2270 [59]	Burnt Rollways	Rainbow Dam West	3270	80	Stratigraphic (Feature B, Unit E, Level 5; basin shaped pit; copper scraps, copper awls, and copper knife fragments were found in Unit E)	Charcoal	Moffat and Speth 1999

It is important to acknowledge that the dataset of available radiocarbon dates is relatively small given the geographic and temporal ranges expected for the cultural practices investigated here. While a small sample size does represent a constraint on the confidence of model outputs, the Bayesian framework employed here actually provides a more formal, empirical assessment of chronological placement given the currently available data. In contrast to approaches that merely "eye-ball" temporal ranges and chronological trends, Bayesian approaches provide a formal framework for assessing probabilities, even from small datasets. In fact, the model structures provided here, and in the S1 File, might serve as a framework for incorporating both new radiocarbon dates and new prior information as either becomes available. Additionally, the simulations we describe below are meant to offer full transparency on the potential effects of our small sample sizes on model outputs and on the potential for our models to change as new information is added. In sum, regarding sample size, the models presented here represent the best and most empirical chronologies and temporal ranges that can be estimated given currently available data and provide a formal framework for future chronological investigations.

Three separate models, one for radiocarbon dates associated with each of the three sets of traditions, were constructed independently of one another. Dates associated with the same kind of copper-associated sites were grouped first by site and then in an overall phase representing the practice of particular traditions (e.g., Old Copper). Each of the three models (Old Copper, Burnt Rollways, and Red Ocher) incorporated trapezium boundaries to estimate start and end boundaries. Trapezium boundaries are commonly used to model cultural phenomena that have an unknown length of period across which they began, persisted, and ended [68–71]. Trapezium boundaries allow for varied temporalities of adoption, use, and abandonment of particular traditions or practices. In general, they represent a more flexible set of modeling parameters compared to other kinds of boundaries (e.g., sigma boundaries; see Alternative Model D in the S1 File for outputs produced using sigma boundaries for comparison). A charcoal outlier model was applied to dates obtained from charcoal to account for potential discrepancies between unidentified wood charcoal dates within the model [72]. Additionally, a general outlier model was applied to all non-charcoal dates to identify any potential outliers and to down weight those determinations that exhibit heightened potentials to be outliers.

In addition to the primary models described above, a series of alternative models were included as a form of sensitivity analysis. By altering model parameters, we were able to assess the effects of different modeling decisions on chronological estimations and to assess the propensity for the model to change given different parameters. The results of the Bayesian modeling efforts (modeled starts, ends, and spans) represent the posteriors. All models were built using OxCal v 4.4 [65, 73] and the IntCal20 calibration curve [74]. Full descriptions and OxCal code for all primary, alternative, and simulated models are included as S1 File.

An additional step was taken using simulations to determine the representativeness of our own radiocarbon dataset and to model chronological scenarios given an ideal dataset [64, 75–79]. As such, in addition to the results gleaned from our radiocarbon dataset, we simulated how the results might change with the addition of a more robust set of radiocarbon dates. To do this, we ran a number of simulations to determine the number of dates needed to produce models that 1) exhibit high levels of precision in start and end boundary determinations and that 2) do not change with the addition of more radiocarbon dates. The full procedures and methods of determinations are presented in the S1 File.

## Results

**Fig 2:**
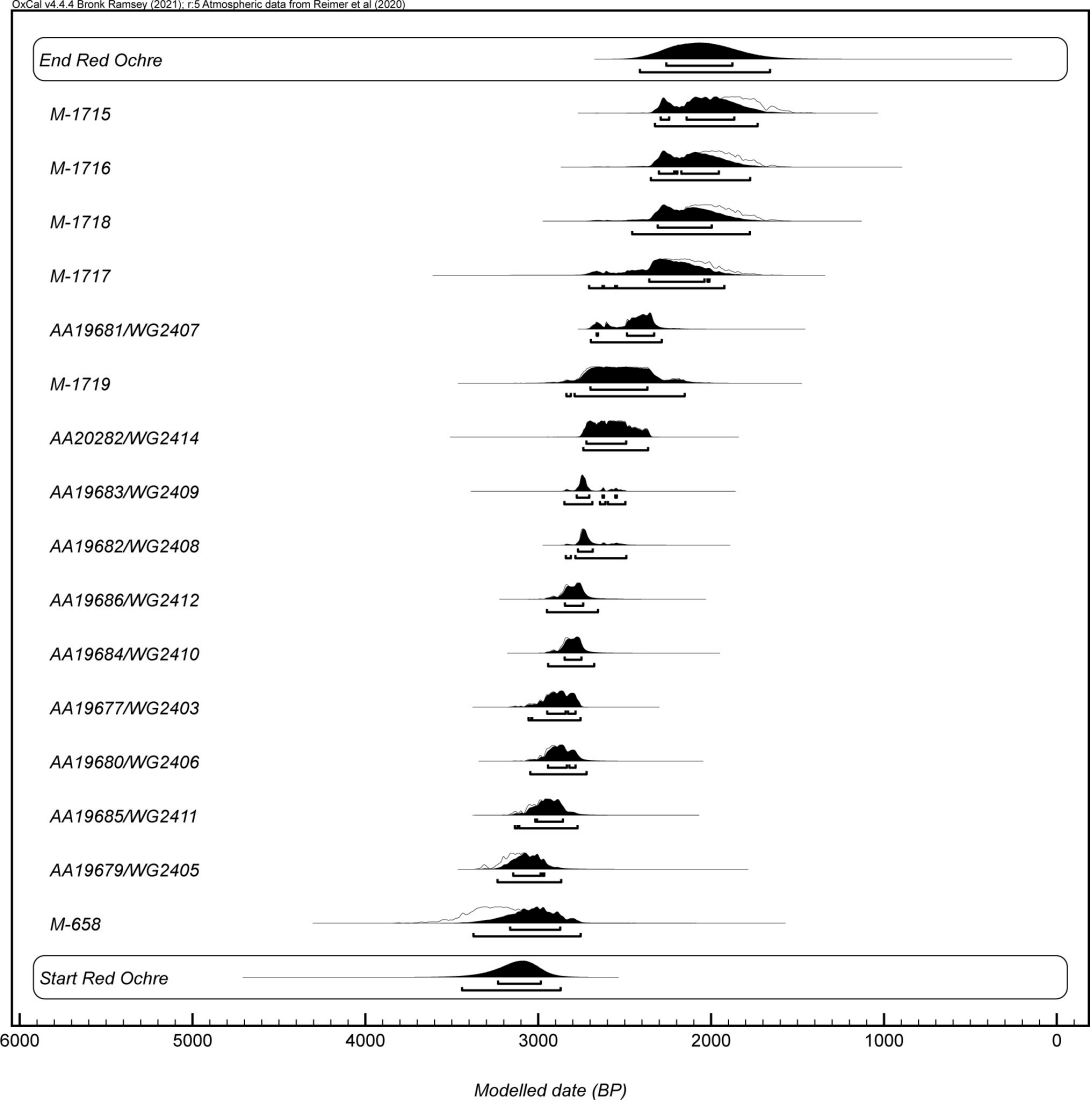
OxCal plot of modeled Red Ocher dates along with start and end boundaries.

**Fig 3:**
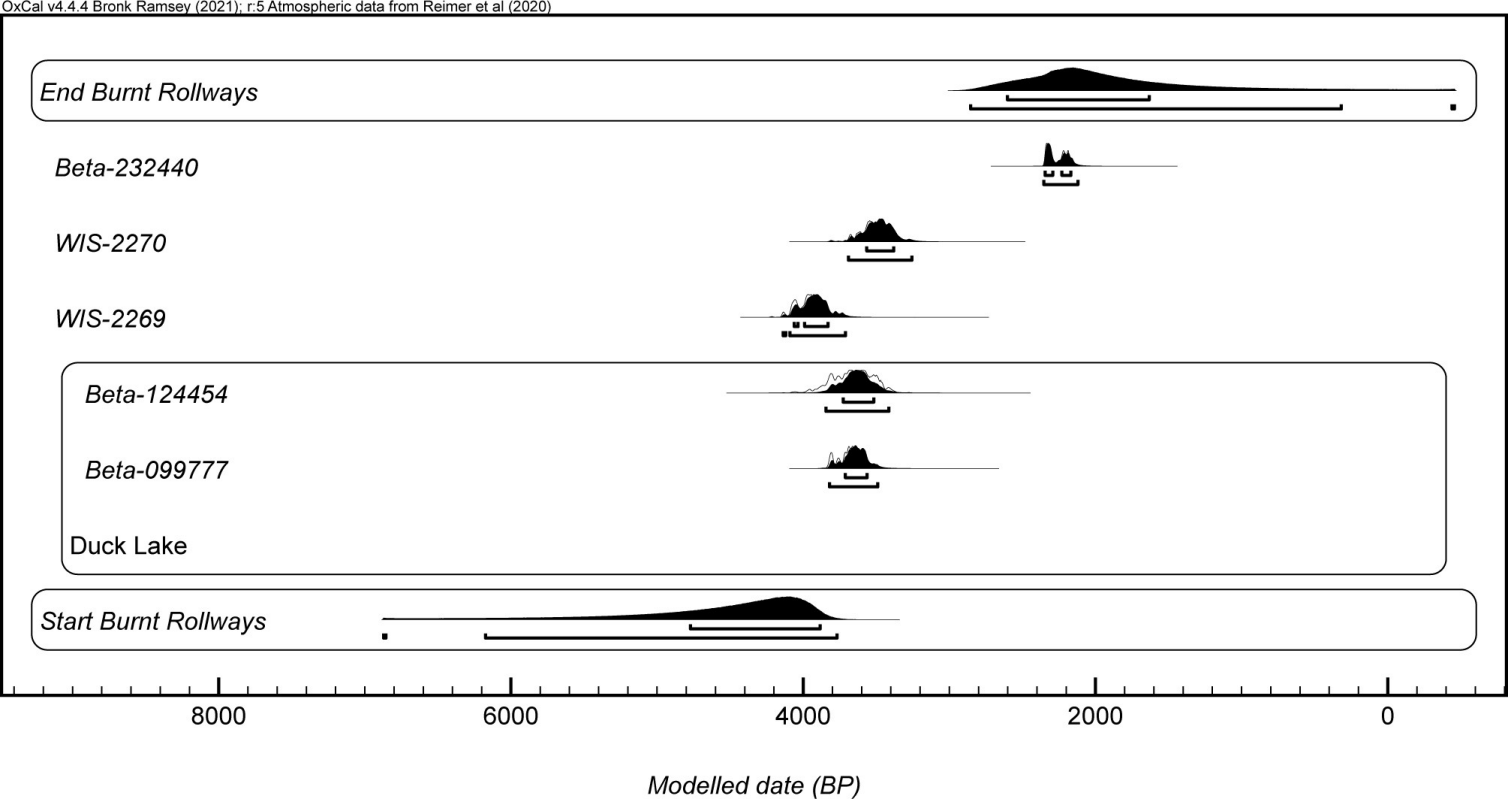
OxCal plot of modeled Burnt Rollways dates along with start and end boundaries.

**Fig 4:**
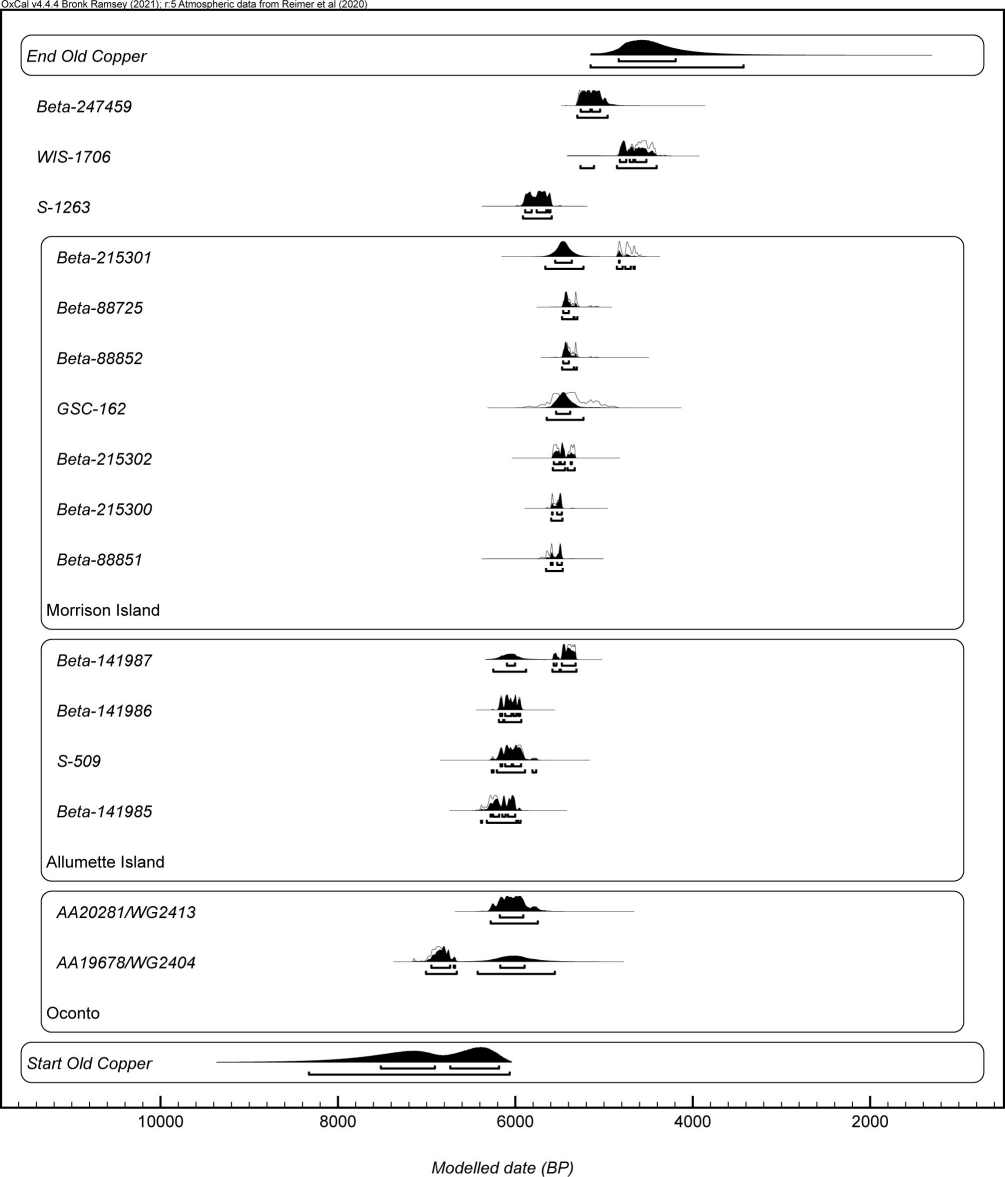
OxCal plot of modeled Old Copper dates along with start and end boundaries.

**Fig 5:**
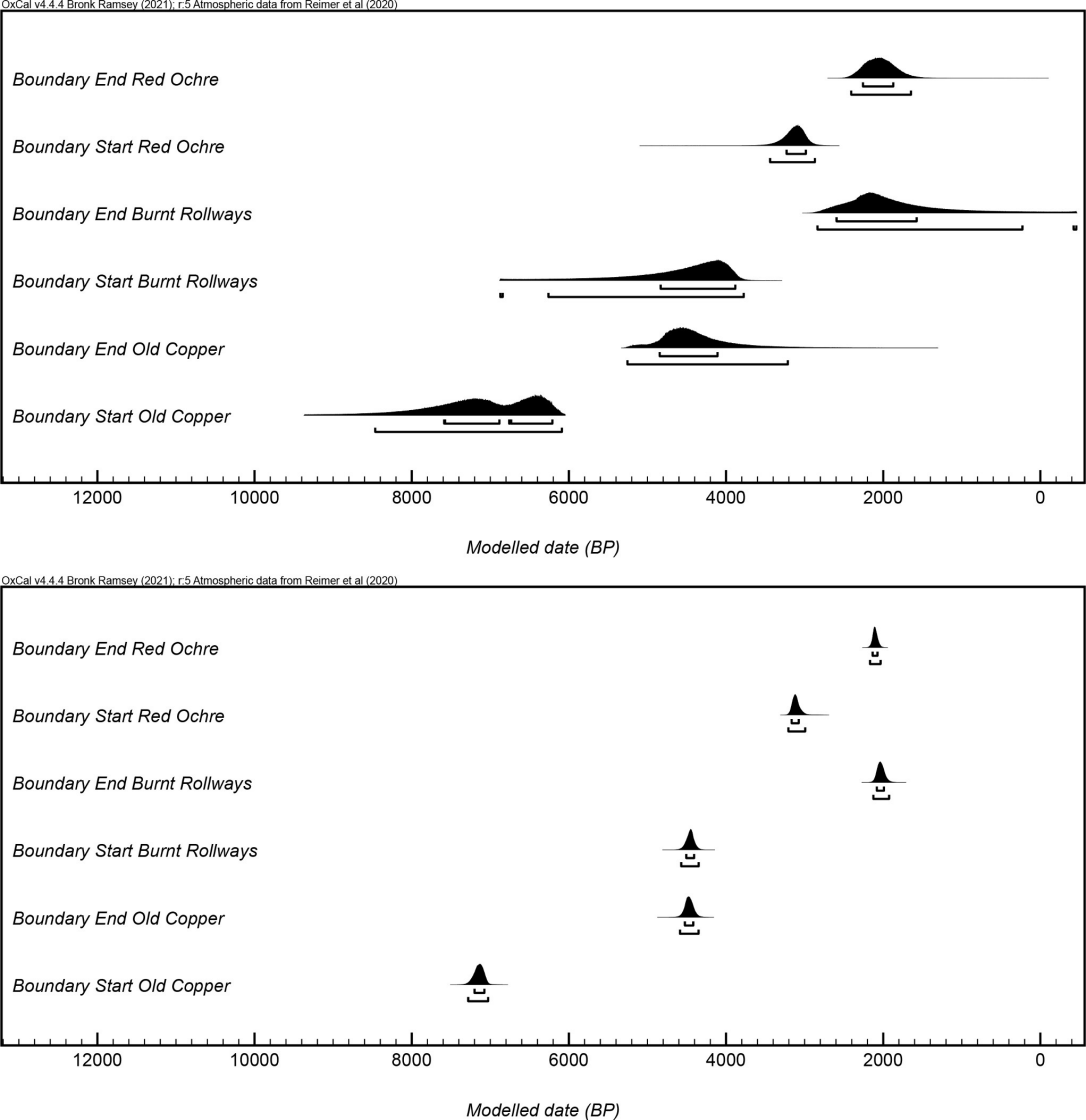
Modeled start and end boundaries from both the primary models (top) and simulated models (bottom) for Old Copper, Burnt Rollways, and Red Ocher.

**Fig 6:**
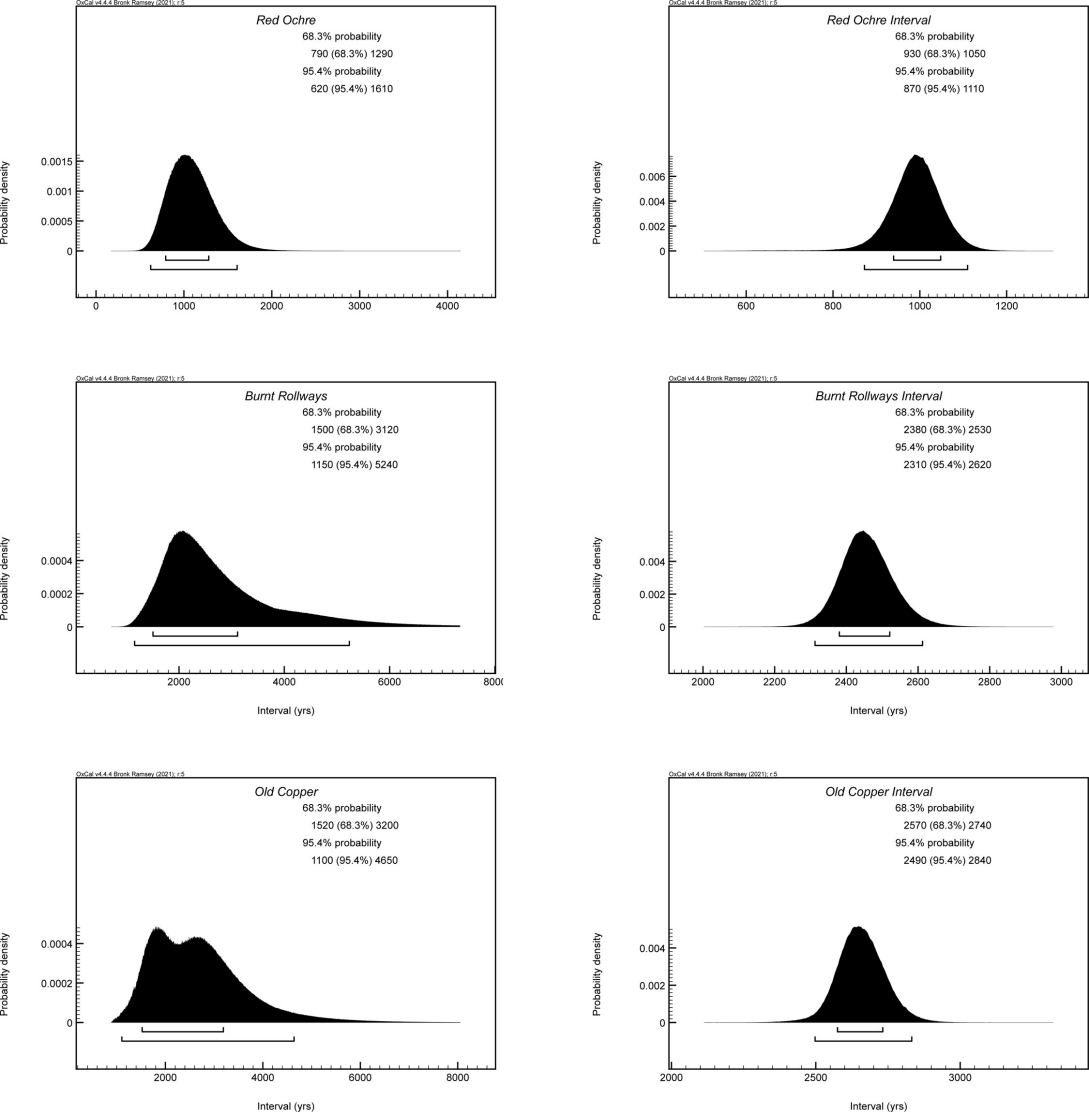
Modeled spans (produced using the interval command) from both the primary models (left) and simulated models (right) for Old Copper, Burnt Rollways, and Red Ocher.

**Fig 7:**
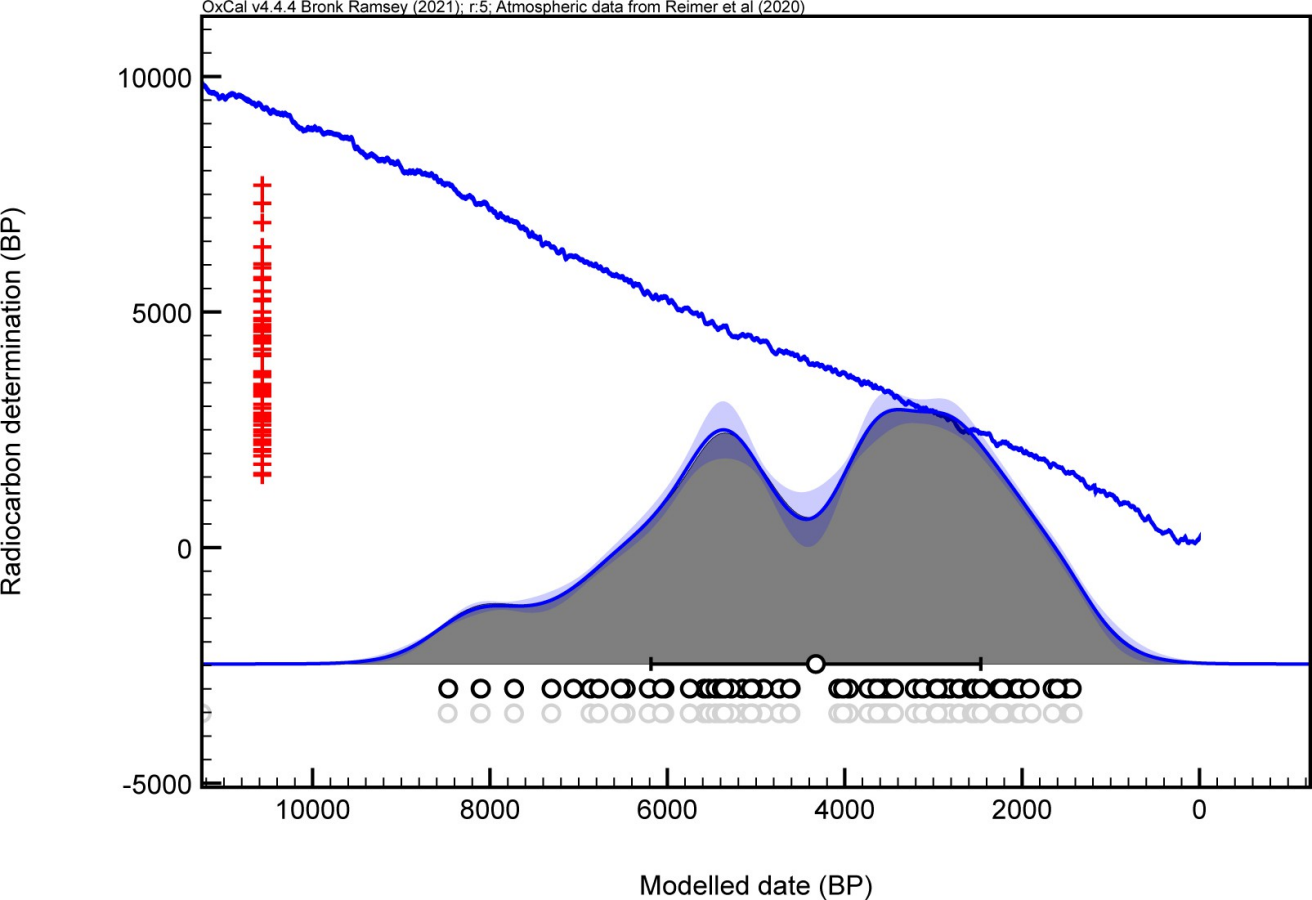
Kernel density estimate plot for all 76 radiocarbon dates associated with copper from across the region.

The three primary models were used to produce start boundary ranges, end boundary ranges, and spans for Old Copper, Burnt Rollways, and Red Ocher (Figs 2–7). The results at both the 68% and 95% confidence intervals are presented in Table 2. Modeled results in text are cited at the 68% confidence interval and presented in italics. Old Copper traditions are estimated to have begun between *cal BP 7520–6180*, ended between *cal BP 4840–4190*, and spanned a length of *1*,*520–3*,*200 years*. Burnt Rollways is estimated to have begun between *cal BP 4780–3880*, ended between *cal BP 2610–1630*, and spanned a length of *1*,*500–3*,*120 years*. Red Ocher is estimated to have begun between *cal BP 3240–2980*, ended between *cal BP 2260–1870*, and spanned a length of *790–1*,*290 years*.

**Table 2:** Modeled start boundaries, end boundaries, and spans for each of the three cultural traditions produced through both the primary and simulated models.

	*68%*	95%	Simulated 68%	Simulated 95%	Most recent published dates
Start Old Copper (*cal BP*)	7520–6180	8330–6060	7200–7070	7290–7020	6950 [15]
End Old Copper (*cal BP*)	4840–4190	5160–3420	4530–4410	4590–4350	2950 [15]
Interval Old Copper (*years*)	1520–3200	1100–4650	2570–2740	2490–2840	4000
Start Burnt Rollways (*cal BP*)	4780–3880	6880–3760	4510–4400	4570–4340	3640 [61]
End Burnt Rollways (*cal BP*)	2610–1630	2860-AD 460	2090–1990	2130–1920	1540 [61]
Interval Burnt Rollways (*years*)	1500–3120	1150–5240	2380–2530	2310–2620	2100
Start Red Ocher (*cal BP*)	3240–2980	3450–2870	3170–3070	3210–2990	3450 [15]
End Red Ocher (*cal BP*)	2260–1870	2420–1650	2140–2070	2170–2030	2350 [15]
Interval Red Ocher (*years*)	790–1290	620–1610	930–1050	870–1110	1100

The order command in OxCal was used to statistically determine the appropriate sequence of start and end boundaries for each of the three traditions (Table 3). While it is quite clear that Old Copper practices began far earlier than both Burnt Rollways and Red Ocher practices, the timing for the end of Old Copper and the beginning of the two other traditions remains ambiguous. The probability that Old Copper practices ended before the start of Red Ocher practices is high. There is, however, just a 45% chance that Old Copper practices ended before the start of Burnt Rollways practices. Similarly, the temporal relationship between the copper working and habitation Burnt Rollways sites and the mortuary Red Ocher sites is ambiguous. While it is clear Burnt Rollways sites begin to be used before Red Ocher practices develop, it is difficult to determine whether or not Red Ocher mortuary traditions outlast the habitation and tool-working Burnt Rollways sites. It may be the case that habitation sites, copper-processing sites, and the Red Ocher mortuary practices all cease at roughly the same time.

**Table 3:** Probability matrices for the primary and simulated models.

Probability matrices							
		Start Old Copper	End Old Copper	Start Burnt Rollways	End Burnt Rollways	Start Red Ochre	End Red Ochre
**Primary Models**	Start Old Copper			1	1	1	1
	End Old Copper			0.45	1	1	1
	Start Burnt Rollways	0	0.55			1	1
	End Burnt Rollways	0	0			0	0.45
	Start Red Ochre	0	0	0	1		
	End Red Ochre	0	0	0	0.55		
**Simulated Models**	Start Old Copper			1	1	1	1
	End Old Copper			0.6	1	1	1
	Start Burnt Rollways	0	0.4			1	1
	End Burnt Rollways	0	0			0	0.1
	Start Red Ochre	0	0	0	1		
	End Red Ochre	0	0	0	0.9		

Probability matrices illustrating the likelihoods that the events listed as rows occurred before the events listed as columns (e.g., there is a 60% probability that the Old Copper Complex end boundary occurred before the Burnt Rollways start boundary).

To increase the precision of potential chronological placements based on available information and model parameters, a simulation experiment was undertaken to determine how many dates would be needed to resolve some of these sequencing issues and to achieve a more robust chronological framework that would be unyielding to new radiocarbon data. The full description of these procedures is reported in the S1 File along with all the OxCal code for each of the simulated models. In short, the modeled chronological ranges produced using the primary models were used to produce simulated chronological ranges. To do so, simulated dates that fell within the ranges produced through the primary models were added until the modeled age estimates 1) ceased to substantively change with new data and 2) provided a precision for start and end boundaries of less than 200 years. It should be noted that these simulated results are representative of actual observations only if new observations (dates) indeed follow the trends identified in the currently available datasets. New radiocarbon dates that fall substantively earlier or later than currently available dates would more substantively change estimated chronological boundaries. The simulated results here thus represent a scenario consistent with extant datasets and currently available observations.

Given the length of these temporal periods and their association with particular parts of the radiocarbon calibration curve, it was determined that ca. 90 additional radiocarbon dates for each of the three traditions (a total of 270 additional radiocarbon dates) would be necessary for achieving a desired precision and representativeness. The point of this exercise is not necessarily to call for such a program of data collection, but rather to create an environment of transparency and to explore the limitations of the currently available dataset [78]. The simulation results should be used as a hypothetical baseline against which to assess the representativeness and robustness of the radiocarbon dates that do exist. Importantly, the only experiments run here were those that added more dates. Further simulations that build in stratigraphic relationships between radiocarbon dates, radiocarbon dates from more sites, or new contextual associations between dates could certainly produce representative models that necessitate far fewer than 270 dates. Nevertheless, the simulated models provide refined ranges for each of the three traditions and lend insight into potential scenarios of their temporal relationships to one another. The results of these simulations are presented next to the results of the primary models in Figs 5 and 6 and in Table 2. Using these simulations based on currently available information, Old Copper is simulated to have begun between *cal BP 7200–7070*, ended between *cal BP 4530–4410*, and spanned a length of *2*,*570–2*,*740 years*. Burnt Rollways is simulated to have begun between *cal BP 4510–4400*, ended between *cal BP 2090–1990*, and spanned a length of *2*,*380–2*,*530 years*. Red Ocher is simulated to have begun between *cal BP 3170–3070*, ended between *cal BP 2140–2070*, and spanned a length of *930–1*,*050 years*.

Once again, the order command in OxCal was used to statistically determine a simulated sequence of start and end boundaries for each of the three traditions (Table 3). It remains clear that the Old Copper practices began much earlier than the other two sets of practices. There is still ambiguity as to whether Burnt Rollways sites began to be occupied before Old Copper practices ceased, with there being a 60% chance that Old Copper practices ended before the start of Burnt Rollways occupations. This likely indicates a potential scenario in which the end of Old Copper mortuary practices and tool-making traditions are contemporaneous with the beginning of Burnt Rollways habitation sites. In any case, Old Copper practices ended, and Burnt Rollways sites began to be occupied, before the Red Ocher traditions began to develop. However, Red Ocher mortuary practices and copper-working traditions certainly began before Burnt Rollways sites were all abandoned, indicating that the two overlap significantly in time. In fact, there is a 90% chance that the end of Red Ocher practices preceded the end of Burnt Rollways practices, suggesting that the entirety of the Red Ocher tradition was contemporaneous with Burnt Rollways habitation and copper-working sites.

It is important to reiterate, however, that the simulations are constrained by what can be gleaned from the extant dataset of radiocarbon dates. As such, new data could certainly alter these results. New radiocarbon dates that fall within the maximum and minimum bounds produced using the current dataset will likely not have a substantive effect on results and may potentially match the results produced through simulations. New dates that fall beyond the minimum and maximum ranges represented by the extant dataset may be more likely to substantively alter the results presented here. This caveat may be most relevant to the timing of Burnt Rollways, where the current dataset consists of just five radiocarbon dates associated with copper materials. The likelihood of obtaining new radiocarbon determinations that expand the range of Burnt Rollways thus remains high. That said, the current dataset of Burnt Rollways dates (five dates from four sites) does represent a potentially robust set of dates that produce generally unchanging results when modeled multiple times, exhibiting little variance in model results over many runs (e.g., modeled boundaries vary by less than 200 years over ten runs; see S1 File for a test of variance for Burnt Rollways modeled boundaries). Unfortunately, Burnt Rollways sites, representing relatively inconspicuous practices like ephemeral habitations and copper-working/processing, are likely least represented both spatially and temporally of the three kinds of sites explored here. While these five dates from four sites consistently produce similar modeled results, new archaeological work may likely produce a wider temporal range for such sites than presented here.

All 76 available radiocarbon dates were also used to produce a kernel density estimate plot. The purpose of the KDE plot is simply to assess the overall distribution of the dataset. The plot itself represents the distribution of dates along a temporal gradient (the means of those dates are represented as white circles below the x-axis in Fig 7). Kernel density estimates take into consideration the frequency of observations (radiocarbon dates), not simply the raw sum of their probability distributions, as in the case of summed probability distribution plots [80]. In this way, a plot is produced that represents the probability of a radiocarbon date at a specific point in time. Such frequentist approaches like kernel density estimates have routinely been used for spatial analyses to estimate the probability that something will occur at a specific place given the frequency of observations of that thing occurring there and elsewhere.

The 76 dates associated with copper from across the region span a great deal of time from ca. 8500 cal BP to ca. 250 cal BP. In the KDE plot presented here (Fig 7), there are three conspicuous features, two broad peaks and a subtle valley between them. The first peak is centered at ca. 5500 cal BP, the second peak at ca. 3300 cal BP, and the valley spanning roughly 500 years between 4400 and 3900 cal BP. It is clear that these peaks are associated with dense clusters of radiocarbon dates whereas the valley is associated with a paucity of dates between these two dense clusters. Whether or not this gap is real, or a product of sampling and archaeological coverage, remains unclear, but its investigation may present fruitful directions for future research.

## Discussion

The results of the models reveal several thought-provoking possibilities, and in some ways, introduce more questions than they can fully answer. For example, although copper use likely began, at least in small scale, by Late Paleoindian times [1], Bayesian models show that a unifying cultural tradition does not become evident until sometime after 8000 years ago. This is not to say that shared traditions involving copper did not begin earlier, but simply that, based on current evidence, the first archaeologically identifiable cultural manifestation linked by an abundance of utilitarian copper tools is that of Old Copper traditions. Indeed, the results of the Bayesian analyses suggest that Old Copper practices began and ended substantially earlier than previously thought and also had a much shorter span (Table 2). These modeled results are informative, however, many questions remain. In particular, given that there are so few early dates from archaeological sites located near the copper sources [81], it is still uncertain precisely who conducted the earliest copper mining in this region. Nor is it fully understood how or why the predominance of copper tools have been found in areas far removed from the copper mines, while there is a paucity of copper tools found near the mining sources. This gap in our understanding is exacerbated by the ephemerality of archaeological materials that could provide insight into the lifeways of Early-Middle Archaic groups in the upper Great Lakes.

Based on the available archaeological data, we suggest that during the Middle Archaic, groups with modes of mortuary practices involving the inclusion of large, utilitarian copper tools (“Old Copper”) made seasonal trips to copper sources for procurement, and then may have returned south to the larger mortuary sites as part of their seasonal rounds. If these groups were small and mobile, it would explain the relative lack of habitation sites during the Early-Middle Archaic period in northern Wisconsin and the Upper Peninsula of Michigan. As successfully argued by others, evidence suggests that during the Middle Archaic, long-distance, task-specific movements were the norm, and groups engaged in such task-specific activities are nearly archaeologically invisible [16].

Alternatively, we acknowledge it is possible that there were larger scale habitation sites in the mining district, possibly even antecedent to the Burnt Rollways sites, that simply have not yet been found. Of interest is that the dates modeled here push the initial start date of copper-bearing habitation sites and tool-working sites (“Burnt Rollways”) back by nearly a millennium (Table 2). It is certainly possible given the age range for Burnt Rollways sites, that these practices developed from Old Copper traditions. However, the modeled age ranges show that, even though there was possibly temporal overlap, Burnt Rollways sites were not contemporary with the apogee of Old Copper practices. Thus, due to temporal disparities, it is unlikely that these northern Burnt Rollways sites, would have been involved in the distribution of copper to the Old Copper mortuary centers to the south. Though, once again, this temporal disparity may be the product of a paucity of dated habitation and tool-working sites more generally, and future work may reveal a more intensive temporal overlap between the Old Copper mortuary practices and these habitation sites located near copper resources.

Considering the geographic location it is possible that the Burnt Rollways sites represent an entirely different group of copper users in the northern region—one that is currently not well understood—who were focused on copper production and possibly distribution to other Late Archaic communities and groups in the surrounding region. Certainly, it is quite possible that Burnt Rollways is a later manifestation of an Early-Middle Archaic group who had inhabited the upper Great Lakes area but is currently unknown from the material record. Given that there were at least two groups with distinct practices coexisting in the area during the Late Paleoindian/Early Archaic transition, it is possible that these groups persisted in the area as separate entities who eventually formed and maintained their own unique traditions. Of interest to untangling these possible historical relationships, is evidence showing that as early as Late Paleoindian times, cremation was being practiced as a mortuary tradition [5] in the upper Great Lakes, possibly by people who later practiced communal mortuary ritual evinced in the Middle-Late Archaic Periods. Perhaps Late Paleoindian groups, who were the first to begin using native copper, developed *in situ* cultural traditions focused on copper mining and production, which eventually became archaeologically visible as Burnt Rollways sites. In fact, although habitation sites are largely missing, the earliest dated copper isolates [1, 82] are found in the northern regions close to the mining districts, and these copper projectiles date to the Early-Middle Archaic Period. Likewise, in the Keweenaw mining district, lead pollution from native copper processing first appears around 8000 cal BP but then decreases dramatically after 5000 cal BP [83], just prior to the initial occupations of Burnt Rollways sites.

According to the Bayesian models, Red Ocher traditions in the copper region only spanned a millennium. Given that Red Ocher mortuary practices and copper-working traditions began after the initial occupations of Burnt Rollways sites and was largely contemporaneous with these sites, multiple scenarios are possible regarding their potential relationships.

It is reasonable to conclude that there may have been relationships based on exchange of materials between peoples producing Burnt Rollways sites and peoples engaging in the complex Red Ocher mortuary practices. Or it could simply be that these various site types represent one group of people who frequented temporary production sites and small seasonal habitation sites, but were ultimately linked by a larger mortuary complex such as that at the Riverside site.

Regarding peaks in radiocarbon dates as gleaned from the KDE plot (Fig 7), the first peak at 5500 cal BP likely represents a period when copper was being extensively mined, processed, and used for a wide variety of large, utilitarian items by groups who are unfortunately not yet fully understood. Of interest is that by the second peak at 3300 cal BP, evidence for intensive copper mining has largely ceased [83]. A possible interpretation is that there were no longer seasonal trips to the mining district for large-scale copper extraction and production efforts. Unfortunately, the precise reasons for this change are currently unknown. It is possible that groups became more regionally restricted around 4500 years ago, at the same time that Old Copper mortuary practice and tool-making styles were disappearing from cultural repertoires. Limited evidence, in the form of a stone projectile found embedded in a human vertebra at the Price III site [55], suggests intergroup violence and competition were factors affecting human lifeways by the end of the Middle Archaic. Indeed, increasing regionalization and territorialism could also explain the disparities in material culture between Burnt Rollways and Red Ocher sites [40].

Likewise, if those participating in Old Copper practices who had previously practiced high levels of seasonal mobility—heading north in the warmer months to exploit aquatic resources and extract copper, and then south to mortuary centers and rockshelter hunting camps during cooler months—could no longer do this, subsequent groups would be forced to rely on one of two options for acquiring copper 1) sourcing “float” copper from local glacial till, and/or 2) recycling. Likewise, change in mobility patterns could also explain shifts in copper tool forms. If groups partaking in the complex mortuary practices and social traditions of the Red Ocher variety could no longer source large amounts of copper from the north, they would be forced to rely on recycling of copper material from large Early-Middle Archaic copper tools to produce new copper items. It follows that, recycling copper materials would ultimately lead to smaller artifacts being made from copper (i.e. awls and beads), consistent with Red Ochre traditions’ preference for smaller, ornamental copper pieces, which happen to be the two most common types of copper artifacts to persist into the Late Pre-contact Period [44, 45]. Additionally, as access to copper became more restricted and the raw material became less readily available, the social value of copper may have increased due to the scarcity principle [84]. This in turn may have intensified the prestige value placed on copper goods, which could explain the subsequent proliferation in copper ornaments after 3000 BP and shaped the social institutions and practices that defined the Red Ocher traditions [41].

## Conclusion

The trajectory of native copper use in the western Great Lakes of North America is a fascinating topic with global significance, however, it is also a topic that presents unique challenges. Nevertheless, these problems can be solved via the application of new techniques in dating, modeling, paleoenvironmental research, and experimental archaeology. Here, in an effort to better understand the timing of the various social, cultural, and economic practices associated with copper-use that occurred throughout the Archaic Period, and to elucidate the potential interrelationships between the copper-using groups involved in these expressions, we provide a revised and updated chronology via Bayesian modeling. The updated age ranges inform our understanding of the historical relationships between, and cultural interactions among, North America’s earliest copper-using groups. Unlike previous work focusing on fine-scale sequences of copper use at particular localities, the research presented here has been situated within a macroscale perspective to better temporally situate the peoples involved in the production, use, and exchange of copper materials across a socially, culturally, and historically complex landscape.

## Supporting information

S1 File Supplementary material file including alternative models, simulations, terminology, and code. (PDF)
