# Supplementary material for: Refining the chronology of North America’s copper using traditions: A macroscalar approach via Bayesian modeling
Source: PLoS One. 2022 Apr 26;17(4):e0266908. doi: 10.1371/journal.pone.0266908 (PMC9041870; doi:10.1371/journal.pone.0266908)
Supplement: S1 File — (PDF) [file pone.0266908.s001.pdf]

SUPPLEMENTAL MATERIAL

Bayesian Chronological Modeling

Bayesian statistics allow us to “analyze new data we have collected about a problem in the context of our existing experiences and knowledge about that problem” (Bayliss 2007:75). By doing so, we can “arrive at a new understanding of the problem which incorporates existing understandings about the problem and our new data” (Bayliss 2007:75). To use the associated terminology, new data, or observations, can be referred to as ‘likelihoods.’ Existing experiences and knowledge are referred to as ‘prior beliefs’ or *a priori* information. The resulting understandings we achieve from incorporating our prior beliefs into the analysis of new data are understood to be ‘posterior beliefs.’ Bayesian statistics are uniquely situated for the analysis of radiocarbon data because of their focus on probabilities. As extensive overviews of Bayesian analysis of radiocarbon dates have been published by experts in the technique (e.g. Bayliss 2015, 2007; Bayliss et al. 2007; Bronk Ramsey 2009a; Buck et al. 1996; Whittle et al. 2011), only a brief introduction is provided here. The results of “scientific dating are always interpreted contextually” and Bayesian statistics “provide an explicit, quantitative method which can combine raw dates with other prior information included in a model to produce formal statistical date estimates which combine both sets of evidence” (Bayliss 2007:76). In the following case, radiocarbon determinations represent likelihoods. The association of radiocarbon dates and archaeological sites assigned to particular cultural traditions represent our posterior beliefs. The results of the Bayesian modeling efforts (defined temporal ranges and overlaps for regional cultural traditions) represent the posteriors. All models were built using OxCal v 4.4 (Bronk Ramsey 2021, 2009a) and the IntCal20 calibration curve (Reimer et al. 2020).

Adopted by researchers for use in archaeological applications over two decades ago (e.g. Buck et al. 1991, 1992, 1994, 1996; Christen 1994; Christen and Litton 1995; Christen et al. 1995), Bayes’ theorem can be expressed mathematically as follows:

$$p(t \mid y) \propto p(y \mid t)p(t)$$

Where t represents a set of parameters, y represents observations or measurements, p(y|t) is the likelihood, and p(t|y) is the posterior probability, or the probability of a given parameter set given the measurements and the priors (Bronk Ramsey 2009a:338). This is expressed in a simpler manner by Bayliss (2007:76) and reads as follows:

$$\frac{P(data \mid parameters)}{P(data)} \mathbf{X} P(parameters) = P(posterior \mid data)$$

Where the likelihood is determined by the probability of the data or observations given the set parameters and is proportional to the probability of the parameters themselves. The combination of these two, observations/measurements and prior information or beliefs is where the value of Bayesian statistical methods lie, especially in regards to interpreting radiocarbon data.

Because radiocarbon dates are actually measurements of isotopic ratios, in order to be read as proxies for calendrical dates they must be calibrated against an established calibration curve that reflects fluctuations in atmospheric carbon isotopes over time (see Scott and Reimer 2009 for a detailed overview; Reimer et al. 2020). The process of calibrating a radiocarbon

determination thus results in a probability distribution along which the actual calendrical age of the sample likely lies. The incorporation of prior information about these observations allows for a formal assessment of observations as well as a formal evaluation of the prior assumptions used to interpret the data. Thus, a formalized Bayesian model allows for the simultaneous, quantitative evaluation of both radiocarbon data and our assumptions about the archaeological record. Through these efforts, the probability distributions of radiocarbon determinations can be modeled using this prior information and may significantly enhance both the precision and accuracy of chronometric dating by producing modeled posterior probability distributions. Arguably the strongest prior information we have as archaeologists are the depositional environments from which radiocarbon data are. More general priors including culture-historic frameworks, ceramic sequences, settlement patterns, stone tool traditions, and documentary evidence can also be employed as prior information. When using more generalized prior information, the assumptions employed may serve as a working hypothesis on which the analysis is based (Bronk Ramsey 2009a:348).

Terminology and Commands

A more thorough discussion of the mathematical expressions underlying each of the parameters discussed below is presented by Bronk Ramsey (2009a:348). One of the simplest parameters to impose on a group of radiocarbon dates is their inclusion in a ***phase***. A phase is an unordered group of events. When dates are grouped in a phase, it is assumed that all dates within the group are equally likely to occur anywhere between the start and end boundaries of the phase. No information concerning order is assumed. For the grouping of dates into a phase to serve as an informative parameter (sensu Bayliss 2007), the phase must be given start and end ***boundaries***. The use of particular kinds of boundaries defines how events (dates) are distributed within the phase. The distributional parameters imposed by particular types of boundaries provide another set of informative parameters that will produce variation in model outputs.

In this study, ***trapezium boundaries*** were used. Whereas the use of the ***boundary*** command assumes a uniform distribution of observations within a phase, ***trapezium boundaries*** are used to account for the unknown temporalities of start and end events (Lee and Bronk Ramsey 2012). ***Trapezium boundaries*** include two transition parameters that allow for flexibility and "reflect archaeological situations in which start and end boundaries could be more realistically expressed by a transition period from a beginning to a peak, and a similar decline towards the end" (Higham et al. 2014: Supplemental 111). Thus, ***trapezium models*** are appropriate for evaluating the temporal ranges for stone tool traditions across a region that likely exhibit periods of transition that relate to their initial adoption and eventual abandonment as a valid technology.

Models can also be built by including multiple ***phases*** within a model and defining the relationships between those ***phases***. The primary model presented below incorporates the dates from individual sites into individual ***phases*** that correspond to each site within an overarching phase representing the regional culture. For example, all dates from the Oconto site were included in a single ***phase***. These individual sites representing the regional Copper Culture tradition were then included together in an overarching ***phase*** for the Copper Culture tradition which was given ***trapezium boundaries*** to model the start and end estimates for the regional Copper Culture tradition. Models were built in this way for both all three regional cultural traditions explored here. This method represents the primary modeling procedures.

Another set of concepts relevant to the current study that need defining are ***outliers*** and ***outlier models***. A full review of the kinds of ***outliers*** and ***outlier models*** that may be applied in Bayesian analyses for archaeological applications can be found in Bronk Ramsey (2009b). ***Outlier models*** are used to "determine whether there are problematic determinations that do not agree with the prior framework" (Higham et al. 2014: Supplemental 6). The model output is thus affected by the down-weighting of particular determinations based on the modeled fit of each ***outlier***. To assess the effects of these model parameters on outputs, the same model frameworks for many of the alternative models were run with and without the application of ***outlier models***.

Lastly, ***interval*** and ***order*** commands were included to estimate the length of the lifespan for each tradition and to determine the probabilistic order of particular events (e.g., did the Old Copper Complex end before the start of Red Ochre?). The ***interval*** command was used to produce an estimated length for each of the three cultural traditions. This command produces modeled ***intervals*** in years with associated credibility intervals.

In total, a primary modeling procedure, three alternative modeling procedures, and a complete simulation experiment were used to evaluate the timing, temporality, and sequence of the Old Copper, Burnt Rollways, and Red Ochre traditions. Although each alternative model represents alterations to prior information and model structures, all models produces comparable results for all outputs in question.

Primary Model

Three separate models, one for radiocarbon dates associated with each of the three cultural traditions, were constructed independently of one another. Dates associated with the same cultural tradition were grouped first by site and then in an overall phase representing the practice of particular traditions (e.g., Old Copper Complex). Each of the three models (Old Copper, Burnt Rollways, and Red Ochre) incorporated trapezium boundaries to estimate start and end boundaries. A charcoal outlier model was applied to account for potential discrepancies between unidentified wood charcoal dates within the model. Additionally, a general outlier model was applied to all dates to identify any potential outliers and to down weight those determinations that exhibit heightened potentials to be outliers. Radiocarbon determinations with ranges of error that exceed 150 years were excluded. Additionally, kiterations were set to 150 (compared to the default of 30) to achieve robust convergence values that indicate the most possible permutations were expressed.

Alternative Model A

Three separate models, one for radiocarbon dates associated with each of the three cultural traditions, were constructed independently of one another. Dates associated with the same cultural tradition were grouped first by site and then in an overall phase representing the practice of particular traditions (e.g., Old Copper Complex). Each of the three models (Old Copper, Burnt Rollways, and Red Ochre) incorporated trapezium boundaries to estimate start and end boundaries. No outlier models were incorporated and all dates regardless of error range were included.

Alternative Model B

Three separate models, one for radiocarbon dates associated with each of the three cultural traditions, were constructed independently of one another. Dates associated with the same cultural tradition were grouped first by site and then in an overall phase representing the practice of particular traditions (e.g., Old Copper Complex). Each of the three models (Old Copper, Burnt Rollways, and Red Ochre) incorporated trapezium boundaries to estimate start and end boundaries. A charcoal outlier model was applied to account for potential discrepancies between unidentified wood charcoal dates within the model. Additionally, a general outlier model was applied to all dates to identify any potential outliers and to down weight those determinations that exhibit heightened potentials to be outliers. All dates regardless of error range were included.

Alternative Model C

Three separate models, one for radiocarbon dates associated with each of the three cultural traditions, were constructed independently of one another. Dates associated with the same cultural tradition were grouped first by site and then in an overall phase representing the practice of particular traditions (e.g., Old Copper Complex). Each of the three models (Old Copper, Burnt Rollways, and Red Ochre) incorporated trapezium boundaries to estimate start and end boundaries. No outlier models were incorporated, but radiocarbon determinations with ranges of error that exceed 150 years were excluded.

Alternative Model D

This is the same as the Primary Model, except trapezium boundaries were replaced with sigma boundaries to define distributions. Three separate models, one for radiocarbon dates associated with each of the three cultural traditions, were constructed independently of one another. Dates associated with the same cultural tradition were grouped first by site and then in an overall phase representing the practice of particular traditions (e.g., Old Copper Complex). Each of the three models (Old Copper, Burnt Rollways, and Red Ochre) incorporated trapezium boundaries to estimate start and end boundaries. No outlier models were incorporated and all dates regardless of error range were included.

Simulation Experiments

The simulation experiments reported here are used to determine 1) at what point would the addition of new dates no longer affect model results and 2) how many dates would be needed to achieve a precision of less than 200 years in estimating start and end boundaries for each of the three cultural traditions. Results from the primary model were used to build simulations. For each cultural tradition, the means from the modeled start and end boundaries were used as ranges within simulated dates were generated and added to the models. For Old Copper, simulated dates were added in the range of 7050-4380 cal BP; for Red Ochre, simulated dates were added in the range of 3130-2040 cal BP; for Burnt Rollways, simulated dates were added in the range of 4600-1860 cal BP. All procedures for generating simulated dates and evaluating simulated models follow those outlined by Holland-Lulewicz and Ritchison (2021). Groups of 10 randomly generated simulated dates were added iteratively to the phases for each cultural tradition. This was done through the addition of 100 randomly simulated dates for each cultural tradition. Start and end boundaries were plotted in excel to determine when variation in results "leveled out"

and when an acceptable precision of less than 200 years was achieved (Figures S1-S3). For each cultural tradition, it was determined that the most robust solution was at least 90 extra simulated dates. At 90 dates, variation in results begins to become less pronounced as results become repeated with the addition of more dates. There is also diminishing returns in precision at this point.

Burnt Rollways Variance Test

The dataset for the Burnt Rollways sites consists of five dates from four sites. As this sample is small, the model was run ten separate times to explore the potential variance in model results from one run to the next. With such a small sample, it could be expected that separate runs using the same data and same model parameters could produce a wide range of modeled results (e.g., differences in boundaries and intervals). The results of ten runs however, vary insignificantly (Figure S4). For modeled start boundaries, maximum and minimum potential start dates varied by no more than 250 years at the greatest and in most cases modeled results were repeated across models. Mean ages for these start boundaries varied even less, at 100 years maximum between models. Similarly, end boundary variance exhibited a max difference of 200 years between model runs with most results replicated from model to model. The maximum difference between modeled mean end boundaries was 200 years. Thes results indicate that both the primary model and the simulated model presented in the main text are unyielding to statistical variances inherent in the extant data. This does not mean, of course, that new dates from more sites would not affect modeled outputs.

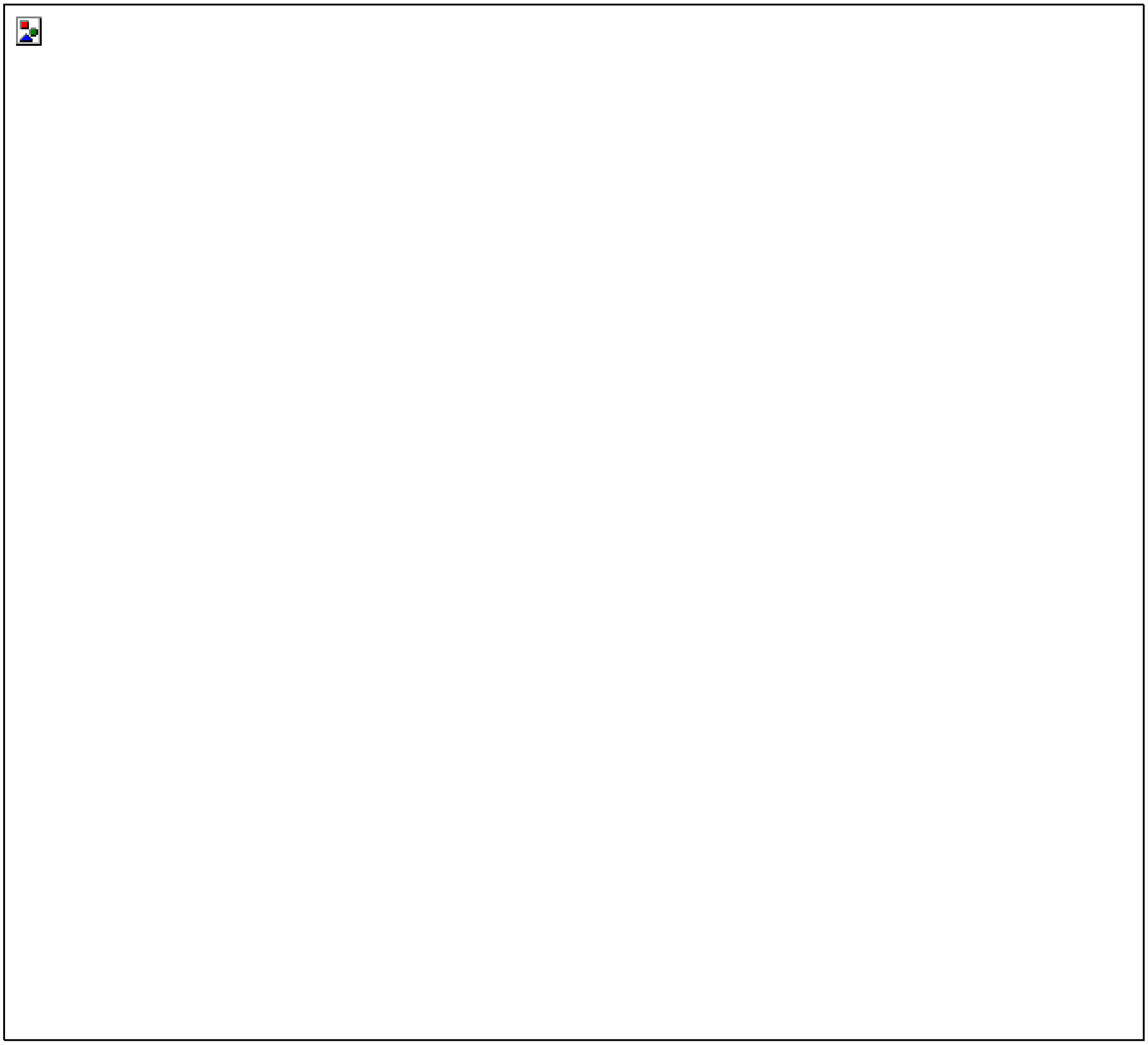

Figure S1. Simulation results for Red Ochre. The top plots depict modeled start and end boundaries across simulations; the middle plots depict the precision achieved for start and end boundaries across simulations; and the bottom plot depicts the modeled spans (using the interval command) across simulations.

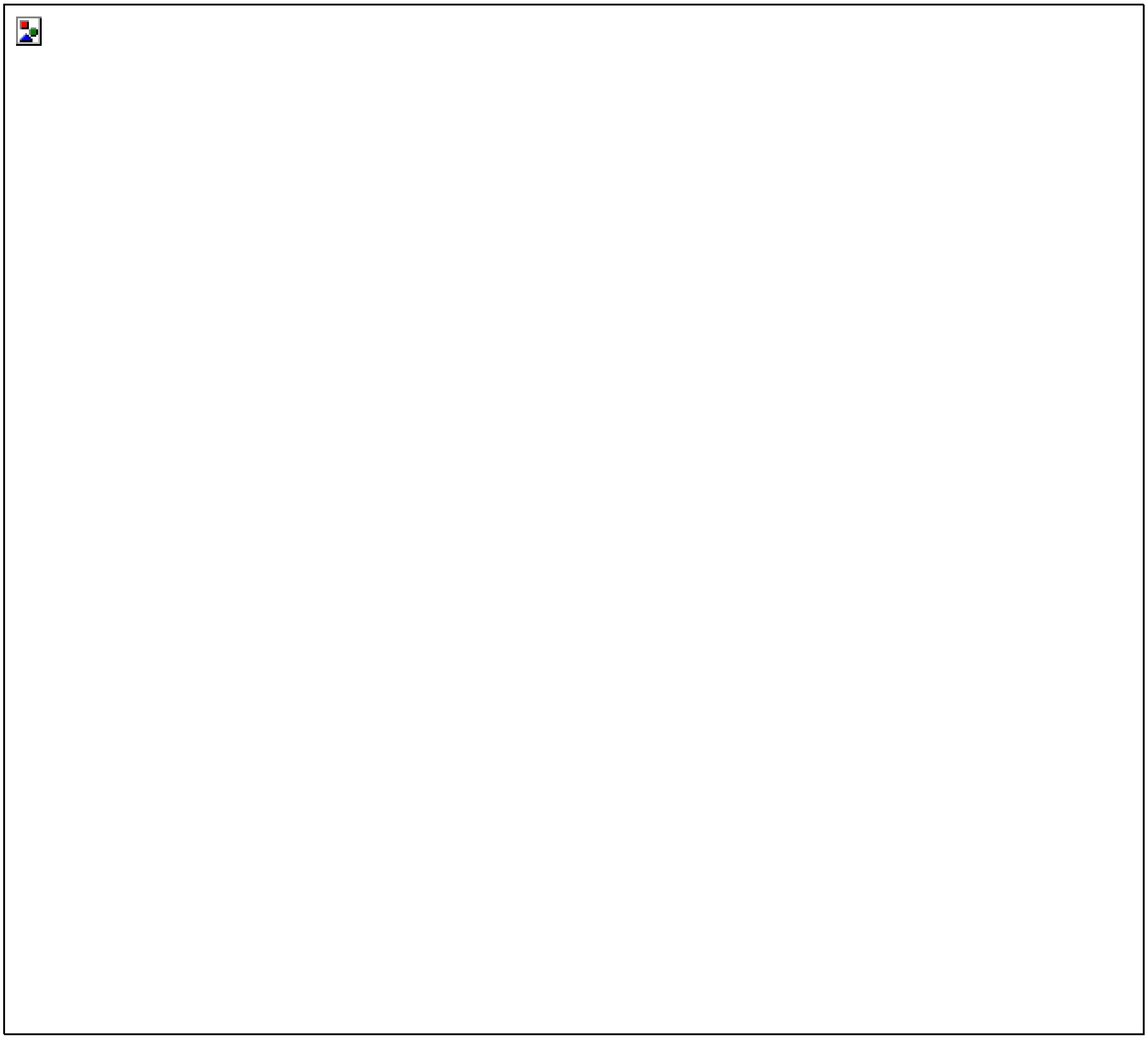

Figure S2. Simulation results for Old Copper. The top plots depict modeled start and end boundaries across simulations; the middle plots depict the precision achieved for start and end boundaries across simulations; and the bottom plot depicts the modeled span (using the interval command) across simulations.

Figure S3. Simulation results for Burnt Rollways. The top plots depict modeled start and end boundaries across simulations; the middle plots depict the precision achieved for start and end boundaries across simulations; and the bottom plot depicts the modeled span (using the interval command) across simulations.

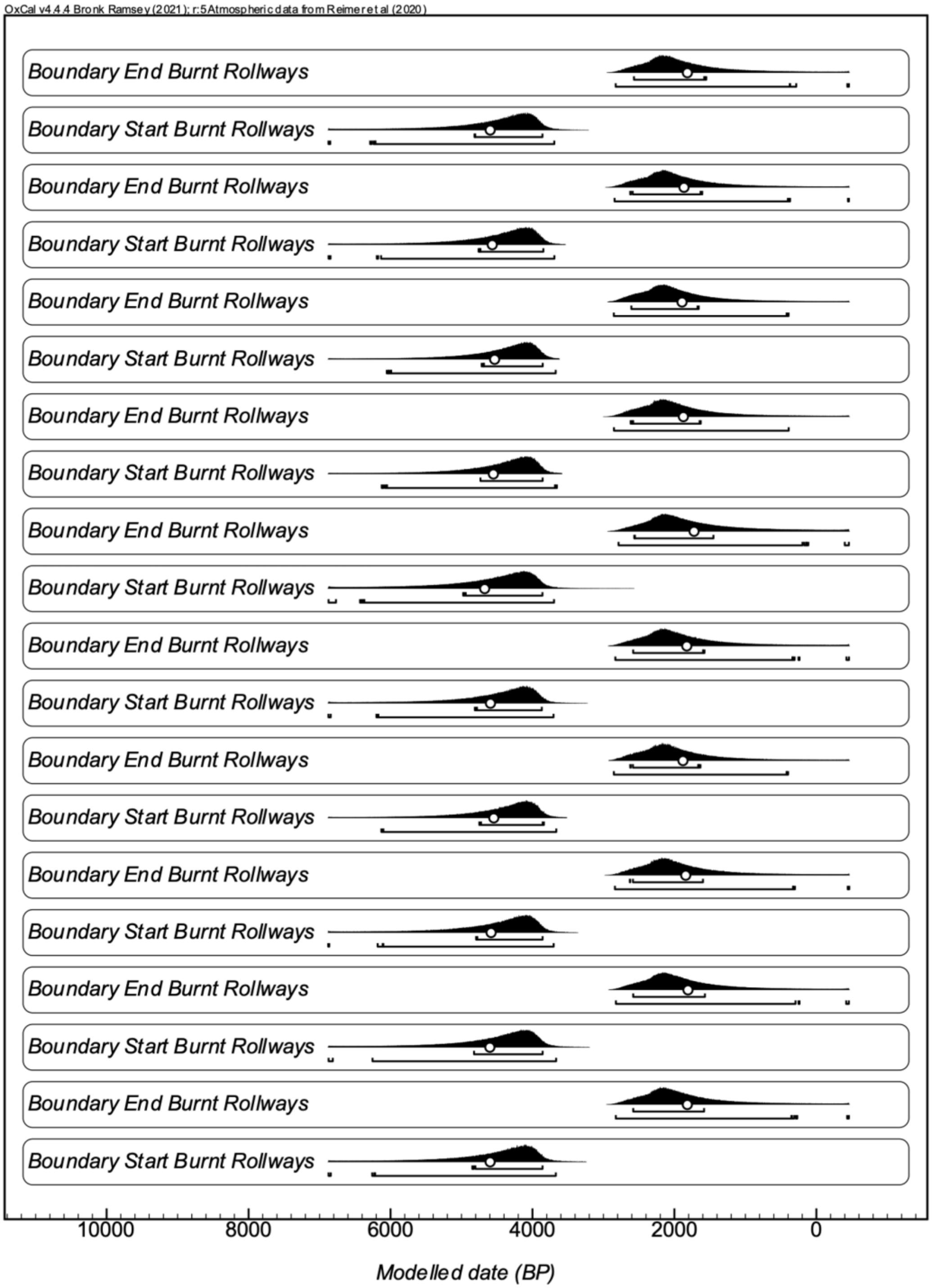

Figure S4. Modeled start and end boundaries over ten runs of the primary Burnt Rollways model.

Primary Model Code

```
Options()
 {
  kIterations=150;
 };
 Plot()
 {
  Outlier_Model("Charcoal",Exp(1,-10,0),U(0,3),"t");
  Outlier_Model("General",T(5),U(0,4),"t");
  Sequence("Red Ochre")
  {
   Boundary("Start Red Ochre")
   {
    Start("Start of Start Red Ochre");
    Transition("Period of Start Red Ochre");
    End("End of Start Red Ochre");
   };
   Phase("Red Ochre")
   {
    R_Date("M-658", 3040, 150)
    {
     Outlier("General", 0.05);
    };
    R_Date("AA19679/WG2405", 2960, 50)
    {
     Outlier("Charcoal", 1);
    };
    R_Date("AA19685/WG2411", 2850, 50)
    {
     Outlier("Charcoal", 1);
    };
    R_Date("AA19680/WG2406", 2790, 50)
    {
     Outlier("Charcoal", 1);
    };
    R_Date("AA19677/WG2403", 2780, 65)
    {
     Outlier("General", 0.05);
    };
    R_Date("AA19684/WG2410", 2710, 50)
    {
     Outlier("Charcoal", 1);
    };
    R_Date("AA19686/WG2412", 2690, 60)
    {
```

```
 Outlier("Charcoal", 1);
 };
 R_Date("AA19682/WG2408", 2605, 45)
 {
  Outlier("Charcoal", 1);
 };
 R_Date("AA19683/WG2409", 2605, 50)
 {
  Outlier("General", 0.05);
 };
 R_Date("AA20282/WG2414", 2495, 65)
 {
  Outlier("General", 0.05);
 };
 R_Date("M-1719", 2460, 140)
 {
  Outlier("Charcoal", 1);
 };
 R_Date("AA19681/WG2407", 2380, 50)
 {
  Outlier("Charcoal", 1);
 };
 R_Date("M-1717", 2190, 140)
 {
  Outlier("General", 0.05);
 };
 R_Date("M-1718", 2080, 140)
 {
  Outlier("Charcoal", 1);
 };
 R_Date("M-1716", 2050, 130)
 {
  Outlier("Charcoal", 1);
 };
 R_Date("M-1715", 1949, 130)
 {
  Outlier("Charcoal", 1);
 };
 Span("Red Ochre");
 Interval("Red Ochre");
};
Boundary("End Red Ochre")
{
 Start("Start of End Red Ochre");
 Transition("Period of End Red Ochre");
 End("End of End Red Ochre");
```

```
 };
};
Sequence("Old Copper")
{
 Boundary("Start Old Copper")
 {
  Start("Start of Start Old Copper");
  Transition("Period of Start Old Copper");
  End("End of Start Old Copper");
 };
 Phase("Old Copper")
 {
  Sequence()
  {
   Boundary("Start Oconto");
   Phase("Oconto")
   {
    R_Date("AA19678/WG2404", 6020, 60)
    {
     Outlier("General", 0.05);
    };
    R_Date("AA20281/WG2413", 5250, 110)
    {
     Outlier("Charcoal", 1);
    };
   };
   Boundary("End Oconto");
  };
  Sequence()
  {
   Boundary("Start Allumette Island");
   Phase("Allumette Island")
   {
    R_Date("Beta-141985", 5440, 80)
    {
     Outlier("General", 0.05);
    };
    R_Date("S-509", 5240, 80)
    {
     Outlier("General", 0.05);
    };
    R_Date("Beta-141986", 5270, 40)
    {
     Outlier("General", 0.05);
    };
    R_Date("Beta-141987", 4680, 40)
```

```
  {
   Outlier("General", 0.05);
  };
 };
 Boundary("End Allumette Island");
};
Sequence()
{
 Boundary("Start Morrison Island");
 Phase("Morrison Island")
 {
  R_Date("Beta-88851", 4860, 50)
  {
   Outlier("General", 0.05);
  };
  R_Date("Beta-215300", 4820, 40)
  {
   Outlier("General", 0.05);
  };
  R_Date("Beta-215302", 4730, 40)
  {
   Outlier("General", 0.05);
  };
  R_Date("GSC-162", 4700, 150)
  {
   Outlier("Charcoal", 1);
  };
  R_Date("Beta-88852", 4630, 40)
  {
   Outlier("General", 0.05);
  };
  R_Date("Beta-88725", 4620, 40)
  {
   Outlier("General", 0.05);
  };
  R_Date("Beta-215301", 4210, 40)
  {
   Outlier("General", 0.05);
  };
 };
 Boundary("End Morrison Island");
};
R_Date("S-1263", 5000, 80)
{
 Outlier("General", 0.05);
};
```

```
  R_Date("WIS-1706", 4080, 70)
  {
   Outlier("General", 0.05);
  };
  R_Date("Beta-247459", 4490, 40)
  {
   Outlier("Charcoal", 1);
  };
  Span("Old Copper");
  Interval("Old Copper");
 };
 Boundary("End Old Copper")
 {
  Start("Start of End Old Copper");
  Transition("Period of End Old Copper");
  End("End of End Old Copper");
 };
};
Sequence("Burnt Rollways")
{
 Boundary("Start Burnt Rollways")
 {
  Start("Start of Start Burnt Rollways");
  Transition("Period of Start Burnt Rollways");
  End("End of Start Burnt Rollways");
 };
 Phase("Burnt Rollways")
 {
  Sequence()
  {
   Boundary("Start Duck Lake");
   Phase("Duck Lake")
   {
    R_Date("Beta-099777", 3420, 50)
    {
     Outlier("Charcoal", 1);
    };
    R_Date("Beta-124454", 3400, 110)
    {
     Outlier("Charcoal", 1);
    };
   };
   Boundary("End Duck Lake");
  };
  R_Date("WIS-2269", 3630, 60)
  {
```

```
   Outlier("Charcoal", 1);
  };
  R_Date("WIS-2270", 3270, 80)
  {
   Outlier("Charcoal", 1);
  };
  R_Date("Beta-232440", 2280, 40)
  {
   Outlier("Charcoal", 1);
  };
  Span("Burnt Rollways");
  Interval("Burnt Rollways");
 };
 Boundary("End Burnt Rollways")
 {
  Start("Start of End Burnt Rollways");
  Transition("Period of End Burnt Rollways");
  End("End of End Burnt Rollways");
 };
};
Order()
{
};
};
```

Alternative Model A Code

```
Plot()
 {
  Sequence("Red Ochre")
  {
   Boundary("Start Red Ochre")
   {
    Start("Start of Start Red Ochre");
    Transition("Period of Start Red Ochre");
    End("End of Start Red Ochre");
   };
   Phase("Red Ochre")
   {
    R_Date("M-658", 3040, 150);
    R_Date("AA19679/WG2405", 2960, 50);
    R_Date("AA19685/WG2411", 2850, 50);
    R_Date("AA19680/WG2406", 2790, 50);
    R_Date("AA19677/WG2403", 2780, 65);
    R_Date("AA19684/WG2410", 2710, 50);
    R_Date("AA19686/WG2412", 2690, 60);
    R_Date("AA19682/WG2408", 2605, 45);
    R_Date("AA19683/WG2409", 2605, 50);
    R_Date("AA20282/WG2414", 2495, 65);
    R_Date("M-1719", 2460, 140);
    R_Date("AA19681/WG2407", 2380, 50);
    R_Date("M-1717", 2190, 140);
    R_Date("M-1718", 2080, 140);
    R_Date("M-1716", 2050, 130);
    R_Date("M-1715", 1949, 130);
    Interval("Red Ochre");
   };
   Boundary("End Red Ochre")
   {
    Start("Start of End Red Ochre");
    Transition("Period of End Red Ochre");
    End("End of End Red Ochre");
   };
  };
  Sequence("Old Copper")
  {
   Boundary("Start Old Copper")
   {
    Start("Start of Start Old Copper");
    Transition("Period of Start Old Copper");
    End("End of Start Old Copper");
```

```
};
Phase("Old Copper")
{
 Sequence()
 {
  Boundary("Start Oconto");
  Phase("Oconto")
  {
   R_Date("C-837/C-839", 7510, 600);
   R_Date("AA19678/WG2404", 6020, 60);
   R_Date("C-836", 5600, 600);
   R_Date("AA20281/WG2413", 5250, 110);
   R_Date("GAK", 4540, 400);
  };
  Boundary("End Oconto");
 };
 Sequence()
 {
  Boundary("Start Allumette Island");
  Phase("Allumette Island")
  {
   R_Date("Beta-141985", 5440, 80);
   R_Date("S-509", 5240, 80);
   R_Date("Beta-141986", 5270, 40);
   R_Date("Beta-141987", 4680, 40);
  };
  Boundary("End Allumette Island");
 };
 Sequence()
 {
  Boundary("Start Morrison Island");
  Phase("Morrison Island")
  {
   R_Date("Beta-88851", 4860, 50);
   R_Date("Beta-215300", 4820, 40);
   R_Date("Beta-215302", 4730, 40);
   R_Date("GSC-162", 4700, 150);
   R_Date("Beta-88852", 4630, 40);
   R_Date("Beta-88725", 4620, 40);
   R_Date("Beta-215301", 4210, 40);
  };
  Boundary("End Morrison Island");
 };
 Sequence()
 {
  Boundary("Start Osceola");
```

```
   Phase("Osceola")
   {
    R_Date("WIS-1706", 4080, 70);
    R_Date("M-643", 3450, 250);
   };
   Boundary("End Osceola");
  };
  Sequence()
  {
   Boundary("Start Reigh");
   Phase("Reigh")
   {
    R_Date("Beta-247459", 4490, 40);
    R_Date("M-644", 3660, 250);
   };
   Boundary("End Reigh");
  };
  R_Date("S-1263", 5000, 80);
  Interval("Old Copper");
 };
 Boundary("End Old Copper")
 {
  Start("Start of End Old Copper");
  Transition("Period of End Old Copper");
  End("End of End Old Copper");
 };
};
Sequence("Burnt Rollways")
{
 Boundary("Start Burnt Rollways")
 {
  Start("Start of Start Burnt Rollways");
  Transition("Period of Start Burnt Rollways");
  End("End of Start Burnt Rollways");
 };
 Phase("Burnt Rollways")
 {
  Sequence()
  {
   Boundary("Start Duck Lake");
   Phase("Duck Lake")
   {
    R_Date("Beta-099777", 3420, 50);
    R_Date("Beta-124454", 3400, 110);
   };
   Boundary("End Duck Lake");
```

```
   };
   R_Date("WIS-2269", 3630, 60);
   R_Date("WIS-2270", 3270, 80);
   R_Date("Beta-232440", 2280, 40);
   Interval("Burnt Rollways");
  };
  Boundary("End Burnt Rollways")
  {
   Start("Start of End Burnt Rollways");
   Transition("Period of End Burnt Rollways");
   End("End of End Burnt Rollways");
  };
 };
 Order()
 {
 };
};
```

Alternative Model B Code

```
Plot()
 {
  Outlier_Model("Charcoal",Exp(1,-10,0),U(0,3),"t");
  Outlier_Model("General",T(5),U(0,4),"t");
  Sequence("Red Ochre")
  {
   Boundary("Start Red Ochre")
   {
    Start("Start of Start Red Ochre");
    Transition("Period of Start Red Ochre");
    End("End of Start Red Ochre");
   };
   Phase("Red Ochre")
   {
    R_Date("M-658", 3040, 150)
    {
     Outlier("General", 0.05);
    };
    R_Date("AA19679/WG2405", 2960, 50)
    {
     Outlier("Charcoal", 1);
    };
    R_Date("AA19685/WG2411", 2850, 50)
    {
     Outlier("Charcoal", 1);
    };
    R_Date("AA19680/WG2406", 2790, 50)
    {
     Outlier("Charcoal", 1);
    };
    R_Date("AA19677/WG2403", 2780, 65)
    {
     Outlier("General", 0.05);
    };
    R_Date("AA19684/WG2410", 2710, 50)
    {
     Outlier("Charcoal", 1);
    };
    R_Date("AA19686/WG2412", 2690, 60)
    {
     Outlier("Charcoal", 1);
    };
    R_Date("AA19682/WG2408", 2605, 45)
    {
```

```
   Outlier("Charcoal", 1);
  };
  R_Date("AA19683/WG2409", 2605, 50)
  {
   Outlier("General", 0.05);
  };
  R_Date("AA20282/WG2414", 2495, 65)
  {
   Outlier("General", 0.05);
  };
  R_Date("M-1719", 2460, 140)
  {
   Outlier("Charcoal", 1);
  };
  R_Date("AA19681/WG2407", 2380, 50)
  {
   Outlier("Charcoal", 1);
  };
  R_Date("M-1717", 2190, 140)
  {
   Outlier("General", 0.05);
  };
  R_Date("M-1718", 2080, 140)
  {
   Outlier("Charcoal", 1);
  };
  R_Date("M-1716", 2050, 130)
  {
   Outlier("Charcoal", 1);
  };
  R_Date("M-1715", 1949, 130)
  {
   Outlier("Charcoal", 1);
  };
  Interval("Red Ochre");
 };
 Boundary("End Red Ochre")
 {
  Start("Start of End Red Ochre");
  Transition("Period of End Red Ochre");
  End("End of End Red Ochre");
 };
};
Sequence("Old Copper")
{
 Boundary("Start Old Copper")
```

```
{
 Start("Start of Start Old Copper");
 Transition("Period of Start Old Copper");
 End("End of Start Old Copper");
};
Phase("Old Copper")
{
 Sequence()
 {
  Boundary("Start Oconto");
  Phase("Oconto")
  {
   R_Date("C-837/C-839", 7510, 600)
   {
    Outlier("Charcoal", 1);
   };
   R_Date("AA19678/WG2404", 6020, 60)
   {
    Outlier("General", 0.05);
   };
   R_Date("C-836", 5600, 600)
   {
    Outlier("Charcoal", 1);
   };
   R_Date("AA20281/WG2413", 5250, 110)
   {
    Outlier("Charcoal", 1);
   };
   R_Date("GAK", 4540, 400)
   {
    Outlier("General", 0.05);
   };
  };
  Boundary("End Oconto");
 };
 Sequence()
 {
  Boundary("Start Allumette Island");
  Phase("Allumette Island")
  {
   R_Date("Beta-141985", 5440, 80)
   {
    Outlier("General", 0.05);
   };
   R_Date("S-509", 5240, 80)
   {
```

```
  Outlier("General", 0.05);
 };
 R_Date("Beta-141986", 5270, 40)
 {
  Outlier("General", 0.05);
 };
 R_Date("Beta-141987", 4680, 40)
 {
  Outlier("General", 0.05);
 };
};
Boundary("End Allumette Island");
};
Sequence()
{
Boundary("Start Morrison Island");
Phase("Morrison Island")
{
 R_Date("Beta-88851", 4860, 50)
 {
  Outlier("General", 0.05);
 };
 R_Date("Beta-215300", 4820, 40)
 {
  Outlier("General", 0.05);
 };
 R_Date("Beta-215302", 4730, 40)
 {
  Outlier("General", 0.05);
 };
 R_Date("GSC-162", 4700, 150)
 {
  Outlier("Charcoal", 1);
 };
 R_Date("Beta-88852", 4630, 40)
 {
  Outlier("General", 0.05);
 };
 R_Date("Beta-88725", 4620, 40)
 {
  Outlier("General", 0.05);
 };
 R_Date("Beta-215301", 4210, 40)
 {
  Outlier("General", 0.05);
 };
```

```
  };
  Boundary("End Morrison Island");
 };
 Sequence()
 {
  Boundary("Start Osceola");
  Phase("Osceola")
  {
   R_Date("WIS-1706", 4080, 70)
   {
    Outlier("General", 0.05);
   };
   R_Date("M-643", 3450, 250)
   {
    Outlier("General", 0.05);
   };
  };
  Boundary("End Osceola");
 };
 Sequence()
 {
  Boundary("Start Reigh");
  Phase("Reigh")
  {
   R_Date("Beta-247459", 4490, 40)
   {
    Outlier("Charcoal", 1);
   };
   R_Date("M-644", 3660, 250)
   {
    Outlier("General", 0.05);
   };
  };
  Boundary("End Reigh");
 };
 R_Date("S-1263", 5000, 80)
 {
  Outlier("General", 0.05);
 };
 Interval("Old Copper");
};
Boundary("End Old Copper")
{
 Start("Start of End Old Copper");
 Transition("Period of End Old Copper");
 End("End of End Old Copper");
```

```
  };
 };
 Sequence("Burnt Rollways")
 {
  Boundary("Start Burnt Rollways")
  {
   Start("Start of Start Burnt Rollways");
   Transition("Period of Start Burnt Rollways");
   End("End of Start Burnt Rollways");
  };
  Phase("Burnt Rollways")
  {
   Sequence()
   {
    Boundary("Start Duck Lake");
    Phase("Duck Lake")
    {
     R_Date("Beta-099777", 3420, 50)
     {
      Outlier("Charcoal", 1);
     };
     R_Date("Beta-124454", 3400, 110)
     {
      Outlier("Charcoal", 1);
     };
    };
    Boundary("End Duck Lake");
   };
   R_Date("WIS-2269", 3630, 60)
   {
    Outlier("Charcoal", 1);
   };
   R_Date("WIS-2270", 3270, 80)
   {
    Outlier("Charcoal", 1);
   };
   R_Date("Beta-232440", 2280, 40)
   {
    Outlier("Charcoal", 1);
   };
   Interval("Burnt Rollways");
  };
  Boundary("End Burnt Rollways")
  {
   Start("Start of End Burnt Rollways");
   Transition("Period of End Burnt Rollways");
```

```
  End("End of End Burnt Rollways");
 };
};
Order()
{
};
};
```

Alternative Model C Code

```
Plot()
 {
  Sequence("Red Ochre")
  {
   Boundary("Start Red Ochre")
   {
    Start("Start of Start Red Ochre");
    Transition("Period of Start Red Ochre");
    End("End of Start Red Ochre");
   };
   Phase("Red Ochre")
   {
    R_Date("M-658", 3040, 150);
    R_Date("AA19679/WG2405", 2960, 50);
    R_Date("AA19685/WG2411", 2850, 50);
    R_Date("AA19680/WG2406", 2790, 50);
    R_Date("AA19677/WG2403", 2780, 65);
    R_Date("AA19684/WG2410", 2710, 50);
    R_Date("AA19686/WG2412", 2690, 60);
    R_Date("AA19682/WG2408", 2605, 45);
    R_Date("AA19683/WG2409", 2605, 50);
    R_Date("AA20282/WG2414", 2495, 65);
    R_Date("M-1719", 2460, 140);
    R_Date("AA19681/WG2407", 2380, 50);
    R_Date("M-1717", 2190, 140);
    R_Date("M-1718", 2080, 140);
    R_Date("M-1716", 2050, 130);
    R_Date("M-1715", 1949, 130);
    Interval("Red Ochre");
   };
   Boundary("End Red Ochre")
   {
    Start("Start of End Red Ochre");
    Transition("Period of End Red Ochre");
    End("End of End Red Ochre");
   };
  };
  Sequence("Old Copper")
  {
   Boundary("Start Old Copper")
   {
    Start("Start of Start Old Copper");
    Transition("Period of Start Old Copper");
    End("End of Start Old Copper");
```

```
};
Phase("Old Copper")
{
 Sequence()
 {
  Boundary("Start Oconto");
  Phase("Oconto")
  {
   R_Date("AA19678/WG2404", 6020, 60);
   R_Date("AA20281/WG2413", 5250, 110);
  };
  Boundary("End Oconto");
 };
 Sequence()
 {
  Boundary("Start Allumette Island");
  Phase("Allumette Island")
  {
   R_Date("Beta-141985", 5440, 80);
   R_Date("S-509", 5240, 80);
   R_Date("Beta-141986", 5270, 40);
   R_Date("Beta-141987", 4680, 40);
  };
  Boundary("End Allumette Island");
 };
 Sequence()
 {
  Boundary("Start Morrison Island");
  Phase("Morrison Island")
  {
   R_Date("Beta-88851", 4860, 50);
   R_Date("Beta-215300", 4820, 40);
   R_Date("Beta-215302", 4730, 40);
   R_Date("GSC-162", 4700, 150);
   R_Date("Beta-88852", 4630, 40);
   R_Date("Beta-88725", 4620, 40);
   R_Date("Beta-215301", 4210, 40);
  };
  Boundary("End Morrison Island");
 };
 R_Date("S-1263", 5000, 80);
 R_Date("Beta-247459", 4490, 40);
 R_Date("WIS-1706", 4080, 70);
 Interval("Old Copper");
};
Boundary("End Old Copper")
```

```
  {
   Start("Start of End Old Copper");
   Transition("Period of End Old Copper");
   End("End of End Old Copper");
  };
 };
 Sequence("Burnt Rollways")
 {
  Boundary("Start Burnt Rollways")
  {
   Start("Start of Start Burnt Rollways");
   Transition("Period of Start Burnt Rollways");
   End("End of Start Burnt Rollways");
  };
  Phase("Burnt Rollways")
  {
   Sequence()
   {
    Boundary("Start Duck Lake");
    Phase("Duck Lake")
    {
     R_Date("Beta-099777", 3420, 50);
     R_Date("Beta-124454", 3400, 110);
    };
    Boundary("End Duck Lake");
   };
   R_Date("WIS-2269", 3630, 60);
   R_Date("WIS-2270", 3270, 80);
   R_Date("Beta-232440", 2280, 40);
   Interval("Burnt Rollways");
  };
  Boundary("End Burnt Rollways")
  {
   Start("Start of End Burnt Rollways");
   Transition("Period of End Burnt Rollways");
   End("End of End Burnt Rollways");
  };
 };
 Order()
 {
 };
};
```

Alternative Model D

```
Plot()
 {
  Outlier_Model("Charcoal",Exp(1,-10,0),U(0,3),"t");
  Outlier_Model("General",T(5),U(0,4),"t");
  Sequence("Red Ochre")
  {
   Sigma_Boundary("Start Red Ochre");
   Phase("Red Ochre")
   {
    R_Date("M-658", 3040, 150)
    {
     Outlier("General", 0.05);
    };
    R_Date("AA19679/WG2405", 2960, 50)
    {
     Outlier("Charcoal", 1);
    };
    R_Date("AA19685/WG2411", 2850, 50)
    {
     Outlier("Charcoal", 1);
    };
    R_Date("AA19680/WG2406", 2790, 50)
    {
     Outlier("Charcoal", 1);
    };
    R_Date("AA19677/WG2403", 2780, 65)
    {
     Outlier("General", 0.05);
    };
    R_Date("AA19684/WG2410", 2710, 50)
    {
     Outlier("Charcoal", 1);
    };
    R_Date("AA19686/WG2412", 2690, 60)
    {
     Outlier("Charcoal", 1);
    };
    R_Date("AA19682/WG2408", 2605, 45)
    {
     Outlier("Charcoal", 1);
    };
    R_Date("AA19683/WG2409", 2605, 50)
    {
     Outlier("General", 0.05);
```

```
  };
  R_Date("AA20282/WG2414", 2495, 65)
  {
   Outlier("General", 0.05);
  };
  R_Date("M-1719", 2460, 140)
  {
   Outlier("Charcoal", 1);
  };
  R_Date("AA19681/WG2407", 2380, 50)
  {
   Outlier("Charcoal", 1);
  };
  R_Date("M-1717", 2190, 140)
  {
   Outlier("General", 0.05);
  };
  R_Date("M-1718", 2080, 140)
  {
   Outlier("Charcoal", 1);
  };
  R_Date("M-1716", 2050, 130)
  {
   Outlier("Charcoal", 1);
  };
  R_Date("M-1715", 1949, 130)
  {
   Outlier("Charcoal", 1);
  };
  Span("Red Ochre");
  Interval("Red Ochre");
 };
 Sigma_Boundary("End Red Ochre");
};
Sequence("Old Copper")
{
 Sigma_Boundary("Start Old Copper");
 Phase("Old Copper")
 {
  Sequence()
  {
   Boundary("Start Oconto");
   Phase("Oconto")
   {
    R_Date("AA19678/WG2404", 6020, 60)
    {
```

```
   Outlier("General", 0.05);
  };
  R_Date("AA20281/WG2413", 5250, 110)
  {
   Outlier("Charcoal", 1);
  };
 };
 Boundary("End Oconto");
};
Sequence()
{
 Boundary("Start Allumette Island");
 Phase("Allumette Island")
 {
  R_Date("Beta-141985", 5440, 80)
  {
   Outlier("General", 0.05);
  };
  R_Date("S-509", 5240, 80)
  {
   Outlier("General", 0.05);
  };
  R_Date("Beta-141986", 5270, 40)
  {
   Outlier("General", 0.05);
  };
  R_Date("Beta-141987", 4680, 40)
  {
   Outlier("General", 0.05);
  };
 };
 Boundary("End Allumette Island");
};
Sequence()
{
 Boundary("Start Morrison Island");
 Phase("Morrison Island")
 {
  R_Date("Beta-88851", 4860, 50)
  {
   Outlier("General", 0.05);
  };
  R_Date("Beta-215300", 4820, 40)
  {
   Outlier("General", 0.05);
  };
```

```
    R_Date("Beta-215302", 4730, 40)
    {
     Outlier("General", 0.05);
    };
    R_Date("GSC-162", 4700, 150)
    {
     Outlier("Charcoal", 1);
    };
    R_Date("Beta-88852", 4630, 40)
    {
     Outlier("General", 0.05);
    };
    R_Date("Beta-88725", 4620, 40)
    {
     Outlier("General", 0.05);
    };
    R_Date("Beta-215301", 4210, 40)
    {
     Outlier("General", 0.05);
    };
   };
   Boundary("End Morrison Island");
  };
  R_Date("S-1263", 5000, 80)
  {
   Outlier("General", 0.05);
  };
  R_Date("WIS-1706", 4080, 70)
  {
   Outlier("General", 0.05);
  };
  R_Date("Beta-247459", 4490, 40)
  {
   Outlier("Charcoal", 1);
  };
  Span("Old Copper");
  Interval("Old Copper");
 };
 Sigma_Boundary("End Old Copper");
};
Sequence("Burnt Rollways")
{
 Sigma_Boundary("Start Burnt Rollways");
 Phase("Burnt Rollways")
 {
  Sequence()
```

```
   {
    Boundary("Start Duck Lake");
    Phase("Duck Lake")
    {
     R_Date("Beta-099777", 3420, 50)
     {
      Outlier("Charcoal", 1);
     };
     R_Date("Beta-124454", 3400, 110)
     {
      Outlier("Charcoal", 1);
     };
    };
    Boundary("End Duck Lake");
   };
   R_Date("WIS-2269", 3630, 60)
   {
    Outlier("Charcoal", 1);
   };
   R_Date("WIS-2270", 3270, 80)
   {
    Outlier("Charcoal", 1);
   };
   R_Date("Beta-232440", 2280, 40)
   {
    Outlier("Charcoal", 1);
   };
   Span("Burnt Rollways");
   Interval("Burnt Rollways");
  };
  Sigma_Boundary("End Burnt Rollways");
 };
 Order()
 {
 };
};
```

Burnt Rollways Variance: 10 Individual Runs of the Burnt Rollways Model

```
Plot()
 {
  Outlier_Model("Charcoal",Exp(1,-10,0),U(0,3),"t");
  Sequence("Burnt Rollways")
  {
   Boundary("Start Burnt Rollways")
   {
    Start("Start of Start Burnt Rollways");
    Transition("Period of Start Burnt Rollways");
    End("End of Start Burnt Rollways");
   };
   Phase("Burnt Rollways")
   {
    Sequence()
    {
     Boundary("Start Duck Lake");
     Phase("Duck Lake")
     {
      R_Date("Beta-099777", 3420, 50)
      {
       Outlier("Charcoal", 1);
      };
      R_Date("Beta-124454", 3400, 110)
      {
       Outlier("Charcoal", 1);
      };
     };
     Boundary("End Duck Lake");
    };
    R_Date("WIS-2269", 3630, 60)
    {
     Outlier("Charcoal", 1);
    };
    R_Date("WIS-2270", 3270, 80)
    {
     Outlier("Charcoal", 1);
    };
    R_Date("Beta-232440", 2280, 40)
    {
     Outlier("Charcoal", 1);
    };
    Span("Burnt Rollways");
    Interval("Burnt Rollways");
   };
```

```
  Boundary("End Burnt Rollways")
  {
   Start("Start of End Burnt Rollways");
   Transition("Period of End Burnt Rollways");
   End("End of End Burnt Rollways");
  };
 };
};
Plot()
{
 Outlier_Model("Charcoal",Exp(1,-10,0),U(0,3),"t");
 Sequence("Burnt Rollways")
 {
  Boundary("Start Burnt Rollways")
  {
   Start("Start of Start Burnt Rollways");
   Transition("Period of Start Burnt Rollways");
   End("End of Start Burnt Rollways");
  };
  Phase("Burnt Rollways")
  {
   Sequence()
   {
    Boundary("Start Duck Lake");
    Phase("Duck Lake")
    {
     R_Date("Beta-099777", 3420, 50)
     {
      Outlier("Charcoal", 1);
     };
     R_Date("Beta-124454", 3400, 110)
     {
      Outlier("Charcoal", 1);
     };
    };
    Boundary("End Duck Lake");
   };
   R_Date("WIS-2269", 3630, 60)
   {
    Outlier("Charcoal", 1);
   };
   R_Date("WIS-2270", 3270, 80)
   {
    Outlier("Charcoal", 1);
   };
   R_Date("Beta-232440", 2280, 40)
```

```
   {
    Outlier("Charcoal", 1);
   };
   Span("Burnt Rollways");
   Interval("Burnt Rollways");
  };
  Boundary("End Burnt Rollways")
  {
   Start("Start of End Burnt Rollways");
   Transition("Period of End Burnt Rollways");
   End("End of End Burnt Rollways");
  };
 };
};
Plot()
{
 Outlier_Model("Charcoal",Exp(1,-10,0),U(0,3),"t");
 Sequence("Burnt Rollways")
 {
  Boundary("Start Burnt Rollways")
  {
   Start("Start of Start Burnt Rollways");
   Transition("Period of Start Burnt Rollways");
   End("End of Start Burnt Rollways");
  };
  Phase("Burnt Rollways")
  {
   Sequence()
   {
    Boundary("Start Duck Lake");
    Phase("Duck Lake")
    {
     R_Date("Beta-099777", 3420, 50)
     {
      Outlier("Charcoal", 1);
     };
     R_Date("Beta-124454", 3400, 110)
     {
      Outlier("Charcoal", 1);
     };
    };
    Boundary("End Duck Lake");
   };
   R_Date("WIS-2269", 3630, 60)
   {
    Outlier("Charcoal", 1);
```

```
    };
    R_Date("WIS-2270", 3270, 80)
    {
     Outlier("Charcoal", 1);
    };
    R_Date("Beta-232440", 2280, 40)
    {
     Outlier("Charcoal", 1);
    };
    Span("Burnt Rollways");
    Interval("Burnt Rollways");
   };
   Boundary("End Burnt Rollways")
   {
    Start("Start of End Burnt Rollways");
    Transition("Period of End Burnt Rollways");
    End("End of End Burnt Rollways");
   };
  };
 };
Plot()
{
 Outlier_Model("Charcoal",Exp(1,-10,0),U(0,3),"t");
 Sequence("Burnt Rollways")
 {
  Boundary("Start Burnt Rollways")
  {
   Start("Start of Start Burnt Rollways");
   Transition("Period of Start Burnt Rollways");
   End("End of Start Burnt Rollways");
  };
  Phase("Burnt Rollways")
  {
   Sequence()
   {
    Boundary("Start Duck Lake");
    Phase("Duck Lake")
    {
     R_Date("Beta-099777", 3420, 50)
     {
      Outlier("Charcoal", 1);
     };
     R_Date("Beta-124454", 3400, 110)
     {
      Outlier("Charcoal", 1);
     };
```

```
    };
    Boundary("End Duck Lake");
   };
   R_Date("WIS-2269", 3630, 60)
   {
    Outlier("Charcoal", 1);
   };
   R_Date("WIS-2270", 3270, 80)
   {
    Outlier("Charcoal", 1);
   };
   R_Date("Beta-232440", 2280, 40)
   {
    Outlier("Charcoal", 1);
   };
   Span("Burnt Rollways");
   Interval("Burnt Rollways");
  };
  Boundary("End Burnt Rollways")
  {
   Start("Start of End Burnt Rollways");
   Transition("Period of End Burnt Rollways");
   End("End of End Burnt Rollways");
  };
 };
};
Plot()
{
 Outlier_Model("Charcoal",Exp(1,-10,0),U(0,3),"t");
 Sequence("Burnt Rollways")
 {
  Boundary("Start Burnt Rollways")
  {
   Start("Start of Start Burnt Rollways");
   Transition("Period of Start Burnt Rollways");
   End("End of Start Burnt Rollways");
  };
  Phase("Burnt Rollways")
  {
   Sequence()
   {
    Boundary("Start Duck Lake");
    Phase("Duck Lake")
    {
     R_Date("Beta-099777", 3420, 50)
     {
```

```
     Outlier("Charcoal", 1);
    };
    R_Date("Beta-124454", 3400, 110)
    {
     Outlier("Charcoal", 1);
    };
   };
   Boundary("End Duck Lake");
  };
  R_Date("WIS-2269", 3630, 60)
  {
   Outlier("Charcoal", 1);
  };
  R_Date("WIS-2270", 3270, 80)
  {
   Outlier("Charcoal", 1);
  };
  R_Date("Beta-232440", 2280, 40)
  {
   Outlier("Charcoal", 1);
  };
  Span("Burnt Rollways");
  Interval("Burnt Rollways");
 };
 Boundary("End Burnt Rollways")
 {
  Start("Start of End Burnt Rollways");
  Transition("Period of End Burnt Rollways");
  End("End of End Burnt Rollways");
 };
};
};
Plot()
{
 Outlier_Model("Charcoal",Exp(1,-10,0),U(0,3),"t");
 Sequence("Burnt Rollways")
 {
  Boundary("Start Burnt Rollways")
  {
   Start("Start of Start Burnt Rollways");
   Transition("Period of Start Burnt Rollways");
   End("End of Start Burnt Rollways");
  };
  Phase("Burnt Rollways")
  {
   Sequence()
```

```
   {
    Boundary("Start Duck Lake");
    Phase("Duck Lake")
    {
     R_Date("Beta-099777", 3420, 50)
     {
      Outlier("Charcoal", 1);
     };
     R_Date("Beta-124454", 3400, 110)
     {
      Outlier("Charcoal", 1);
     };
    };
    Boundary("End Duck Lake");
   };
   R_Date("WIS-2269", 3630, 60)
   {
    Outlier("Charcoal", 1);
   };
   R_Date("WIS-2270", 3270, 80)
   {
    Outlier("Charcoal", 1);
   };
   R_Date("Beta-232440", 2280, 40)
   {
    Outlier("Charcoal", 1);
   };
   Span("Burnt Rollways");
   Interval("Burnt Rollways");
  };
  Boundary("End Burnt Rollways")
  {
   Start("Start of End Burnt Rollways");
   Transition("Period of End Burnt Rollways");
   End("End of End Burnt Rollways");
  };
 };
};
Plot()
{
 Outlier_Model("Charcoal",Exp(1,-10,0),U(0,3),"t");
 Sequence("Burnt Rollways")
 {
  Boundary("Start Burnt Rollways")
  {
   Start("Start of Start Burnt Rollways");
```

```
  Transition("Period of Start Burnt Rollways");
  End("End of Start Burnt Rollways");
 };
 Phase("Burnt Rollways")
 {
  Sequence()
  {
   Boundary("Start Duck Lake");
   Phase("Duck Lake")
   {
    R_Date("Beta-099777", 3420, 50)
    {
     Outlier("Charcoal", 1);
    };
    R_Date("Beta-124454", 3400, 110)
    {
     Outlier("Charcoal", 1);
    };
   };
   Boundary("End Duck Lake");
  };
  R_Date("WIS-2269", 3630, 60)
  {
   Outlier("Charcoal", 1);
  };
  R_Date("WIS-2270", 3270, 80)
  {
   Outlier("Charcoal", 1);
  };
  R_Date("Beta-232440", 2280, 40)
  {
   Outlier("Charcoal", 1);
  };
  Span("Burnt Rollways");
  Interval("Burnt Rollways");
 };
 Boundary("End Burnt Rollways")
 {
  Start("Start of End Burnt Rollways");
  Transition("Period of End Burnt Rollways");
  End("End of End Burnt Rollways");
 };
};
};
Plot()
{
```

```
Outlier_Model("Charcoal",Exp(1,-10,0),U(0,3),"t");
Sequence("Burnt Rollways")
{
 Boundary("Start Burnt Rollways")
 {
  Start("Start of Start Burnt Rollways");
  Transition("Period of Start Burnt Rollways");
  End("End of Start Burnt Rollways");
 };
 Phase("Burnt Rollways")
 {
  Sequence()
  {
   Boundary("Start Duck Lake");
   Phase("Duck Lake")
   {
    R_Date("Beta-099777", 3420, 50)
    {
     Outlier("Charcoal", 1);
    };
    R_Date("Beta-124454", 3400, 110)
    {
     Outlier("Charcoal", 1);
    };
   };
   Boundary("End Duck Lake");
  };
  R_Date("WIS-2269", 3630, 60)
  {
   Outlier("Charcoal", 1);
  };
  R_Date("WIS-2270", 3270, 80)
  {
   Outlier("Charcoal", 1);
  };
  R_Date("Beta-232440", 2280, 40)
  {
   Outlier("Charcoal", 1);
  };
  Span("Burnt Rollways");
  Interval("Burnt Rollways");
 };
 Boundary("End Burnt Rollways")
 {
  Start("Start of End Burnt Rollways");
  Transition("Period of End Burnt Rollways");
```

```
   End("End of End Burnt Rollways");
  };
 };
};
Plot()
{
 Outlier_Model("Charcoal",Exp(1,-10,0),U(0,3),"t");
 Sequence("Burnt Rollways")
 {
  Boundary("Start Burnt Rollways")
  {
   Start("Start of Start Burnt Rollways");
   Transition("Period of Start Burnt Rollways");
   End("End of Start Burnt Rollways");
  };
  Phase("Burnt Rollways")
  {
   Sequence()
   {
    Boundary("Start Duck Lake");
    Phase("Duck Lake")
    {
     R_Date("Beta-099777", 3420, 50)
     {
      Outlier("Charcoal", 1);
     };
     R_Date("Beta-124454", 3400, 110)
     {
      Outlier("Charcoal", 1);
     };
    };
    Boundary("End Duck Lake");
   };
   R_Date("WIS-2269", 3630, 60)
   {
    Outlier("Charcoal", 1);
   };
   R_Date("WIS-2270", 3270, 80)
   {
    Outlier("Charcoal", 1);
   };
   R_Date("Beta-232440", 2280, 40)
   {
    Outlier("Charcoal", 1);
   };
   Span("Burnt Rollways");
```

```
   Interval("Burnt Rollways");
  };
  Boundary("End Burnt Rollways")
  {
   Start("Start of End Burnt Rollways");
   Transition("Period of End Burnt Rollways");
   End("End of End Burnt Rollways");
  };
 };
};
Plot()
{
 Outlier_Model("Charcoal",Exp(1,-10,0),U(0,3),"t");
 Sequence("Burnt Rollways")
 {
  Boundary("Start Burnt Rollways")
  {
   Start("Start of Start Burnt Rollways");
   Transition("Period of Start Burnt Rollways");
   End("End of Start Burnt Rollways");
  };
  Phase("Burnt Rollways")
  {
   Sequence()
   {
    Boundary("Start Duck Lake");
    Phase("Duck Lake")
    {
     R_Date("Beta-099777", 3420, 50)
     {
      Outlier("Charcoal", 1);
     };
     R_Date("Beta-124454", 3400, 110)
     {
      Outlier("Charcoal", 1);
     };
    };
    Boundary("End Duck Lake");
   };
   R_Date("WIS-2269", 3630, 60)
   {
    Outlier("Charcoal", 1);
   };
   R_Date("WIS-2270", 3270, 80)
   {
    Outlier("Charcoal", 1);
```

```
   };
   R_Date("Beta-232440", 2280, 40)
   {
    Outlier("Charcoal", 1);
   };
   Span("Burnt Rollways");
   Interval("Burnt Rollways");
  };
  Boundary("End Burnt Rollways")
  {
   Start("Start of End Burnt Rollways");
   Transition("Period of End Burnt Rollways");
   End("End of End Burnt Rollways");
  };
 };
};
```

Simulation Code: Plus 10 Simulated Dates for Each Tradition

```
Plot()
 {
  Outlier_Model("Charcoal",Exp(1,-10,0),U(0,3),"t");
  Outlier_Model("General",T(5),U(0,4),"t");
  Sequence("Red Ochre")
  {
   Boundary("Start Red Ochre")
   {
    Start("Start of Start Red Ochre");
    Transition("Period of Start Red Ochre");
    End("End of Start Red Ochre");
   };
   Phase("Red Ochre")
   {
    R_Date("M-658", 3040, 150)
    {
     Outlier("General", 0.05);
    };
    R_Date("AA19679/WG2405", 2960, 50)
    {
     Outlier("Charcoal", 1);
    };
    R_Date("AA19685/WG2411", 2850, 50)
    {
     Outlier("Charcoal", 1);
    };
    R_Date("AA19680/WG2406", 2790, 50)
    {
     Outlier("Charcoal", 1);
    };
    R_Date("AA19677/WG2403", 2780, 65)
    {
     Outlier("General", 0.05);
    };
    R_Date("AA19684/WG2410", 2710, 50)
    {
     Outlier("Charcoal", 1);
    };
    R_Date("AA19686/WG2412", 2690, 60)
    {
     Outlier("Charcoal", 1);
    };
    R_Date("AA19682/WG2408", 2605, 45)
    {
```

```
 Outlier("Charcoal", 1);
 };
 R_Date("AA19683/WG2409", 2605, 50)
 {
  Outlier("General", 0.05);
 };
 R_Date("AA20282/WG2414", 2495, 65)
 {
  Outlier("General", 0.05);
 };
 R_Date("M-1719", 2460, 140)
 {
  Outlier("Charcoal", 1);
 };
 R_Date("AA19681/WG2407", 2380, 50)
 {
  Outlier("Charcoal", 1);
 };
 R_Date("M-1717", 2190, 140)
 {
  Outlier("General", 0.05);
 };
 R_Date("M-1718", 2080, 140)
 {
  Outlier("Charcoal", 1);
 };
 R_Date("M-1716", 2050, 130)
 {
  Outlier("Charcoal", 1);
 };
 R_Date("M-1715", 1949, 130)
 {
  Outlier("Charcoal", 1);
 };
 R_Simulate("Sim_RO1", -755, 35);
 R_Simulate("Sim_RO2", -415, 35);
 R_Simulate("Sim_RO3", -777, 35);
 R_Simulate("Sim_RO4", -174, 35);
 R_Simulate("Sim_RO5", -428, 35);
 R_Simulate("Sim_RO6", -389, 35);
 R_Simulate("Sim_RO7", -985, 35);
 R_Simulate("Sim_RO8", -756, 35);
 R_Simulate("Sim_RO9", -401, 35);
 R_Simulate("Sim_RO10", -775, 35);
 Interval("Red Ochre");
};
```

```
  Boundary("End Red Ochre")
  {
   Start("Start of End Red Ochre");
   Transition("Period of End Red Ochre");
   End("End of End Red Ochre");
  };
 };
 Sequence("Old Copper")
 {
  Boundary("Start Old Copper")
  {
   Start("Start of Start Old Copper");
   Transition("Period of Start Old Copper");
   End("End of Start Old Copper");
  };
  Phase("Old Copper")
  {
   Sequence()
   {
    Boundary("Start Oconto");
    Phase("Oconto")
    {
     R_Date("AA19678/WG2404", 6020, 60)
     {
      Outlier("General", 0.05);
     };
     R_Date("AA20281/WG2413", 5250, 110)
     {
      Outlier("Charcoal", 1);
     };
    };
    Boundary("End Oconto");
   };
   Sequence()
   {
    Boundary("Start Allumette Island");
    Phase("Allumette Island")
    {
     R_Date("Beta-141985", 5440, 80)
     {
      Outlier("General", 0.05);
     };
     R_Date("S-509", 5240, 80)
     {
      Outlier("General", 0.05);
     };
```

```
  R_Date("Beta-141986", 5270, 40)
  {
   Outlier("General", 0.05);
  };
  R_Date("Beta-141987", 4680, 40)
  {
   Outlier("General", 0.05);
  };
 };
 Boundary("End Allumette Island");
};
Sequence()
{
 Boundary("Start Morrison Island");
 Phase("Morrison Island")
 {
  R_Date("Beta-88851", 4860, 50)
  {
   Outlier("General", 0.05);
  };
  R_Date("Beta-215300", 4820, 40)
  {
   Outlier("General", 0.05);
  };
  R_Date("Beta-215302", 4730, 40)
  {
   Outlier("General", 0.05);
  };
  R_Date("GSC-162", 4700, 150)
  {
   Outlier("Charcoal", 1);
  };
  R_Date("Beta-88852", 4630, 40)
  {
   Outlier("General", 0.05);
  };
  R_Date("Beta-88725", 4620, 40)
  {
   Outlier("General", 0.05);
  };
  R_Date("Beta-215301", 4210, 40)
  {
   Outlier("General", 0.05);
  };
 };
 Boundary("End Morrison Island");
```

```
  };
  R_Date("S-1263", 5000, 80)
  {
   Outlier("General", 0.05);
  };
  R_Date("WIS-1706", 4080, 70)
  {
   Outlier("General", 0.05);
  };
  R_Date("Beta-247459", 4490, 40)
  {
   Outlier("Charcoal", 1);
  };
  R_Simulate("Sim_OC1", -2574, 35);
  R_Simulate("Sim_OC2", -2826, 35);
  R_Simulate("Sim_OC3", -4664, 35);
  R_Simulate("Sim_OC4", -4896, 35);
  R_Simulate("Sim_OC5", -3635, 35);
  R_Simulate("Sim_OC6", -4366, 35);
  R_Simulate("Sim_OC7", -4686, 35);
  R_Simulate("Sim_OC8", -3350, 35);
  R_Simulate("Sim_OC9", -4366, 35);
  R_Simulate("Sim_OC10", -4001, 35);
  Interval("Old Copper");
 };
 Boundary("End Old Copper")
 {
  Start("Start of End Old Copper");
  Transition("Period of End Old Copper");
  End("End of End Old Copper");
 };
};
Sequence("Burnt Rollways")
{
 Boundary("Start Burnt Rollways")
 {
  Start("Start of Start Burnt Rollways");
  Transition("Period of Start Burnt Rollways");
  End("End of Start Burnt Rollways");
 };
 Phase("Burnt Rollways")
 {
  Sequence()
  {
   Boundary("Start Duck Lake");
   Phase("Duck Lake")
```

```
    {
     R_Date("Beta-099777", 3420, 50)
     {
      Outlier("Charcoal", 1);
     };
     R_Date("Beta-124454", 3400, 110)
     {
      Outlier("Charcoal", 1);
     };
    };
    Boundary("End Duck Lake");
   };
   R_Date("WIS-2269", 3630, 60)
   {
    Outlier("Charcoal", 1);
   };
   R_Date("WIS-2270", 3270, 80)
   {
    Outlier("Charcoal", 1);
   };
   R_Date("Beta-232440", 2280, 40)
   {
    Outlier("Charcoal", 1);
   };
   R_Simulate("Sim_BR1", -1658, 35);
   R_Simulate("Sim_BR2", -2062, 35);
   R_Simulate("Sim_BR3", -1630, 35);
   R_Simulate("Sim_BR4", -1099, 35);
   R_Simulate("Sim_BR5", -567, 35);
   R_Simulate("Sim_BR6", -1366, 35);
   R_Simulate("Sim_BR7", -1484, 35);
   R_Simulate("Sim_BR8", -2341, 35);
   R_Simulate("Sim_BR9", -1938, 35);
   R_Simulate("Sim_BR10", -1753, 35);
   Interval("Burnt Rollways");
  };
  Boundary("End Burnt Rollways")
  {
   Start("Start of End Burnt Rollways");
   Transition("Period of End Burnt Rollways");
   End("End of End Burnt Rollways");
  };
 };
 Order()
 {
 };
```

```
};
```

Simulation Code: Plus 20 Simulated Dates for Each Tradition

```
Plot()
 {
  Outlier_Model("Charcoal",Exp(1,-10,0),U(0,3),"t");
  Outlier_Model("General",T(5),U(0,4),"t");
  Sequence("Red Ochre")
  {
   Boundary("Start Red Ochre")
   {
    Start("Start of Start Red Ochre");
    Transition("Period of Start Red Ochre");
    End("End of Start Red Ochre");
   };
   Phase("Red Ochre")
   {
    R_Date("M-658", 3040, 150)
    {
     Outlier("General", 0.05);
    };
    R_Date("AA19679/WG2405", 2960, 50)
    {
     Outlier("Charcoal", 1);
    };
    R_Date("AA19685/WG2411", 2850, 50)
    {
     Outlier("Charcoal", 1);
    };
    R_Date("AA19680/WG2406", 2790, 50)
    {
     Outlier("Charcoal", 1);
    };
    R_Date("AA19677/WG2403", 2780, 65)
    {
     Outlier("General", 0.05);
    };
    R_Date("AA19684/WG2410", 2710, 50)
    {
     Outlier("Charcoal", 1);
    };
    R_Date("AA19686/WG2412", 2690, 60)
    {
     Outlier("Charcoal", 1);
    };
    R_Date("AA19682/WG2408", 2605, 45)
    {
```

```
 Outlier("Charcoal", 1);
};
R_Date("AA19683/WG2409", 2605, 50)
{
 Outlier("General", 0.05);
};
R_Date("AA20282/WG2414", 2495, 65)
{
 Outlier("General", 0.05);
};
R_Date("M-1719", 2460, 140)
{
 Outlier("Charcoal", 1);
};
R_Date("AA19681/WG2407", 2380, 50)
{
 Outlier("Charcoal", 1);
};
R_Date("M-1717", 2190, 140)
{
 Outlier("General", 0.05);
};
R_Date("M-1718", 2080, 140)
{
 Outlier("Charcoal", 1);
};
R_Date("M-1716", 2050, 130)
{
 Outlier("Charcoal", 1);
};
R_Date("M-1715", 1949, 130)
{
 Outlier("Charcoal", 1);
};
R_Simulate("Sim_RO1", -992, 35);
R_Simulate("Sim_RO2", -172, 35);
R_Simulate("Sim_RO3", -344, 35);
R_Simulate("Sim_RO4", -161, 35);
R_Simulate("Sim_RO5", -563, 35);
R_Simulate("Sim_RO6", -675, 35);
R_Simulate("Sim_RO7", -513, 35);
R_Simulate("Sim_RO8", -392, 35);
R_Simulate("Sim_RO9", -585, 35);
R_Simulate("Sim_RO10", -230, 35);
R_Simulate("Sim_RO11", -482, 35);
R_Simulate("Sim_RO12", -476, 35);
```

```
  R_Simulate("Sim_RO13", -123, 35);
  R_Simulate("Sim_RO14", -365, 35);
  R_Simulate("Sim_RO15", -494, 35);
  R_Simulate("Sim_RO16", -970, 35);
  R_Simulate("Sim_RO17", -490, 35);
  R_Simulate("Sim_RO18", -375, 35);
  R_Simulate("Sim_RO19", -416, 35);
  R_Simulate("Sim_RO20", -321, 35);
  Interval ("Red Ochre");
 };
 Boundary("End Red Ochre")
 {
  Start("Start of End Red Ochre");
  Transition("Period of End Red Ochre");
  End("End of End Red Ochre");
 };
};
Sequence("Old Copper")
{
 Boundary("Start Old Copper")
 {
  Start("Start of Start Old Copper");
  Transition("Period of Start Old Copper");
  End("End of Start Old Copper");
 };
 Phase("Old Copper")
 {
  Sequence()
  {
   Boundary("Start Oconto");
   Phase("Oconto")
   {
    R_Date("AA19678/WG2404", 6020, 60)
    {
     Outlier("General", 0.05);
    };
    R_Date("AA20281/WG2413", 5250, 110)
    {
     Outlier("Charcoal", 1);
    };
   };
   Boundary("End Oconto");
  };
  Sequence()
  {
   Boundary("Start Allumette Island");
```

```
 Phase("Allumette Island")
 {
  R_Date("Beta-141985", 5440, 80)
  {
   Outlier("General", 0.05);
  };
  R_Date("S-509", 5240, 80)
  {
   Outlier("General", 0.05);
  };
  R_Date("Beta-141986", 5270, 40)
  {
   Outlier("General", 0.05);
  };
  R_Date("Beta-141987", 4680, 40)
  {
   Outlier("General", 0.05);
  };
 };
 Boundary("End Allumette Island");
};
Sequence()
{
 Boundary("Start Morrison Island");
 Phase("Morrison Island")
 {
  R_Date("Beta-88851", 4860, 50)
  {
   Outlier("General", 0.05);
  };
  R_Date("Beta-215300", 4820, 40)
  {
   Outlier("General", 0.05);
  };
  R_Date("Beta-215302", 4730, 40)
  {
   Outlier("General", 0.05);
  };
  R_Date("GSC-162", 4700, 150)
  {
   Outlier("Charcoal", 1);
  };
  R_Date("Beta-88852", 4630, 40)
  {
   Outlier("General", 0.05);
  };
```

```
   R_Date("Beta-88725", 4620, 40)
   {
    Outlier("General", 0.05);
   };
   R_Date("Beta-215301", 4210, 40)
   {
    Outlier("General", 0.05);
   };
  };
  Boundary("End Morrison Island");
 };
 R_Date("S-1263", 5000, 80)
 {
  Outlier("General", 0.05);
 };
 R_Date("WIS-1706", 4080, 70)
 {
  Outlier("General", 0.05);
 };
 R_Date("Beta-247459", 4490, 40)
 {
  Outlier("Charcoal", 1);
 };
 R_Simulate("Sim_OC1", -4449, 35);
 R_Simulate("Sim_OC2", -2859, 35);
 R_Simulate("Sim_OC3", -5093, 35);
 R_Simulate("Sim_OC4", -4015, 35);
 R_Simulate("Sim_OC5", -2652, 35);
 R_Simulate("Sim_OC6", -2818, 35);
 R_Simulate("Sim_OC7", -4272, 35);
 R_Simulate("Sim_OC8", -3408, 35);
 R_Simulate("Sim_OC9", -5068, 35);
 R_Simulate("Sim_OC10", -4203, 35);
 R_Simulate("Sim_OC11", -3835, 35);
 R_Simulate("Sim_OC12", -5039, 35);
 R_Simulate("Sim_OC13", -4961, 35);
 R_Simulate("Sim_OC14", -3472, 35);
 R_Simulate("Sim_OC15", -3796, 35);
 R_Simulate("Sim_OC16", -3769, 35);
 R_Simulate("Sim_OC17", -2738, 35);
 R_Simulate("Sim_OC18", -3009, 35);
 R_Simulate("Sim_OC19", -5057, 35);
 R_Simulate("Sim_OC20", -3590, 35);
 Interval ("Old Copper");
};
Boundary("End Old Copper")
```

```
  {
   Start("Start of End Old Copper");
   Transition("Period of End Old Copper");
   End("End of End Old Copper");
  };
 };
 Sequence("Burnt Rollways")
 {
  Boundary("Start Burnt Rollways")
  {
   Start("Start of Start Burnt Rollways");
   Transition("Period of Start Burnt Rollways");
   End("End of Start Burnt Rollways");
  };
  Phase("Burnt Rollways")
  {
   Sequence()
   {
    Boundary("Start Duck Lake");
    Phase("Duck Lake")
    {
     R_Date("Beta-099777", 3420, 50)
     {
      Outlier("Charcoal", 1);
     };
     R_Date("Beta-124454", 3400, 110)
     {
      Outlier("Charcoal", 1);
     };
    };
    Boundary("End Duck Lake");
   };
   R_Date("WIS-2269", 3630, 60)
   {
    Outlier("Charcoal", 1);
   };
   R_Date("WIS-2270", 3270, 80)
   {
    Outlier("Charcoal", 1);
   };
   R_Date("Beta-232440", 2280, 40)
   {
    Outlier("Charcoal", 1);
   };
   R_Simulate("Sim_BR1", -273, 35);
   R_Simulate("Sim_BR2", -821, 35);
```

```
  R_Simulate("Sim_BR3", -647, 35);
  R_Simulate("Sim_BR4", -2419, 35);
  R_Simulate("Sim_BR5", -1093, 35);
  R_Simulate("Sim_BR6", -234, 35);
  R_Simulate("Sim_BR7", -2325, 35);
  R_Simulate("Sim_BR8", -1130, 35);
  R_Simulate("Sim_BR9", -2334, 35);
  R_Simulate("Sim_BR10", -304, 35);
  R_Simulate("Sim_BR11", -793, 35);
  R_Simulate("Sim_BR12", -2003, 35);
  R_Simulate("Sim_BR13", -291, 35);
  R_Simulate("Sim_BR14", -499, 35);
  R_Simulate("Sim_BR15", -1695, 35);
  R_Simulate("Sim_BR16", -1391, 35);
  R_Simulate("Sim_BR17", -259, 35);
  R_Simulate("Sim_BR18", -1029, 35);
  R_Simulate("Sim_BR19", -1831, 35);
  R_Simulate("Sim_BR20", -1056, 35);
  Interval ("Burnt Rollways");
 };
 Boundary("End Burnt Rollways")
 {
  Start("Start of End Burnt Rollways");
  Transition("Period of End Burnt Rollways");
  End("End of End Burnt Rollways");
 };
};
Order()
{
};
};
```

Simulation Code: Plus 30 Simulated Dates for Each Tradition

```
Plot()
 {
  Outlier_Model("Charcoal",Exp(1,-10,0),U(0,3),"t");
  Outlier_Model("General",T(5),U(0,4),"t");
  Sequence("Red Ochre")
  {
   Boundary("Start Red Ochre")
   {
    Start("Start of Start Red Ochre");
    Transition("Period of Start Red Ochre");
    End("End of Start Red Ochre");
   };
   Phase("Red Ochre")
   {
    R_Date("M-658", 3040, 150)
    {
     Outlier("General", 0.05);
    };
    R_Date("AA19679/WG2405", 2960, 50)
    {
     Outlier("Charcoal", 1);
    };
    R_Date("AA19685/WG2411", 2850, 50)
    {
     Outlier("Charcoal", 1);
    };
    R_Date("AA19680/WG2406", 2790, 50)
    {
     Outlier("Charcoal", 1);
    };
    R_Date("AA19677/WG2403", 2780, 65)
    {
     Outlier("General", 0.05);
    };
    R_Date("AA19684/WG2410", 2710, 50)
    {
     Outlier("Charcoal", 1);
    };
    R_Date("AA19686/WG2412", 2690, 60)
    {
     Outlier("Charcoal", 1);
    };
    R_Date("AA19682/WG2408", 2605, 45)
    {
```

```
 Outlier("Charcoal", 1);
};
R_Date("AA19683/WG2409", 2605, 50)
{
 Outlier("General", 0.05);
};
R_Date("AA20282/WG2414", 2495, 65)
{
 Outlier("General", 0.05);
};
R_Date("M-1719", 2460, 140)
{
 Outlier("Charcoal", 1);
};
R_Date("AA19681/WG2407", 2380, 50)
{
 Outlier("Charcoal", 1);
};
R_Date("M-1717", 2190, 140)
{
 Outlier("General", 0.05);
};
R_Date("M-1718", 2080, 140)
{
 Outlier("Charcoal", 1);
};
R_Date("M-1716", 2050, 130)
{
 Outlier("Charcoal", 1);
};
R_Date("M-1715", 1949, 130)
{
 Outlier("Charcoal", 1);
};
R_Simulate("Sim_RO1", -303, 35);
R_Simulate("Sim_RO2", -123, 35);
R_Simulate("Sim_RO3", -205, 35);
R_Simulate("Sim_RO4", -1061, 35);
R_Simulate("Sim_RO5", -765, 35);
R_Simulate("Sim_RO6", -533, 35);
R_Simulate("Sim_RO7", -344, 35);
R_Simulate("Sim_RO8", -630, 35);
R_Simulate("Sim_RO9", -962, 35);
R_Simulate("Sim_RO10", -246, 35);
R_Simulate("Sim_RO11", -920, 35);
R_Simulate("Sim_RO12", -619, 35);
```

```
  R_Simulate("Sim_RO13", -797, 35);
  R_Simulate("Sim_RO14", -674, 35);
  R_Simulate("Sim_RO15", -902, 35);
  R_Simulate("Sim_RO16", -248, 35);
  R_Simulate("Sim_RO17", -804, 35);
  R_Simulate("Sim_RO18", -627, 35);
  R_Simulate("Sim_RO19", -195, 35);
  R_Simulate("Sim_RO20", -1096, 35);
  R_Simulate("Sim_RO21", -540, 35);
  R_Simulate("Sim_RO22", -552, 35);
  R_Simulate("Sim_RO23", -530, 35);
  R_Simulate("Sim_RO24", -1100, 35);
  R_Simulate("Sim_RO25", -814, 35);
  R_Simulate("Sim_RO26", -184, 35);
  R_Simulate("Sim_RO27", -222, 35);
  R_Simulate("Sim_RO28", -596, 35);
  R_Simulate("Sim_RO29", -631, 35);
  R_Simulate("Sim_RO30", -454, 35);
  Interval ("Red Ochre");
 };
 Boundary("End Red Ochre")
 {
  Start("Start of End Red Ochre");
  Transition("Period of End Red Ochre");
  End("End of End Red Ochre");
 };
};
Sequence("Old Copper")
{
 Boundary("Start Old Copper")
 {
  Start("Start of Start Old Copper");
  Transition("Period of Start Old Copper");
  End("End of Start Old Copper");
 };
 Phase("Old Copper")
 {
  Sequence()
  {
   Boundary("Start Oconto");
   Phase("Oconto")
   {
    R_Date("AA19678/WG2404", 6020, 60)
    {
     Outlier("General", 0.05);
    };
```

```
  R_Date("AA20281/WG2413", 5250, 110)
  {
   Outlier("Charcoal", 1);
  };
 };
 Boundary("End Oconto");
};
Sequence()
{
 Boundary("Start Allumette Island");
 Phase("Allumette Island")
 {
  R_Date("Beta-141985", 5440, 80)
  {
   Outlier("General", 0.05);
  };
  R_Date("S-509", 5240, 80)
  {
   Outlier("General", 0.05);
  };
  R_Date("Beta-141986", 5270, 40)
  {
   Outlier("General", 0.05);
  };
  R_Date("Beta-141987", 4680, 40)
  {
   Outlier("General", 0.05);
  };
 };
 Boundary("End Allumette Island");
};
Sequence()
{
 Boundary("Start Morrison Island");
 Phase("Morrison Island")
 {
  R_Date("Beta-88851", 4860, 50)
  {
   Outlier("General", 0.05);
  };
  R_Date("Beta-215300", 4820, 40)
  {
   Outlier("General", 0.05);
  };
  R_Date("Beta-215302", 4730, 40)
  {
```

```
   Outlier("General", 0.05);
  };
  R_Date("GSC-162", 4700, 150)
  {
   Outlier("Charcoal", 1);
  };
  R_Date("Beta-88852", 4630, 40)
  {
   Outlier("General", 0.05);
  };
  R_Date("Beta-88725", 4620, 40)
  {
   Outlier("General", 0.05);
  };
  R_Date("Beta-215301", 4210, 40)
  {
   Outlier("General", 0.05);
  };
 };
 Boundary("End Morrison Island");
};
R_Date("S-1263", 5000, 80)
{
 Outlier("General", 0.05);
};
R_Date("WIS-1706", 4080, 70)
{
 Outlier("General", 0.05);
};
R_Date("Beta-247459", 4490, 40)
{
 Outlier("Charcoal", 1);
};
R_Simulate("Sim_OC1", -3173, 35);
R_Simulate("Sim_OC2", -3503, 35);
R_Simulate("Sim_OC3", -4586, 35);
R_Simulate("Sim_OC4", -4745, 35);
R_Simulate("Sim_OC5", -2569, 35);
R_Simulate("Sim_OC6", -3999, 35);
R_Simulate("Sim_OC7", -4577, 35);
R_Simulate("Sim_OC8", -4320, 35);
R_Simulate("Sim_OC9", -4962, 35);
R_Simulate("Sim_OC10", -4692, 35);
R_Simulate("Sim_OC11", -2516, 35);
R_Simulate("Sim_OC12", -4905, 35);
R_Simulate("Sim_OC13", -4982, 35);
```

```
  R_Simulate("Sim_OC14", -4947, 35);
  R_Simulate("Sim_OC15", -3238, 35);
  R_Simulate("Sim_OC16", -2630, 35);
  R_Simulate("Sim_OC17", -4091, 35);
  R_Simulate("Sim_OC18", -4345, 35);
  R_Simulate("Sim_OC19", -3411, 35);
  R_Simulate("Sim_OC20", -4184, 35);
  R_Simulate("Sim_OC21", -4909, 35);
  R_Simulate("Sim_OC22", -2862, 35);
  R_Simulate("Sim_OC23", -3756, 35);
  R_Simulate("Sim_OC24", -3199, 35);
  R_Simulate("Sim_OC25", -2534, 35);
  R_Simulate("Sim_OC26", -4835, 35);
  R_Simulate("Sim_OC27", -3887, 35);
  R_Simulate("Sim_OC28", -3346, 35);
  R_Simulate("Sim_OC29", -3536, 35);
  R_Simulate("Sim_OC30", -2726, 35);
  Interval ("Old Copper");
 };
 Boundary("End Old Copper")
 {
  Start("Start of End Old Copper");
  Transition("Period of End Old Copper");
  End("End of End Old Copper");
 };
};
Sequence("Burnt Rollways")
{
 Boundary("Start Burnt Rollways")
 {
  Start("Start of Start Burnt Rollways");
  Transition("Period of Start Burnt Rollways");
  End("End of Start Burnt Rollways");
 };
 Phase("Burnt Rollways")
 {
  Sequence()
  {
   Boundary("Start Duck Lake");
   Phase("Duck Lake")
   {
    R_Date("Beta-099777", 3420, 50)
    {
     Outlier("Charcoal", 1);
    };
    R_Date("Beta-124454", 3400, 110)
```

```
  {
   Outlier("Charcoal", 1);
  };
 };
 Boundary("End Duck Lake");
};
R_Date("WIS-2269", 3630, 60)
{
 Outlier("Charcoal", 1);
};
R_Date("WIS-2270", 3270, 80)
{
 Outlier("Charcoal", 1);
};
R_Date("Beta-232440", 2280, 40)
{
 Outlier("Charcoal", 1);
};
R_Simulate("Sim_BR1", -1809, 35);
R_Simulate("Sim_BR2", -1048, 35);
R_Simulate("Sim_BR3", -2376, 35);
R_Simulate("Sim_BR4", -912, 35);
R_Simulate("Sim_BR5", -567, 35);
R_Simulate("Sim_BR6", -1439, 35);
R_Simulate("Sim_BR7", -987, 35);
R_Simulate("Sim_BR8", -2088, 35);
R_Simulate("Sim_BR9", -1137, 35);
R_Simulate("Sim_BR10", -1154, 35);
R_Simulate("Sim_BR11", -289, 35);
R_Simulate("Sim_BR12", -936, 35);
R_Simulate("Sim_BR13", -2387, 35);
R_Simulate("Sim_BR14", -1366, 35);
R_Simulate("Sim_BR15", -379, 35);
R_Simulate("Sim_BR16", -904, 35);
R_Simulate("Sim_BR17", -529, 35);
R_Simulate("Sim_BR18", -1710, 35);
R_Simulate("Sim_BR19", -2252, 35);
R_Simulate("Sim_BR20", -1919, 35);
R_Simulate("Sim_BR21", -340, 35);
R_Simulate("Sim_BR22", -1776, 35);
R_Simulate("Sim_BR23", -931, 35);
R_Simulate("Sim_BR24", -429, 35);
R_Simulate("Sim_BR25", -2246, 35);
R_Simulate("Sim_BR26", -1934, 35);
R_Simulate("Sim_BR27", -573, 35);
R_Simulate("Sim_BR28", -1493, 35);
```

```
   R_Simulate("Sim_BR29", -105, 35);
   R_Simulate("Sim_BR30", -900, 35);
   Interval ("Burnt Rollways");
  };
  Boundary("End Burnt Rollways")
  {
   Start("Start of End Burnt Rollways");
   Transition("Period of End Burnt Rollways");
   End("End of End Burnt Rollways");
  };
 };
 Order()
 {
 };
};
```

Simulation Code: Plus 40 Simulated Dates for Each Tradition

```
Plot()
 {
  Outlier_Model("Charcoal",Exp(1,-10,0),U(0,3),"t");
  Outlier_Model("General",T(5),U(0,4),"t");
  Sequence("Red Ochre")
  {
   Boundary("Start Red Ochre")
   {
    Start("Start of Start Red Ochre");
    Transition("Period of Start Red Ochre");
    End("End of Start Red Ochre");
   };
   Phase("Red Ochre")
   {
    R_Date("M-658", 3040, 150)
    {
     Outlier("General", 0.05);
    };
    R_Date("AA19679/WG2405", 2960, 50)
    {
     Outlier("Charcoal", 1);
    };
    R_Date("AA19685/WG2411", 2850, 50)
    {
     Outlier("Charcoal", 1);
    };
    R_Date("AA19680/WG2406", 2790, 50)
    {
     Outlier("Charcoal", 1);
    };
    R_Date("AA19677/WG2403", 2780, 65)
    {
     Outlier("General", 0.05);
    };
    R_Date("AA19684/WG2410", 2710, 50)
    {
     Outlier("Charcoal", 1);
    };
    R_Date("AA19686/WG2412", 2690, 60)
    {
     Outlier("Charcoal", 1);
    };
    R_Date("AA19682/WG2408", 2605, 45)
    {
```

```
 Outlier("Charcoal", 1);
};
R_Date("AA19683/WG2409", 2605, 50)
{
 Outlier("General", 0.05);
};
R_Date("AA20282/WG2414", 2495, 65)
{
 Outlier("General", 0.05);
};
R_Date("M-1719", 2460, 140)
{
 Outlier("Charcoal", 1);
};
R_Date("AA19681/WG2407", 2380, 50)
{
 Outlier("Charcoal", 1);
};
R_Date("M-1717", 2190, 140)
{
 Outlier("General", 0.05);
};
R_Date("M-1718", 2080, 140)
{
 Outlier("Charcoal", 1);
};
R_Date("M-1716", 2050, 130)
{
 Outlier("Charcoal", 1);
};
R_Date("M-1715", 1949, 130)
{
 Outlier("Charcoal", 1);
};
R_Simulate("Sim_RO1", -710, 35);
R_Simulate("Sim_RO2", -795, 35);
R_Simulate("Sim_RO3", -1122, 35);
R_Simulate("Sim_RO4", -696, 35);
R_Simulate("Sim_RO5", -647, 35);
R_Simulate("Sim_RO6", -1102, 35);
R_Simulate("Sim_RO7", -823, 35);
R_Simulate("Sim_RO8", -982, 35);
R_Simulate("Sim_RO9", -509, 35);
R_Simulate("Sim_RO10", -1120, 35);
R_Simulate("Sim_RO11", -1141, 35);
R_Simulate("Sim_RO12", -645, 35);
```

```
  R_Simulate("Sim_RO13", -906, 35);
  R_Simulate("Sim_RO14", -859, 35);
  R_Simulate("Sim_RO15", -781, 35);
  R_Simulate("Sim_RO16", -667, 35);
  R_Simulate("Sim_RO17", -546, 35);
  R_Simulate("Sim_RO18", -145, 35);
  R_Simulate("Sim_RO19", -520, 35);
  R_Simulate("Sim_RO20", -193, 35);
  R_Simulate("Sim_RO21", -413, 35);
  R_Simulate("Sim_RO22", -376, 35);
  R_Simulate("Sim_RO23", -623, 35);
  R_Simulate("Sim_RO24", -976, 35);
  R_Simulate("Sim_RO25", -716, 35);
  R_Simulate("Sim_RO26", -934, 35);
  R_Simulate("Sim_RO27", -1006, 35);
  R_Simulate("Sim_RO28", -940, 35);
  R_Simulate("Sim_RO29", -388, 35);
  R_Simulate("Sim_RO30", -242, 35);
  R_Simulate("Sim_RO31", -403, 35);
  R_Simulate("Sim_RO32", -370, 35);
  R_Simulate("Sim_RO33", -504, 35);
  R_Simulate("Sim_RO34", -825, 35);
  R_Simulate("Sim_RO35", -736, 35);
  R_Simulate("Sim_RO36", -506, 35);
  R_Simulate("Sim_RO37", -332, 35);
  R_Simulate("Sim_RO38", -594, 35);
  R_Simulate("Sim_RO39", -295, 35);
  R_Simulate("Sim_RO40", -533, 35);
  Interval ("Red Ochre");
 };
 Boundary("End Red Ochre")
 {
  Start("Start of End Red Ochre");
  Transition("Period of End Red Ochre");
  End("End of End Red Ochre");
 };
};
Sequence("Old Copper")
{
 Boundary("Start Old Copper")
 {
  Start("Start of Start Old Copper");
  Transition("Period of Start Old Copper");
  End("End of Start Old Copper");
 };
 Phase("Old Copper")
```

```
{
 Sequence()
 {
  Boundary("Start Oconto");
  Phase("Oconto")
  {
   R_Date("AA19678/WG2404", 6020, 60)
   {
    Outlier("General", 0.05);
   };
   R_Date("AA20281/WG2413", 5250, 110)
   {
    Outlier("Charcoal", 1);
   };
  };
  Boundary("End Oconto");
 };
 Sequence()
 {
  Boundary("Start Allumette Island");
  Phase("Allumette Island")
  {
   R_Date("Beta-141985", 5440, 80)
   {
    Outlier("General", 0.05);
   };
   R_Date("S-509", 5240, 80)
   {
    Outlier("General", 0.05);
   };
   R_Date("Beta-141986", 5270, 40)
   {
    Outlier("General", 0.05);
   };
   R_Date("Beta-141987", 4680, 40)
   {
    Outlier("General", 0.05);
   };
  };
  Boundary("End Allumette Island");
 };
 Sequence()
 {
  Boundary("Start Morrison Island");
  Phase("Morrison Island")
  {
```

```
  R_Date("Beta-88851", 4860, 50)
  {
   Outlier("General", 0.05);
  };
  R_Date("Beta-215300", 4820, 40)
  {
   Outlier("General", 0.05);
  };
  R_Date("Beta-215302", 4730, 40)
  {
   Outlier("General", 0.05);
  };
  R_Date("GSC-162", 4700, 150)
  {
   Outlier("Charcoal", 1);
  };
  R_Date("Beta-88852", 4630, 40)
  {
   Outlier("General", 0.05);
  };
  R_Date("Beta-88725", 4620, 40)
  {
   Outlier("General", 0.05);
  };
  R_Date("Beta-215301", 4210, 40)
  {
   Outlier("General", 0.05);
  };
 };
 Boundary("End Morrison Island");
};
R_Date("S-1263", 5000, 80)
{
 Outlier("General", 0.05);
};
R_Date("WIS-1706", 4080, 70)
{
 Outlier("General", 0.05);
};
R_Date("Beta-247459", 4490, 40)
{
 Outlier("Charcoal", 1);
};
R_Simulate("Sim_OC1", -4179, 35);
R_Simulate("Sim_OC2", -3550, 35);
R_Simulate("Sim_OC3", -3519, 35);
```

```
 R_Simulate("Sim_OC4", -4641, 35);
 R_Simulate("Sim_OC5", -3383, 35);
 R_Simulate("Sim_OC6", -3946, 35);
 R_Simulate("Sim_OC7", -4443, 35);
 R_Simulate("Sim_OC8", -2805, 35);
 R_Simulate("Sim_OC9", -4959, 35);
 R_Simulate("Sim_OC10", -4811, 35);
 R_Simulate("Sim_OC11", -2698, 35);
 R_Simulate("Sim_OC12", -3596, 35);
 R_Simulate("Sim_OC13", -2843, 35);
 R_Simulate("Sim_OC14", -2676, 35);
 R_Simulate("Sim_OC15", -4103, 35);
 R_Simulate("Sim_OC16", -4798, 35);
 R_Simulate("Sim_OC17", -4528, 35);
 R_Simulate("Sim_OC18", -3074, 35);
 R_Simulate("Sim_OC19", -2652, 35);
 R_Simulate("Sim_OC20", -3204, 35);
 R_Simulate("Sim_OC21", -4419, 35);
 R_Simulate("Sim_OC22", -3361, 35);
 R_Simulate("Sim_OC23", -2987, 35);
 R_Simulate("Sim_OC24", -3981, 35);
 R_Simulate("Sim_OC25", -2566, 35);
 R_Simulate("Sim_OC26", -2880, 35);
 R_Simulate("Sim_OC27", -3489, 35);
 R_Simulate("Sim_OC28", -3151, 35);
 R_Simulate("Sim_OC29", -4734, 35);
 R_Simulate("Sim_OC30", -4815, 35);
 R_Simulate("Sim_OC31", -3273, 35);
 R_Simulate("Sim_OC32", -3509, 35);
 R_Simulate("Sim_OC33", -2789, 35);
 R_Simulate("Sim_OC34", -4373, 35);
 R_Simulate("Sim_OC35", -2595, 35);
 R_Simulate("Sim_OC36", -2658, 35);
 R_Simulate("Sim_OC37", -2969, 35);
 R_Simulate("Sim_OC38", -3513, 35);
 R_Simulate("Sim_OC39", -5046, 35);
 R_Simulate("Sim_OC40", -4396, 35);
 Interval ("Old Copper");
};
Boundary("End Old Copper")
{
 Start("Start of End Old Copper");
 Transition("Period of End Old Copper");
 End("End of End Old Copper");
};
};
```

```
Sequence("Burnt Rollways")
{
 Boundary("Start Burnt Rollways")
 {
  Start("Start of Start Burnt Rollways");
  Transition("Period of Start Burnt Rollways");
  End("End of Start Burnt Rollways");
 };
 Phase("Burnt Rollways")
 {
  Sequence()
  {
   Boundary("Start Duck Lake");
   Phase("Duck Lake")
   {
    R_Date("Beta-099777", 3420, 50)
    {
     Outlier("Charcoal", 1);
    };
    R_Date("Beta-124454", 3400, 110)
    {
     Outlier("Charcoal", 1);
    };
   };
   Boundary("End Duck Lake");
  };
  R_Date("WIS-2269", 3630, 60)
  {
   Outlier("Charcoal", 1);
  };
  R_Date("WIS-2270", 3270, 80)
  {
   Outlier("Charcoal", 1);
  };
  R_Date("Beta-232440", 2280, 40)
  {
   Outlier("Charcoal", 1);
  };
  R_Simulate("Sim_BR1", -2446, 35);
  R_Simulate("Sim_BR2", -1399, 35);
  R_Simulate("Sim_BR3", -2168, 35);
  R_Simulate("Sim_BR4", -703, 35);
  R_Simulate("Sim_BR5", -1755, 35);
  R_Simulate("Sim_BR6", -1263, 35);
  R_Simulate("Sim_BR7", -2362, 35);
  R_Simulate("Sim_BR8", -845, 35);
```

```
  R_Simulate("Sim_BR9", -2158, 35);
  R_Simulate("Sim_BR10", -1971, 35);
  R_Simulate("Sim_BR11", -158, 35);
  R_Simulate("Sim_BR12", -446, 35);
  R_Simulate("Sim_BR13", -624, 35);
  R_Simulate("Sim_BR14", -760, 35);
  R_Simulate("Sim_BR15", -940, 35);
  R_Simulate("Sim_BR16", -2426, 35);
  R_Simulate("Sim_BR17", -808, 35);
  R_Simulate("Sim_BR18", -1189, 35);
  R_Simulate("Sim_BR19", -57, 35);
  R_Simulate("Sim_BR20", -2372, 35);
  R_Simulate("Sim_BR21", -450, 35);
  R_Simulate("Sim_BR22", -969, 35);
  R_Simulate("Sim_BR23", -2108, 35);
  R_Simulate("Sim_BR24", -1364, 35);
  R_Simulate("Sim_BR25", -172, 35);
  R_Simulate("Sim_BR26", -413, 35);
  R_Simulate("Sim_BR27", -1087, 35);
  R_Simulate("Sim_BR28", -1580, 35);
  R_Simulate("Sim_BR29", -375, 35);
  R_Simulate("Sim_BR30", -1887, 35);
  R_Simulate("Sim_BR31", -1055, 35);
  R_Simulate("Sim_BR32", -2365, 35);
  R_Simulate("Sim_BR33", -1663, 35);
  R_Simulate("Sim_BR34", -1816, 35);
  R_Simulate("Sim_BR35", -2259, 35);
  R_Simulate("Sim_BR36", -1145, 35);
  R_Simulate("Sim_BR37", -1136, 35);
  R_Simulate("Sim_BR38", -646, 35);
  R_Simulate("Sim_BR39", -386, 35);
  R_Simulate("Sim_BR40", -1606, 35);
  Interval ("Burnt Rollways");
 };
 Boundary("End Burnt Rollways")
 {
  Start("Start of End Burnt Rollways");
  Transition("Period of End Burnt Rollways");
  End("End of End Burnt Rollways");
 };
};
Order()
{
};
};
```

Simulation Code: Plus 50 Simulated Dates for Each Tradition

```
Plot()
 {
  Outlier_Model("Charcoal",Exp(1,-10,0),U(0,3),"t");
  Outlier_Model("General",T(5),U(0,4),"t");
  Sequence("Red Ochre")
  {
   Boundary("Start Red Ochre")
   {
    Start("Start of Start Red Ochre");
    Transition("Period of Start Red Ochre");
    End("End of Start Red Ochre");
   };
   Phase("Red Ochre")
   {
    R_Date("M-658", 3040, 150)
    {
     Outlier("General", 0.05);
    };
    R_Date("AA19679/WG2405", 2960, 50)
    {
     Outlier("Charcoal", 1);
    };
    R_Date("AA19685/WG2411", 2850, 50)
    {
     Outlier("Charcoal", 1);
    };
    R_Date("AA19680/WG2406", 2790, 50)
    {
     Outlier("Charcoal", 1);
    };
    R_Date("AA19677/WG2403", 2780, 65)
    {
     Outlier("General", 0.05);
    };
    R_Date("AA19684/WG2410", 2710, 50)
    {
     Outlier("Charcoal", 1);
    };
    R_Date("AA19686/WG2412", 2690, 60)
    {
     Outlier("Charcoal", 1);
    };
    R_Date("AA19682/WG2408", 2605, 45)
    {
```

```
 Outlier("Charcoal", 1);
};
R_Date("AA19683/WG2409", 2605, 50)
{
 Outlier("General", 0.05);
};
R_Date("AA20282/WG2414", 2495, 65)
{
 Outlier("General", 0.05);
};
R_Date("M-1719", 2460, 140)
{
 Outlier("Charcoal", 1);
};
R_Date("AA19681/WG2407", 2380, 50)
{
 Outlier("Charcoal", 1);
};
R_Date("M-1717", 2190, 140)
{
 Outlier("General", 0.05);
};
R_Date("M-1718", 2080, 140)
{
 Outlier("Charcoal", 1);
};
R_Date("M-1716", 2050, 130)
{
 Outlier("Charcoal", 1);
};
R_Date("M-1715", 1949, 130)
{
 Outlier("Charcoal", 1);
};
R_Simulate("Sim_RO1", -993, 35);
R_Simulate("Sim_RO2", -761, 35);
R_Simulate("Sim_RO3", -226, 35);
R_Simulate("Sim_RO4", -720, 35);
R_Simulate("Sim_RO5", -538, 35);
R_Simulate("Sim_RO6", -310, 35);
R_Simulate("Sim_RO7", -558, 35);
R_Simulate("Sim_RO8", -776, 35);
R_Simulate("Sim_RO9", -1168, 35);
R_Simulate("Sim_RO10", -256, 35);
R_Simulate("Sim_RO11", -1140, 35);
R_Simulate("Sim_RO12", -772, 35);
```

```
 R_Simulate("Sim_RO13", -350, 35);
 R_Simulate("Sim_RO14", -801, 35);
 R_Simulate("Sim_RO15", -930, 35);
 R_Simulate("Sim_RO16", -1063, 35);
 R_Simulate("Sim_RO17", -1064, 35);
 R_Simulate("Sim_RO18", -166, 35);
 R_Simulate("Sim_RO19", -938, 35);
 R_Simulate("Sim_RO20", -673, 35);
 R_Simulate("Sim_RO21", -1032, 35);
 R_Simulate("Sim_RO22", -683, 35);
 R_Simulate("Sim_RO23", -959, 35);
 R_Simulate("Sim_RO24", -1027, 35);
 R_Simulate("Sim_RO25", -177, 35);
 R_Simulate("Sim_RO26", -538, 35);
 R_Simulate("Sim_RO27", -521, 35);
 R_Simulate("Sim_RO28", -1152, 35);
 R_Simulate("Sim_RO29", -690, 35);
 R_Simulate("Sim_RO30", -849, 35);
 R_Simulate("Sim_RO31", -294, 35);
 R_Simulate("Sim_RO32", -585, 35);
 R_Simulate("Sim_RO33", -1000, 35);
 R_Simulate("Sim_RO34", -271, 35);
 R_Simulate("Sim_RO35", -787, 35);
 R_Simulate("Sim_RO36", -612, 35);
 R_Simulate("Sim_RO37", -564, 35);
 R_Simulate("Sim_RO38", -311, 35);
 R_Simulate("Sim_RO39", -952, 35);
 R_Simulate("Sim_RO40", -674, 35);
 R_Simulate("Sim_RO41", -802, 35);
 R_Simulate("Sim_RO42", -635, 35);
 R_Simulate("Sim_RO43", -952, 35);
 R_Simulate("Sim_RO44", -671, 35);
 R_Simulate("Sim_RO45", -552, 35);
 R_Simulate("Sim_RO46", -347, 35);
 R_Simulate("Sim_RO47", -554, 35);
 R_Simulate("Sim_RO48", -590, 35);
 R_Simulate("Sim_RO49", -1164, 35);
 R_Simulate("Sim_RO50", -840, 35);
 Interval ("Red Ochre");
};
Boundary("End Red Ochre")
{
 Start("Start of End Red Ochre");
 Transition("Period of End Red Ochre");
 End("End of End Red Ochre");
};
```

```
};
Sequence("Old Copper")
{
 Boundary("Start Old Copper")
 {
  Start("Start of Start Old Copper");
  Transition("Period of Start Old Copper");
  End("End of Start Old Copper");
 };
 Phase("Old Copper")
 {
  Sequence()
  {
   Boundary("Start Oconto");
   Phase("Oconto")
   {
    R_Date("AA19678/WG2404", 6020, 60)
    {
     Outlier("General", 0.05);
    };
    R_Date("AA20281/WG2413", 5250, 110)
    {
     Outlier("Charcoal", 1);
    };
   };
   Boundary("End Oconto");
  };
  Sequence()
  {
   Boundary("Start Allumette Island");
   Phase("Allumette Island")
   {
    R_Date("Beta-141985", 5440, 80)
    {
     Outlier("General", 0.05);
    };
    R_Date("S-509", 5240, 80)
    {
     Outlier("General", 0.05);
    };
    R_Date("Beta-141986", 5270, 40)
    {
     Outlier("General", 0.05);
    };
    R_Date("Beta-141987", 4680, 40)
    {
```

```
   Outlier("General", 0.05);
  };
 };
 Boundary("End Allumette Island");
};
Sequence()
{
 Boundary("Start Morrison Island");
 Phase("Morrison Island")
 {
  R_Date("Beta-88851", 4860, 50)
  {
   Outlier("General", 0.05);
  };
  R_Date("Beta-215300", 4820, 40)
  {
   Outlier("General", 0.05);
  };
  R_Date("Beta-215302", 4730, 40)
  {
   Outlier("General", 0.05);
  };
  R_Date("GSC-162", 4700, 150)
  {
   Outlier("Charcoal", 1);
  };
  R_Date("Beta-88852", 4630, 40)
  {
   Outlier("General", 0.05);
  };
  R_Date("Beta-88725", 4620, 40)
  {
   Outlier("General", 0.05);
  };
  R_Date("Beta-215301", 4210, 40)
  {
   Outlier("General", 0.05);
  };
 };
 Boundary("End Morrison Island");
};
R_Date("S-1263", 5000, 80)
{
 Outlier("General", 0.05);
};
R_Date("WIS-1706", 4080, 70)
```

```
{
 Outlier("General", 0.05);
};
R_Date("Beta-247459", 4490, 40)
{
 Outlier("Charcoal", 1);
};
R_Simulate("Sim_OC1", -3158, 35);
R_Simulate("Sim_OC2", -3049, 35);
R_Simulate("Sim_OC3", -4441, 35);
R_Simulate("Sim_OC4", -2659, 35);
R_Simulate("Sim_OC5", -3795, 35);
R_Simulate("Sim_OC6", -4283, 35);
R_Simulate("Sim_OC7", -3233, 35);
R_Simulate("Sim_OC8", -4974, 35);
R_Simulate("Sim_OC9", -3409, 35);
R_Simulate("Sim_OC10", -4321, 35);
R_Simulate("Sim_OC11", -3472, 35);
R_Simulate("Sim_OC12", -4159, 35);
R_Simulate("Sim_OC13", -3458, 35);
R_Simulate("Sim_OC14", -4741, 35);
R_Simulate("Sim_OC15", -3328, 35);
R_Simulate("Sim_OC16", -2971, 35);
R_Simulate("Sim_OC17", -3660, 35);
R_Simulate("Sim_OC18", -3428, 35);
R_Simulate("Sim_OC19", -2641, 35);
R_Simulate("Sim_OC20", -2740, 35);
R_Simulate("Sim_OC21", -4022, 35);
R_Simulate("Sim_OC22", -4517, 35);
R_Simulate("Sim_OC23", -2765, 35);
R_Simulate("Sim_OC24", -4788, 35);
R_Simulate("Sim_OC25", -4103, 35);
R_Simulate("Sim_OC26", -3145, 35);
R_Simulate("Sim_OC27", -5024, 35);
R_Simulate("Sim_OC28", -5121, 35);
R_Simulate("Sim_OC29", -3427, 35);
R_Simulate("Sim_OC30", -2646, 35);
R_Simulate("Sim_OC31", -2605, 35);
R_Simulate("Sim_OC32", -4101, 35);
R_Simulate("Sim_OC33", -3825, 35);
R_Simulate("Sim_OC34", -2613, 35);
R_Simulate("Sim_OC35", -3106, 35);
R_Simulate("Sim_OC36", -3704, 35);
R_Simulate("Sim_OC37", -4794, 35);
R_Simulate("Sim_OC38", -3096, 35);
R_Simulate("Sim_OC39", -4040, 35);
```

```
  R_Simulate("Sim_OC40", -4246, 35);
  R_Simulate("Sim_OC41", -4161, 35);
  R_Simulate("Sim_OC42", -2988, 35);
  R_Simulate("Sim_OC43", -2752, 35);
  R_Simulate("Sim_OC44", -3049, 35);
  R_Simulate("Sim_OC45", -4408, 35);
  R_Simulate("Sim_OC46", -4094, 35);
  R_Simulate("Sim_OC47", -3889, 35);
  R_Simulate("Sim_OC48", -3411, 35);
  R_Simulate("Sim_OC49", -2927, 35);
  R_Simulate("Sim_OC50", -4875, 35);
  Interval ("Old Copper");
 };
 Boundary("End Old Copper")
 {
  Start("Start of End Old Copper");
  Transition("Period of End Old Copper");
  End("End of End Old Copper");
 };
};
Sequence("Burnt Rollways")
{
 Boundary("Start Burnt Rollways")
 {
  Start("Start of Start Burnt Rollways");
  Transition("Period of Start Burnt Rollways");
  End("End of Start Burnt Rollways");
 };
 Phase("Burnt Rollways")
 {
  Sequence()
  {
   Boundary("Start Duck Lake");
   Phase("Duck Lake")
   {
    R_Date("Beta-099777", 3420, 50)
    {
     Outlier("Charcoal", 1);
    };
    R_Date("Beta-124454", 3400, 110)
    {
     Outlier("Charcoal", 1);
    };
   };
   Boundary("End Duck Lake");
  };
```

```
R_Date("WIS-2269", 3630, 60)
{
 Outlier("Charcoal", 1);
};
R_Date("WIS-2270", 3270, 80)
{
 Outlier("Charcoal", 1);
};
R_Date("Beta-232440", 2280, 40)
{
 Outlier("Charcoal", 1);
};
R_Simulate("Sim_BR1", -757, 35);
R_Simulate("Sim_BR2", -1938, 35);
R_Simulate("Sim_BR3", -1214, 35);
R_Simulate("Sim_BR4", -1249, 35);
R_Simulate("Sim_BR5", -1962, 35);
R_Simulate("Sim_BR6", -1972, 35);
R_Simulate("Sim_BR7", -2092, 35);
R_Simulate("Sim_BR8", -2034, 35);
R_Simulate("Sim_BR9", -731, 35);
R_Simulate("Sim_BR10", -1732, 35);
R_Simulate("Sim_BR11", -205, 35);
R_Simulate("Sim_BR12", -156, 35);
R_Simulate("Sim_BR13", -714, 35);
R_Simulate("Sim_BR14", -1736, 35);
R_Simulate("Sim_BR15", -649, 35);
R_Simulate("Sim_BR16", -578, 35);
R_Simulate("Sim_BR17", -1331, 35);
R_Simulate("Sim_BR18", -1537, 35);
R_Simulate("Sim_BR19", -762, 35);
R_Simulate("Sim_BR20", -1050, 35);
R_Simulate("Sim_BR21", -2394, 35);
R_Simulate("Sim_BR22", -1000, 35);
R_Simulate("Sim_BR23", -1457, 35);
R_Simulate("Sim_BR24", -204, 35);
R_Simulate("Sim_BR25", -1241, 35);
R_Simulate("Sim_BR26", -2394, 35);
R_Simulate("Sim_BR27", -1479, 35);
R_Simulate("Sim_BR28", -1804, 35);
R_Simulate("Sim_BR29", -1350, 35);
R_Simulate("Sim_BR30", -1417, 35);
R_Simulate("Sim_BR31", -2452, 35);
R_Simulate("Sim_BR32", -1177, 35);
R_Simulate("Sim_BR33", -1393, 35);
R_Simulate("Sim_BR34", -949, 35);
```

```
  R_Simulate("Sim_BR35", -955, 35);
  R_Simulate("Sim_BR36", -1969, 35);
  R_Simulate("Sim_BR37", -1956, 35);
  R_Simulate("Sim_BR38", -633, 35);
  R_Simulate("Sim_BR39", -2063, 35);
  R_Simulate("Sim_BR40", -1960, 35);
  R_Simulate("Sim_BR41", -285, 35);
  R_Simulate("Sim_BR42", -638, 35);
  R_Simulate("Sim_BR43", -1397, 35);
  R_Simulate("Sim_BR44", -1492, 35);
  R_Simulate("Sim_BR45", -335, 35);
  R_Simulate("Sim_BR46", -2345, 35);
  R_Simulate("Sim_BR47", -150, 35);
  R_Simulate("Sim_BR48", -2054, 35);
  R_Simulate("Sim_BR49", -1162, 35);
  R_Simulate("Sim_BR50", -1556, 35);
  Interval ("Burnt Rollways");
 };
 Boundary("End Burnt Rollways")
 {
  Start("Start of End Burnt Rollways");
  Transition("Period of End Burnt Rollways");
  End("End of End Burnt Rollways");
 };
};
Order()
{
};
};
```

Simulation Code: Plus 60 Simulated Dates for Each Tradition

```
Plot()
 {
  Outlier_Model("Charcoal",Exp(1,-10,0),U(0,3),"t");
  Outlier_Model("General",T(5),U(0,4),"t");
  Sequence("Red Ochre")
  {
   Boundary("Start Red Ochre")
   {
    Start("Start of Start Red Ochre");
    Transition("Period of Start Red Ochre");
    End("End of Start Red Ochre");
   };
   Phase("Red Ochre")
   {
    R_Date("M-658", 3040, 150)
    {
     Outlier("General", 0.05);
    };
    R_Date("AA19679/WG2405", 2960, 50)
    {
     Outlier("Charcoal", 1);
    };
    R_Date("AA19685/WG2411", 2850, 50)
    {
     Outlier("Charcoal", 1);
    };
    R_Date("AA19680/WG2406", 2790, 50)
    {
     Outlier("Charcoal", 1);
    };
    R_Date("AA19677/WG2403", 2780, 65)
    {
     Outlier("General", 0.05);
    };
    R_Date("AA19684/WG2410", 2710, 50)
    {
     Outlier("Charcoal", 1);
    };
    R_Date("AA19686/WG2412", 2690, 60)
    {
     Outlier("Charcoal", 1);
    };
    R_Date("AA19682/WG2408", 2605, 45)
    {
```

```
 Outlier("Charcoal", 1);
};
R_Date("AA19683/WG2409", 2605, 50)
{
 Outlier("General", 0.05);
};
R_Date("AA20282/WG2414", 2495, 65)
{
 Outlier("General", 0.05);
};
R_Date("M-1719", 2460, 140)
{
 Outlier("Charcoal", 1);
};
R_Date("AA19681/WG2407", 2380, 50)
{
 Outlier("Charcoal", 1);
};
R_Date("M-1717", 2190, 140)
{
 Outlier("General", 0.05);
};
R_Date("M-1718", 2080, 140)
{
 Outlier("Charcoal", 1);
};
R_Date("M-1716", 2050, 130)
{
 Outlier("Charcoal", 1);
};
R_Date("M-1715", 1949, 130)
{
 Outlier("Charcoal", 1);
};
R_Simulate("Sim_RO1", -1004, 35);
R_Simulate("Sim_RO2", -1121, 35);
R_Simulate("Sim_RO3", -717, 35);
R_Simulate("Sim_RO4", -543, 35);
R_Simulate("Sim_RO5", -439, 35);
R_Simulate("Sim_RO6", -810, 35);
R_Simulate("Sim_RO7", -892, 35);
R_Simulate("Sim_RO8", -450, 35);
R_Simulate("Sim_RO9", -630, 35);
R_Simulate("Sim_RO10", -765, 35);
R_Simulate("Sim_RO11", -535, 35);
R_Simulate("Sim_RO12", -1054, 35);
```

```
R_Simulate("Sim_RO13", -270, 35);
R_Simulate("Sim_RO14", -447, 35);
R_Simulate("Sim_RO15", -670, 35);
R_Simulate("Sim_RO16", -582, 35);
R_Simulate("Sim_RO17", -203, 35);
R_Simulate("Sim_RO18", -1136, 35);
R_Simulate("Sim_RO19", -222, 35);
R_Simulate("Sim_RO20", -915, 35);
R_Simulate("Sim_RO21", -1023, 35);
R_Simulate("Sim_RO22", -345, 35);
R_Simulate("Sim_RO23", -744, 35);
R_Simulate("Sim_RO24", -772, 35);
R_Simulate("Sim_RO25", -360, 35);
R_Simulate("Sim_RO26", -211, 35);
R_Simulate("Sim_RO27", -781, 35);
R_Simulate("Sim_RO28", -841, 35);
R_Simulate("Sim_RO29", -338, 35);
R_Simulate("Sim_RO30", -552, 35);
R_Simulate("Sim_RO31", -182, 35);
R_Simulate("Sim_RO32", -104, 35);
R_Simulate("Sim_RO33", -805, 35);
R_Simulate("Sim_RO34", -510, 35);
R_Simulate("Sim_RO35", -876, 35);
R_Simulate("Sim_RO36", -132, 35);
R_Simulate("Sim_RO37", -141, 35);
R_Simulate("Sim_RO38", -742, 35);
R_Simulate("Sim_RO39", -882, 35);
R_Simulate("Sim_RO40", -947, 35);
R_Simulate("Sim_RO41", -661, 35);
R_Simulate("Sim_RO42", -949, 35);
R_Simulate("Sim_RO43", -687, 35);
R_Simulate("Sim_RO44", -985, 35);
R_Simulate("Sim_RO45", -173, 35);
R_Simulate("Sim_RO46", -467, 35);
R_Simulate("Sim_RO47", -472, 35);
R_Simulate("Sim_RO48", -744, 35);
R_Simulate("Sim_RO49", -863, 35);
R_Simulate("Sim_RO50", -658, 35);
R_Simulate("Sim_RO51", -937, 35);
R_Simulate("Sim_RO52", -930, 35);
R_Simulate("Sim_RO53", -935, 35);
R_Simulate("Sim_RO54", -750, 35);
R_Simulate("Sim_RO55", -155, 35);
R_Simulate("Sim_RO56", -695, 35);
R_Simulate("Sim_RO57", -591, 35);
R_Simulate("Sim_RO58", -579, 35);
```

```
  R_Simulate("Sim_RO59", -672, 35);
  R_Simulate("Sim_RO60", -674, 35);
  Interval ("Red Ochre");
 };
 Boundary("End Red Ochre")
 {
  Start("Start of End Red Ochre");
  Transition("Period of End Red Ochre");
  End("End of End Red Ochre");
 };
};
Sequence("Old Copper")
{
 Boundary("Start Old Copper")
 {
  Start("Start of Start Old Copper");
  Transition("Period of Start Old Copper");
  End("End of Start Old Copper");
 };
 Phase("Old Copper")
 {
  Sequence()
  {
   Boundary("Start Oconto");
   Phase("Oconto")
   {
    R_Date("AA19678/WG2404", 6020, 60)
    {
     Outlier("General", 0.05);
    };
    R_Date("AA20281/WG2413", 5250, 110)
    {
     Outlier("Charcoal", 1);
    };
   };
   Boundary("End Oconto");
  };
  Sequence()
  {
   Boundary("Start Allumette Island");
   Phase("Allumette Island")
   {
    R_Date("Beta-141985", 5440, 80)
    {
     Outlier("General", 0.05);
    };
```

```
  R_Date("S-509", 5240, 80)
  {
   Outlier("General", 0.05);
  };
  R_Date("Beta-141986", 5270, 40)
  {
   Outlier("General", 0.05);
  };
  R_Date("Beta-141987", 4680, 40)
  {
   Outlier("General", 0.05);
  };
 };
 Boundary("End Allumette Island");
};
Sequence()
{
 Boundary("Start Morrison Island");
 Phase("Morrison Island")
 {
  R_Date("Beta-88851", 4860, 50)
  {
   Outlier("General", 0.05);
  };
  R_Date("Beta-215300", 4820, 40)
  {
   Outlier("General", 0.05);
  };
  R_Date("Beta-215302", 4730, 40)
  {
   Outlier("General", 0.05);
  };
  R_Date("GSC-162", 4700, 150)
  {
   Outlier("Charcoal", 1);
  };
  R_Date("Beta-88852", 4630, 40)
  {
   Outlier("General", 0.05);
  };
  R_Date("Beta-88725", 4620, 40)
  {
   Outlier("General", 0.05);
  };
  R_Date("Beta-215301", 4210, 40)
  {
```

```
   Outlier("General", 0.05);
  };
 };
 Boundary("End Morrison Island");
};
R_Date("S-1263", 5000, 80)
{
 Outlier("General", 0.05);
};
R_Date("WIS-1706", 4080, 70)
{
 Outlier("General", 0.05);
};
R_Date("Beta-247459", 4490, 40)
{
 Outlier("Charcoal", 1);
};
R_Simulate("Sim_OC1", -2711, 35);
R_Simulate("Sim_OC2", -5008, 35);
R_Simulate("Sim_OC3", -2897, 35);
R_Simulate("Sim_OC4", -3077, 35);
R_Simulate("Sim_OC5", -4768, 35);
R_Simulate("Sim_OC6", -2580, 35);
R_Simulate("Sim_OC7", -3513, 35);
R_Simulate("Sim_OC8", -3842, 35);
R_Simulate("Sim_OC9", -2940, 35);
R_Simulate("Sim_OC10", -2627, 35);
R_Simulate("Sim_OC11", -4642, 35);
R_Simulate("Sim_OC12", -3581, 35);
R_Simulate("Sim_OC13", -2603, 35);
R_Simulate("Sim_OC14", -2707, 35);
R_Simulate("Sim_OC15", -2890, 35);
R_Simulate("Sim_OC16", -3895, 35);
R_Simulate("Sim_OC17", -5049, 35);
R_Simulate("Sim_OC18", -4539, 35);
R_Simulate("Sim_OC19", -3860, 35);
R_Simulate("Sim_OC20", -3416, 35);
R_Simulate("Sim_OC21", -3106, 35);
R_Simulate("Sim_OC22", -4723, 35);
R_Simulate("Sim_OC23", -4560, 35);
R_Simulate("Sim_OC24", -2864, 35);
R_Simulate("Sim_OC25", -2826, 35);
R_Simulate("Sim_OC26", -4350, 35);
R_Simulate("Sim_OC27", -3188, 35);
R_Simulate("Sim_OC28", -3735, 35);
R_Simulate("Sim_OC29", -3817, 35);
```

```
  R_Simulate("Sim_OC30", -3936, 35);
  R_Simulate("Sim_OC31", -3082, 35);
  R_Simulate("Sim_OC32", -3825, 35);
  R_Simulate("Sim_OC33", -4570, 35);
  R_Simulate("Sim_OC34", -4330, 35);
  R_Simulate("Sim_OC35", -3231, 35);
  R_Simulate("Sim_OC36", -2756, 35);
  R_Simulate("Sim_OC37", -2790, 35);
  R_Simulate("Sim_OC38", -4471, 35);
  R_Simulate("Sim_OC39", -3069, 35);
  R_Simulate("Sim_OC40", -5091, 35);
  R_Simulate("Sim_OC41", -3902, 35);
  R_Simulate("Sim_OC42", -4239, 35);
  R_Simulate("Sim_OC43", -2627, 35);
  R_Simulate("Sim_OC44", -2700, 35);
  R_Simulate("Sim_OC45", -2917, 35);
  R_Simulate("Sim_OC46", -3794, 35);
  R_Simulate("Sim_OC47", -5123, 35);
  R_Simulate("Sim_OC48", -5027, 35);
  R_Simulate("Sim_OC49", -4182, 35);
  R_Simulate("Sim_OC50", -2768, 35);
  R_Simulate("Sim_OC51", -4727, 35);
  R_Simulate("Sim_OC52", -5129, 35);
  R_Simulate("Sim_OC53", -4549, 35);
  R_Simulate("Sim_OC54", -5040, 35);
  R_Simulate("Sim_OC55", -4883, 35);
  R_Simulate("Sim_OC56", -3320, 35);
  R_Simulate("Sim_OC57", -5125, 35);
  R_Simulate("Sim_OC58", -3899, 35);
  R_Simulate("Sim_OC59", -3575, 35);
  R_Simulate("Sim_OC60", -2817, 35);
  Interval ("Old Copper");
 };
 Boundary("End Old Copper")
 {
  Start("Start of End Old Copper");
  Transition("Period of End Old Copper");
  End("End of End Old Copper");
 };
};
Sequence("Burnt Rollways")
{
 Boundary("Start Burnt Rollways")
 {
  Start("Start of Start Burnt Rollways");
  Transition("Period of Start Burnt Rollways");
```

```
 End("End of Start Burnt Rollways");
};
Phase("Burnt Rollways")
{
 Sequence()
 {
  Boundary("Start Duck Lake");
  Phase("Duck Lake")
  {
   R_Date("Beta-099777", 3420, 50)
   {
    Outlier("Charcoal", 1);
   };
   R_Date("Beta-124454", 3400, 110)
   {
    Outlier("Charcoal", 1);
   };
  };
  Boundary("End Duck Lake");
 };
 R_Date("WIS-2269", 3630, 60)
 {
  Outlier("Charcoal", 1);
 };
 R_Date("WIS-2270", 3270, 80)
 {
  Outlier("Charcoal", 1);
 };
 R_Date("Beta-232440", 2280, 40)
 {
  Outlier("Charcoal", 1);
 };
 R_Simulate("Sim_BR1", -1947, 35);
 R_Simulate("Sim_BR2", -1592, 35);
 R_Simulate("Sim_BR3", -2314, 35);
 R_Simulate("Sim_BR4", -1456, 35);
 R_Simulate("Sim_BR5", -2008, 35);
 R_Simulate("Sim_BR6", -647, 35);
 R_Simulate("Sim_BR7", -724, 35);
 R_Simulate("Sim_BR8", -1784, 35);
 R_Simulate("Sim_BR9", -493, 35);
 R_Simulate("Sim_BR10", -1588, 35);
 R_Simulate("Sim_BR11", -490, 35);
 R_Simulate("Sim_BR12", -631, 35);
 R_Simulate("Sim_BR13", -91, 35);
 R_Simulate("Sim_BR14", -426, 35);
```

```
R_Simulate("Sim_BR15", -1706, 35);
R_Simulate("Sim_BR16", -194, 35);
R_Simulate("Sim_BR17", -328, 35);
R_Simulate("Sim_BR18", -378, 35);
R_Simulate("Sim_BR19", -1103, 35);
R_Simulate("Sim_BR20", -2479, 35);
R_Simulate("Sim_BR21", -1069, 35);
R_Simulate("Sim_BR22", -99, 35);
R_Simulate("Sim_BR23", -610, 35);
R_Simulate("Sim_BR24", -135, 35);
R_Simulate("Sim_BR25", -1150, 35);
R_Simulate("Sim_BR26", -66, 35);
R_Simulate("Sim_BR27", -71, 35);
R_Simulate("Sim_BR28", -887, 35);
R_Simulate("Sim_BR29", -1756, 35);
R_Simulate("Sim_BR30", -1784, 35);
R_Simulate("Sim_BR31", -321, 35);
R_Simulate("Sim_BR32", -1557, 35);
R_Simulate("Sim_BR33", -706, 35);
R_Simulate("Sim_BR34", -2339, 35);
R_Simulate("Sim_BR35", -1018, 35);
R_Simulate("Sim_BR36", -1636, 35);
R_Simulate("Sim_BR37", -705, 35);
R_Simulate("Sim_BR38", -1189, 35);
R_Simulate("Sim_BR39", -2287, 35);
R_Simulate("Sim_BR40", -493, 35);
R_Simulate("Sim_BR41", -481, 35);
R_Simulate("Sim_BR42", -311, 35);
R_Simulate("Sim_BR43", -421, 35);
R_Simulate("Sim_BR44", -955, 35);
R_Simulate("Sim_BR45", -79, 35);
R_Simulate("Sim_BR46", -352, 35);
R_Simulate("Sim_BR47", -968, 35);
R_Simulate("Sim_BR48", -882, 35);
R_Simulate("Sim_BR49", -994, 35);
R_Simulate("Sim_BR50", -1043, 35);
R_Simulate("Sim_BR51", -1662, 35);
R_Simulate("Sim_BR52", -1084, 35);
R_Simulate("Sim_BR53", -2235, 35);
R_Simulate("Sim_BR54", -1524, 35);
R_Simulate("Sim_BR55", -2463, 35);
R_Simulate("Sim_BR56", -1497, 35);
R_Simulate("Sim_BR57", -1076, 35);
R_Simulate("Sim_BR58", -2046, 35);
R_Simulate("Sim_BR59", -428, 35);
R_Simulate("Sim_BR60", -573, 35);
```

```
   Interval ("Burnt Rollways");
  };
  Boundary("End Burnt Rollways")
  {
   Start("Start of End Burnt Rollways");
   Transition("Period of End Burnt Rollways");
   End("End of End Burnt Rollways");
  };
 };
 Order()
 {
 };
};
```

Simulation Code: Plus 70 Simulated Dates for Each Tradition

```
Plot()
 {
  Outlier_Model("Charcoal",Exp(1,-10,0),U(0,3),"t");
  Outlier_Model("General",T(5),U(0,4),"t");
  Sequence("Red Ochre")
  {
   Boundary("Start Red Ochre")
   {
    Start("Start of Start Red Ochre");
    Transition("Period of Start Red Ochre");
    End("End of Start Red Ochre");
   };
   Phase("Red Ochre")
   {
    R_Date("M-658", 3040, 150)
    {
     Outlier("General", 0.05);
    };
    R_Date("AA19679/WG2405", 2960, 50)
    {
     Outlier("Charcoal", 1);
    };
    R_Date("AA19685/WG2411", 2850, 50)
    {
     Outlier("Charcoal", 1);
    };
    R_Date("AA19680/WG2406", 2790, 50)
    {
     Outlier("Charcoal", 1);
    };
    R_Date("AA19677/WG2403", 2780, 65)
    {
     Outlier("General", 0.05);
    };
    R_Date("AA19684/WG2410", 2710, 50)
    {
     Outlier("Charcoal", 1);
    };
    R_Date("AA19686/WG2412", 2690, 60)
    {
     Outlier("Charcoal", 1);
    };
    R_Date("AA19682/WG2408", 2605, 45)
    {
```

```
 Outlier("Charcoal", 1);
};
R_Date("AA19683/WG2409", 2605, 50)
{
 Outlier("General", 0.05);
};
R_Date("AA20282/WG2414", 2495, 65)
{
 Outlier("General", 0.05);
};
R_Date("M-1719", 2460, 140)
{
 Outlier("Charcoal", 1);
};
R_Date("AA19681/WG2407", 2380, 50)
{
 Outlier("Charcoal", 1);
};
R_Date("M-1717", 2190, 140)
{
 Outlier("General", 0.05);
};
R_Date("M-1718", 2080, 140)
{
 Outlier("Charcoal", 1);
};
R_Date("M-1716", 2050, 130)
{
 Outlier("Charcoal", 1);
};
R_Date("M-1715", 1949, 130)
{
 Outlier("Charcoal", 1);
};
R_Simulate("Sim_RO1", -923, 35);
R_Simulate("Sim_RO2", -282, 35);
R_Simulate("Sim_RO3", -686, 35);
R_Simulate("Sim_RO4", -1027, 35);
R_Simulate("Sim_RO5", -1143, 35);
R_Simulate("Sim_RO6", -1091, 35);
R_Simulate("Sim_RO7", -972, 35);
R_Simulate("Sim_RO8", -795, 35);
R_Simulate("Sim_RO9", -199, 35);
R_Simulate("Sim_RO10", -1107, 35);
R_Simulate("Sim_RO11", -985, 35);
R_Simulate("Sim_RO12", -428, 35);
```

```
R_Simulate("Sim_RO13", -121, 35);
R_Simulate("Sim_RO14", -473, 35);
R_Simulate("Sim_RO15", -1149, 35);
R_Simulate("Sim_RO16", -620, 35);
R_Simulate("Sim_RO17", -427, 35);
R_Simulate("Sim_RO18", -486, 35);
R_Simulate("Sim_RO19", -140, 35);
R_Simulate("Sim_RO20", -555, 35);
R_Simulate("Sim_RO21", -932, 35);
R_Simulate("Sim_RO22", -233, 35);
R_Simulate("Sim_RO23", -1010, 35);
R_Simulate("Sim_RO24", -611, 35);
R_Simulate("Sim_RO25", -1161, 35);
R_Simulate("Sim_RO26", -1052, 35);
R_Simulate("Sim_RO27", -1001, 35);
R_Simulate("Sim_RO28", -637, 35);
R_Simulate("Sim_RO29", -1113, 35);
R_Simulate("Sim_RO30", -434, 35);
R_Simulate("Sim_RO31", -787, 35);
R_Simulate("Sim_RO32", -687, 35);
R_Simulate("Sim_RO33", -551, 35);
R_Simulate("Sim_RO34", -304, 35);
R_Simulate("Sim_RO35", -256, 35);
R_Simulate("Sim_RO36", -275, 35);
R_Simulate("Sim_RO37", -191, 35);
R_Simulate("Sim_RO38", -181, 35);
R_Simulate("Sim_RO39", -953, 35);
R_Simulate("Sim_RO40", -991, 35);
R_Simulate("Sim_RO41", -491, 35);
R_Simulate("Sim_RO42", -241, 35);
R_Simulate("Sim_RO43", -899, 35);
R_Simulate("Sim_RO44", -556, 35);
R_Simulate("Sim_RO45", -973, 35);
R_Simulate("Sim_RO46", -291, 35);
R_Simulate("Sim_RO47", -1120, 35);
R_Simulate("Sim_RO48", -233, 35);
R_Simulate("Sim_RO49", -601, 35);
R_Simulate("Sim_RO50", -326, 35);
R_Simulate("Sim_RO51", -248, 35);
R_Simulate("Sim_RO52", -197, 35);
R_Simulate("Sim_RO53", -501, 35);
R_Simulate("Sim_RO54", -888, 35);
R_Simulate("Sim_RO55", -973, 35);
R_Simulate("Sim_RO56", -338, 35);
R_Simulate("Sim_RO57", -313, 35);
R_Simulate("Sim_RO58", -1169, 35);
```

```
  R_Simulate("Sim_RO59", -541, 35);
  R_Simulate("Sim_RO60", -650, 35);
  R_Simulate("Sim_RO61", -1162, 35);
  R_Simulate("Sim_RO62", -204, 35);
  R_Simulate("Sim_RO63", -891, 35);
  R_Simulate("Sim_RO64", -991, 35);
  R_Simulate("Sim_RO65", -600, 35);
  R_Simulate("Sim_RO66", -1043, 35);
  R_Simulate("Sim_RO67", -696, 35);
  R_Simulate("Sim_RO68", -1100, 35);
  R_Simulate("Sim_RO69", -220, 35);
  R_Simulate("Sim_RO70", -1116, 35);
  Interval ("Red Ochre");
 };
 Boundary("End Red Ochre")
 {
  Start("Start of End Red Ochre");
  Transition("Period of End Red Ochre");
  End("End of End Red Ochre");
 };
};
Sequence("Old Copper")
{
 Boundary("Start Old Copper")
 {
  Start("Start of Start Old Copper");
  Transition("Period of Start Old Copper");
  End("End of Start Old Copper");
 };
 Phase("Old Copper")
 {
  Sequence()
  {
   Boundary("Start Oconto");
   Phase("Oconto")
   {
    R_Date("AA19678/WG2404", 6020, 60)
    {
     Outlier("General", 0.05);
    };
    R_Date("AA20281/WG2413", 5250, 110)
    {
     Outlier("Charcoal", 1);
    };
   };
   Boundary("End Oconto");
```

```
};
Sequence()
{
 Boundary("Start Allumette Island");
 Phase("Allumette Island")
 {
  R_Date("Beta-141985", 5440, 80)
  {
   Outlier("General", 0.05);
  };
  R_Date("S-509", 5240, 80)
  {
   Outlier("General", 0.05);
  };
  R_Date("Beta-141986", 5270, 40)
  {
   Outlier("General", 0.05);
  };
  R_Date("Beta-141987", 4680, 40)
  {
   Outlier("General", 0.05);
  };
 };
 Boundary("End Allumette Island");
};
Sequence()
{
 Boundary("Start Morrison Island");
 Phase("Morrison Island")
 {
  R_Date("Beta-88851", 4860, 50)
  {
   Outlier("General", 0.05);
  };
  R_Date("Beta-215300", 4820, 40)
  {
   Outlier("General", 0.05);
  };
  R_Date("Beta-215302", 4730, 40)
  {
   Outlier("General", 0.05);
  };
  R_Date("GSC-162", 4700, 150)
  {
   Outlier("Charcoal", 1);
  };
```

```
  R_Date("Beta-88852", 4630, 40)
  {
   Outlier("General", 0.05);
  };
  R_Date("Beta-88725", 4620, 40)
  {
   Outlier("General", 0.05);
  };
  R_Date("Beta-215301", 4210, 40)
  {
   Outlier("General", 0.05);
  };
 };
 Boundary("End Morrison Island");
};
R_Date("S-1263", 5000, 80)
{
 Outlier("General", 0.05);
};
R_Date("WIS-1706", 4080, 70)
{
 Outlier("General", 0.05);
};
R_Date("Beta-247459", 4490, 40)
{
 Outlier("Charcoal", 1);
};
R_Simulate("Sim_OC1", -3558, 35);
R_Simulate("Sim_OC2", -4332, 35);
R_Simulate("Sim_OC3", -4496, 35);
R_Simulate("Sim_OC4", -2715, 35);
R_Simulate("Sim_OC5", -2834, 35);
R_Simulate("Sim_OC6", -4998, 35);
R_Simulate("Sim_OC7", -4956, 35);
R_Simulate("Sim_OC8", -3917, 35);
R_Simulate("Sim_OC9", -3964, 35);
R_Simulate("Sim_OC10", -3151, 35);
R_Simulate("Sim_OC11", -4297, 35);
R_Simulate("Sim_OC12", -3527, 35);
R_Simulate("Sim_OC13", -5076, 35);
R_Simulate("Sim_OC14", -4249, 35);
R_Simulate("Sim_OC15", -3096, 35);
R_Simulate("Sim_OC16", -2786, 35);
R_Simulate("Sim_OC17", -3492, 35);
R_Simulate("Sim_OC18", -2853, 35);
R_Simulate("Sim_OC19", -3753, 35);
```

```
R_Simulate("Sim_OC20", -2641, 35);
R_Simulate("Sim_OC21", -4652, 35);
R_Simulate("Sim_OC22", -3647, 35);
R_Simulate("Sim_OC23", -3155, 35);
R_Simulate("Sim_OC24", -4717, 35);
R_Simulate("Sim_OC25", -3688, 35);
R_Simulate("Sim_OC26", -4066, 35);
R_Simulate("Sim_OC27", -2764, 35);
R_Simulate("Sim_OC28", -4135, 35);
R_Simulate("Sim_OC29", -3030, 35);
R_Simulate("Sim_OC30", -4290, 35);
R_Simulate("Sim_OC31", -2542, 35);
R_Simulate("Sim_OC32", -4121, 35);
R_Simulate("Sim_OC33", -4649, 35);
R_Simulate("Sim_OC34", -3499, 35);
R_Simulate("Sim_OC35", -2858, 35);
R_Simulate("Sim_OC36", -4293, 35);
R_Simulate("Sim_OC37", -2999, 35);
R_Simulate("Sim_OC38", -4073, 35);
R_Simulate("Sim_OC39", -3032, 35);
R_Simulate("Sim_OC40", -4784, 35);
R_Simulate("Sim_OC41", -2985, 35);
R_Simulate("Sim_OC42", -2629, 35);
R_Simulate("Sim_OC43", -3392, 35);
R_Simulate("Sim_OC44", -4524, 35);
R_Simulate("Sim_OC45", -4191, 35);
R_Simulate("Sim_OC46", -2830, 35);
R_Simulate("Sim_OC47", -3077, 35);
R_Simulate("Sim_OC48", -3163, 35);
R_Simulate("Sim_OC49", -3417, 35);
R_Simulate("Sim_OC50", -3956, 35);
R_Simulate("Sim_OC51", -3842, 35);
R_Simulate("Sim_OC52", -3217, 35);
R_Simulate("Sim_OC53", -2661, 35);
R_Simulate("Sim_OC54", -2628, 35);
R_Simulate("Sim_OC55", -4859, 35);
R_Simulate("Sim_OC56", -4628, 35);
R_Simulate("Sim_OC57", -5013, 35);
R_Simulate("Sim_OC58", -4584, 35);
R_Simulate("Sim_OC59", -4445, 35);
R_Simulate("Sim_OC60", -3631, 35);
R_Simulate("Sim_OC61", -3292, 35);
R_Simulate("Sim_OC62", -3696, 35);
R_Simulate("Sim_OC63", -2603, 35);
R_Simulate("Sim_OC64", -2613, 35);
R_Simulate("Sim_OC65", -2510, 35);
```

```
  R_Simulate("Sim_OC66", -4815, 35);
  R_Simulate("Sim_OC67", -3262, 35);
  R_Simulate("Sim_OC68", -5043, 35);
  R_Simulate("Sim_OC69", -3055, 35);
  R_Simulate("Sim_OC70", -3146, 35);
  Interval ("Old Copper");
 };
 Boundary("End Old Copper")
 {
  Start("Start of End Old Copper");
  Transition("Period of End Old Copper");
  End("End of End Old Copper");
 };
};
Sequence("Burnt Rollways")
{
 Boundary("Start Burnt Rollways")
 {
  Start("Start of Start Burnt Rollways");
  Transition("Period of Start Burnt Rollways");
  End("End of Start Burnt Rollways");
 };
 Phase("Burnt Rollways")
 {
  Sequence()
  {
   Boundary("Start Duck Lake");
   Phase("Duck Lake")
   {
    R_Date("Beta-099777", 3420, 50)
    {
     Outlier("Charcoal", 1);
    };
    R_Date("Beta-124454", 3400, 110)
    {
     Outlier("Charcoal", 1);
    };
   };
   Boundary("End Duck Lake");
  };
  R_Date("WIS-2269", 3630, 60)
  {
   Outlier("Charcoal", 1);
  };
  R_Date("WIS-2270", 3270, 80)
  {
```

```
 Outlier("Charcoal", 1);
};
R_Date("Beta-232440", 2280, 40)
{
 Outlier("Charcoal", 1);
};
R_Simulate("Sim_BR1", -2339, 35);
R_Simulate("Sim_BR2", -2153, 35);
R_Simulate("Sim_BR3", -1880, 35);
R_Simulate("Sim_BR4", -1981, 35);
R_Simulate("Sim_BR5", -1108, 35);
R_Simulate("Sim_BR6", -1495, 35);
R_Simulate("Sim_BR7", -2102, 35);
R_Simulate("Sim_BR8", -955, 35);
R_Simulate("Sim_BR9", -1163, 35);
R_Simulate("Sim_BR10", -1772, 35);
R_Simulate("Sim_BR11", -1184, 35);
R_Simulate("Sim_BR12", -2037, 35);
R_Simulate("Sim_BR13", -1875, 35);
R_Simulate("Sim_BR14", -931, 35);
R_Simulate("Sim_BR15", -1341, 35);
R_Simulate("Sim_BR16", -2027, 35);
R_Simulate("Sim_BR17", -2245, 35);
R_Simulate("Sim_BR18", -1156, 35);
R_Simulate("Sim_BR19", -260, 35);
R_Simulate("Sim_BR20", -2338, 35);
R_Simulate("Sim_BR21", -889, 35);
R_Simulate("Sim_BR22", -910, 35);
R_Simulate("Sim_BR23", -1623, 35);
R_Simulate("Sim_BR24", -2035, 35);
R_Simulate("Sim_BR25", -394, 35);
R_Simulate("Sim_BR26", -2088, 35);
R_Simulate("Sim_BR27", -190, 35);
R_Simulate("Sim_BR28", -2304, 35);
R_Simulate("Sim_BR29", -1022, 35);
R_Simulate("Sim_BR30", -718, 35);
R_Simulate("Sim_BR31", -1257, 35);
R_Simulate("Sim_BR32", -2001, 35);
R_Simulate("Sim_BR33", -373, 35);
R_Simulate("Sim_BR34", -412, 35);
R_Simulate("Sim_BR35", -195, 35);
R_Simulate("Sim_BR36", -1754, 35);
R_Simulate("Sim_BR37", -647, 35);
R_Simulate("Sim_BR38", -1733, 35);
R_Simulate("Sim_BR39", -321, 35);
R_Simulate("Sim_BR40", -1675, 35);
```

```
   R_Simulate("Sim_BR41", -468, 35);
   R_Simulate("Sim_BR42", -2267, 35);
   R_Simulate("Sim_BR43", -846, 35);
   R_Simulate("Sim_BR44", -2008, 35);
   R_Simulate("Sim_BR45", -1513, 35);
   R_Simulate("Sim_BR46", -1127, 35);
   R_Simulate("Sim_BR47", -2457, 35);
   R_Simulate("Sim_BR48", -1739, 35);
   R_Simulate("Sim_BR49", -1098, 35);
   R_Simulate("Sim_BR50", -1340, 35);
   R_Simulate("Sim_BR51", -2195, 35);
   R_Simulate("Sim_BR52", -909, 35);
   R_Simulate("Sim_BR53", -2394, 35);
   R_Simulate("Sim_BR54", -171, 35);
   R_Simulate("Sim_BR55", -1506, 35);
   R_Simulate("Sim_BR56", -1582, 35);
   R_Simulate("Sim_BR57", -2483, 35);
   R_Simulate("Sim_BR58", -2179, 35);
   R_Simulate("Sim_BR59", -2132, 35);
   R_Simulate("Sim_BR60", -1982, 35);
   R_Simulate("Sim_BR61", -1467, 35);
   R_Simulate("Sim_BR62", -1414, 35);
   R_Simulate("Sim_BR63", -986, 35);
   R_Simulate("Sim_BR64", -1451, 35);
   R_Simulate("Sim_BR65", -2211, 35);
   R_Simulate("Sim_BR66", -1030, 35);
   R_Simulate("Sim_BR67", -1708, 35);
   R_Simulate("Sim_BR68", -1024, 35);
   R_Simulate("Sim_BR69", -2075, 35);
   R_Simulate("Sim_BR70", -1404, 35);
   Interval ("Burnt Rollways");
  };
  Boundary("End Burnt Rollways")
  {
   Start("Start of End Burnt Rollways");
   Transition("Period of End Burnt Rollways");
   End("End of End Burnt Rollways");
  };
 };
 Order()
 {
 };
};
```

Simulation Code: Plus 80 Simulated Dates for Each Tradition

```
Plot()
 {
  Outlier_Model("Charcoal",Exp(1,-10,0),U(0,3),"t");
  Outlier_Model("General",T(5),U(0,4),"t");
  Sequence("Red Ochre")
  {
   Boundary("Start Red Ochre")
   {
    Start("Start of Start Red Ochre");
    Transition("Period of Start Red Ochre");
    End("End of Start Red Ochre");
   };
   Phase("Red Ochre")
   {
    R_Date("M-658", 3040, 150)
    {
     Outlier("General", 0.05);
    };
    R_Date("AA19679/WG2405", 2960, 50)
    {
     Outlier("Charcoal", 1);
    };
    R_Date("AA19685/WG2411", 2850, 50)
    {
     Outlier("Charcoal", 1);
    };
    R_Date("AA19680/WG2406", 2790, 50)
    {
     Outlier("Charcoal", 1);
    };
    R_Date("AA19677/WG2403", 2780, 65)
    {
     Outlier("General", 0.05);
    };
    R_Date("AA19684/WG2410", 2710, 50)
    {
     Outlier("Charcoal", 1);
    };
    R_Date("AA19686/WG2412", 2690, 60)
    {
     Outlier("Charcoal", 1);
    };
    R_Date("AA19682/WG2408", 2605, 45)
    {
```

```
 Outlier("Charcoal", 1);
};
R_Date("AA19683/WG2409", 2605, 50)
{
 Outlier("General", 0.05);
};
R_Date("AA20282/WG2414", 2495, 65)
{
 Outlier("General", 0.05);
};
R_Date("M-1719", 2460, 140)
{
 Outlier("Charcoal", 1);
};
R_Date("AA19681/WG2407", 2380, 50)
{
 Outlier("Charcoal", 1);
};
R_Date("M-1717", 2190, 140)
{
 Outlier("General", 0.05);
};
R_Date("M-1718", 2080, 140)
{
 Outlier("Charcoal", 1);
};
R_Date("M-1716", 2050, 130)
{
 Outlier("Charcoal", 1);
};
R_Date("M-1715", 1949, 130)
{
 Outlier("Charcoal", 1);
};
R_Simulate("Sim_RO1", -248, 35);
R_Simulate("Sim_RO2", -624, 35);
R_Simulate("Sim_RO3", -289, 35);
R_Simulate("Sim_RO4", -1074, 35);
R_Simulate("Sim_RO5", -340, 35);
R_Simulate("Sim_RO6", -980, 35);
R_Simulate("Sim_RO7", -1020, 35);
R_Simulate("Sim_RO8", -666, 35);
R_Simulate("Sim_RO9", -377, 35);
R_Simulate("Sim_RO10", -277, 35);
R_Simulate("Sim_RO11", -281, 35);
R_Simulate("Sim_RO12", -913, 35);
```

```
R_Simulate("Sim_RO13", -247, 35);
R_Simulate("Sim_RO14", -121, 35);
R_Simulate("Sim_RO15", -340, 35);
R_Simulate("Sim_RO16", -330, 35);
R_Simulate("Sim_RO17", -403, 35);
R_Simulate("Sim_RO18", -113, 35);
R_Simulate("Sim_RO19", -589, 35);
R_Simulate("Sim_RO20", -459, 35);
R_Simulate("Sim_RO21", -441, 35);
R_Simulate("Sim_RO22", -419, 35);
R_Simulate("Sim_RO23", -517, 35);
R_Simulate("Sim_RO24", -641, 35);
R_Simulate("Sim_RO25", -651, 35);
R_Simulate("Sim_RO26", -266, 35);
R_Simulate("Sim_RO27", -237, 35);
R_Simulate("Sim_RO28", -832, 35);
R_Simulate("Sim_RO29", -189, 35);
R_Simulate("Sim_RO30", -1135, 35);
R_Simulate("Sim_RO31", -387, 35);
R_Simulate("Sim_RO32", -491, 35);
R_Simulate("Sim_RO33", -579, 35);
R_Simulate("Sim_RO34", -316, 35);
R_Simulate("Sim_RO35", -420, 35);
R_Simulate("Sim_RO36", -430, 35);
R_Simulate("Sim_RO37", -1100, 35);
R_Simulate("Sim_RO38", -969, 35);
R_Simulate("Sim_RO39", -650, 35);
R_Simulate("Sim_RO40", -862, 35);
R_Simulate("Sim_RO41", -950, 35);
R_Simulate("Sim_RO42", -143, 35);
R_Simulate("Sim_RO43", -426, 35);
R_Simulate("Sim_RO44", -998, 35);
R_Simulate("Sim_RO45", -616, 35);
R_Simulate("Sim_RO46", -351, 35);
R_Simulate("Sim_RO47", -390, 35);
R_Simulate("Sim_RO48", -321, 35);
R_Simulate("Sim_RO49", -521, 35);
R_Simulate("Sim_RO50", -902, 35);
R_Simulate("Sim_RO51", -855, 35);
R_Simulate("Sim_RO52", -192, 35);
R_Simulate("Sim_RO53", -832, 35);
R_Simulate("Sim_RO54", -517, 35);
R_Simulate("Sim_RO55", -341, 35);
R_Simulate("Sim_RO56", -707, 35);
R_Simulate("Sim_RO57", -211, 35);
R_Simulate("Sim_RO58", -228, 35);
```

```
  R_Simulate("Sim_RO59", -523, 35);
  R_Simulate("Sim_RO60", -117, 35);
  R_Simulate("Sim_RO61", -604, 35);
  R_Simulate("Sim_RO62", -301, 35);
  R_Simulate("Sim_RO63", -405, 35);
  R_Simulate("Sim_RO64", -758, 35);
  R_Simulate("Sim_RO65", -724, 35);
  R_Simulate("Sim_RO66", -354, 35);
  R_Simulate("Sim_RO67", -299, 35);
  R_Simulate("Sim_RO68", -416, 35);
  R_Simulate("Sim_RO69", -950, 35);
  R_Simulate("Sim_RO70", -547, 35);
  R_Simulate("Sim_RO71", -847, 35);
  R_Simulate("Sim_RO72", -1135, 35);
  R_Simulate("Sim_RO73", -631, 35);
  R_Simulate("Sim_RO74", -434, 35);
  R_Simulate("Sim_RO75", -273, 35);
  R_Simulate("Sim_RO76", -851, 35);
  R_Simulate("Sim_RO77", -1019, 35);
  R_Simulate("Sim_RO78", -875, 35);
  R_Simulate("Sim_RO79", -551, 35);
  R_Simulate("Sim_RO80", -1140, 35);
  Interval ("Red Ochre");
 };
 Boundary("End Red Ochre")
 {
  Start("Start of End Red Ochre");
  Transition("Period of End Red Ochre");
  End("End of End Red Ochre");
 };
};
Sequence("Old Copper")
{
 Boundary("Start Old Copper")
 {
  Start("Start of Start Old Copper");
  Transition("Period of Start Old Copper");
  End("End of Start Old Copper");
 };
 Phase("Old Copper")
 {
  Sequence()
  {
   Boundary("Start Oconto");
   Phase("Oconto")
   {
```

```
  R_Date("AA19678/WG2404", 6020, 60)
  {
   Outlier("General", 0.05);
  };
  R_Date("AA20281/WG2413", 5250, 110)
  {
   Outlier("Charcoal", 1);
  };
 };
 Boundary("End Oconto");
};
Sequence()
{
 Boundary("Start Allumette Island");
 Phase("Allumette Island")
 {
  R_Date("Beta-141985", 5440, 80)
  {
   Outlier("General", 0.05);
  };
  R_Date("S-509", 5240, 80)
  {
   Outlier("General", 0.05);
  };
  R_Date("Beta-141986", 5270, 40)
  {
   Outlier("General", 0.05);
  };
  R_Date("Beta-141987", 4680, 40)
  {
   Outlier("General", 0.05);
  };
 };
 Boundary("End Allumette Island");
};
Sequence()
{
 Boundary("Start Morrison Island");
 Phase("Morrison Island")
 {
  R_Date("Beta-88851", 4860, 50)
  {
   Outlier("General", 0.05);
  };
  R_Date("Beta-215300", 4820, 40)
  {
```

```
   Outlier("General", 0.05);
  };
  R_Date("Beta-215302", 4730, 40)
  {
   Outlier("General", 0.05);
  };
  R_Date("GSC-162", 4700, 150)
  {
   Outlier("Charcoal", 1);
  };
  R_Date("Beta-88852", 4630, 40)
  {
   Outlier("General", 0.05);
  };
  R_Date("Beta-88725", 4620, 40)
  {
   Outlier("General", 0.05);
  };
  R_Date("Beta-215301", 4210, 40)
  {
   Outlier("General", 0.05);
  };
 };
 Boundary("End Morrison Island");
};
R_Date("S-1263", 5000, 80)
{
 Outlier("General", 0.05);
};
R_Date("WIS-1706", 4080, 70)
{
 Outlier("General", 0.05);
};
R_Date("Beta-247459", 4490, 40)
{
 Outlier("Charcoal", 1);
};
R_Simulate("Sim_OC1", -4458, 35);
R_Simulate("Sim_OC2", -2876, 35);
R_Simulate("Sim_OC3", -2914, 35);
R_Simulate("Sim_OC4", -4275, 35);
R_Simulate("Sim_OC5", -4609, 35);
R_Simulate("Sim_OC6", -2669, 35);
R_Simulate("Sim_OC7", -3421, 35);
R_Simulate("Sim_OC8", -4304, 35);
R_Simulate("Sim_OC9", -5043, 35);
```

```
R_Simulate("Sim_OC10", -3328, 35);
R_Simulate("Sim_OC11", -3862, 35);
R_Simulate("Sim_OC12", -3918, 35);
R_Simulate("Sim_OC13", -4963, 35);
R_Simulate("Sim_OC14", -3710, 35);
R_Simulate("Sim_OC15", -4506, 35);
R_Simulate("Sim_OC16", -3071, 35);
R_Simulate("Sim_OC17", -2513, 35);
R_Simulate("Sim_OC18", -4968, 35);
R_Simulate("Sim_OC19", -3177, 35);
R_Simulate("Sim_OC20", -3881, 35);
R_Simulate("Sim_OC21", -2696, 35);
R_Simulate("Sim_OC22", -4742, 35);
R_Simulate("Sim_OC23", -2952, 35);
R_Simulate("Sim_OC24", -5091, 35);
R_Simulate("Sim_OC25", -3916, 35);
R_Simulate("Sim_OC26", -3379, 35);
R_Simulate("Sim_OC27", -3847, 35);
R_Simulate("Sim_OC28", -2922, 35);
R_Simulate("Sim_OC29", -2981, 35);
R_Simulate("Sim_OC30", -4987, 35);
R_Simulate("Sim_OC31", -4294, 35);
R_Simulate("Sim_OC32", -2511, 35);
R_Simulate("Sim_OC33", -4141, 35);
R_Simulate("Sim_OC34", -2795, 35);
R_Simulate("Sim_OC35", -2854, 35);
R_Simulate("Sim_OC36", -4191, 35);
R_Simulate("Sim_OC37", -5028, 35);
R_Simulate("Sim_OC38", -3793, 35);
R_Simulate("Sim_OC39", -4056, 35);
R_Simulate("Sim_OC40", -4339, 35);
R_Simulate("Sim_OC41", -4549, 35);
R_Simulate("Sim_OC42", -3989, 35);
R_Simulate("Sim_OC43", -2658, 35);
R_Simulate("Sim_OC44", -4766, 35);
R_Simulate("Sim_OC45", -3608, 35);
R_Simulate("Sim_OC46", -2640, 35);
R_Simulate("Sim_OC47", -3506, 35);
R_Simulate("Sim_OC48", -3595, 35);
R_Simulate("Sim_OC49", -4257, 35);
R_Simulate("Sim_OC50", -4826, 35);
R_Simulate("Sim_OC51", -4141, 35);
R_Simulate("Sim_OC52", -3473, 35);
R_Simulate("Sim_OC53", -4990, 35);
R_Simulate("Sim_OC54", -4229, 35);
R_Simulate("Sim_OC55", -4530, 35);
```

```
  R_Simulate("Sim_OC56", -2951, 35);
  R_Simulate("Sim_OC57", -4044, 35);
  R_Simulate("Sim_OC58", -4179, 35);
  R_Simulate("Sim_OC59", -3907, 35);
  R_Simulate("Sim_OC60", -2619, 35);
  R_Simulate("Sim_OC61", -4032, 35);
  R_Simulate("Sim_OC62", -4728, 35);
  R_Simulate("Sim_OC63", -3660, 35);
  R_Simulate("Sim_OC64", -5067, 35);
  R_Simulate("Sim_OC65", -4673, 35);
  R_Simulate("Sim_OC66", -4716, 35);
  R_Simulate("Sim_OC67", -4890, 35);
  R_Simulate("Sim_OC68", -4895, 35);
  R_Simulate("Sim_OC69", -2685, 35);
  R_Simulate("Sim_OC70", -3988, 35);
  R_Simulate("Sim_OC71", -5097, 35);
  R_Simulate("Sim_OC72", -2891, 35);
  R_Simulate("Sim_OC73", -4947, 35);
  R_Simulate("Sim_OC74", -5075, 35);
  R_Simulate("Sim_OC75", -3195, 35);
  R_Simulate("Sim_OC76", -3509, 35);
  R_Simulate("Sim_OC77", -3108, 35);
  R_Simulate("Sim_OC78", -2733, 35);
  R_Simulate("Sim_OC79", -3373, 35);
  R_Simulate("Sim_OC80", -3564, 35);
  Interval ("Old Copper");
 };
 Boundary("End Old Copper")
 {
  Start("Start of End Old Copper");
  Transition("Period of End Old Copper");
  End("End of End Old Copper");
 };
};
Sequence("Burnt Rollways")
{
 Boundary("Start Burnt Rollways")
 {
  Start("Start of Start Burnt Rollways");
  Transition("Period of Start Burnt Rollways");
  End("End of Start Burnt Rollways");
 };
 Phase("Burnt Rollways")
 {
  Sequence()
  {
```

```
 Boundary("Start Duck Lake");
 Phase("Duck Lake")
 {
  R_Date("Beta-099777", 3420, 50)
  {
   Outlier("Charcoal", 1);
  };
  R_Date("Beta-124454", 3400, 110)
  {
   Outlier("Charcoal", 1);
  };
 };
 Boundary("End Duck Lake");
};
R_Date("WIS-2269", 3630, 60)
{
 Outlier("Charcoal", 1);
};
R_Date("WIS-2270", 3270, 80)
{
 Outlier("Charcoal", 1);
};
R_Date("Beta-232440", 2280, 40)
{
 Outlier("Charcoal", 1);
};
R_Simulate("Sim_BR1", -2379, 35);
R_Simulate("Sim_BR2", -1920, 35);
R_Simulate("Sim_BR3", -674, 35);
R_Simulate("Sim_BR4", -1219, 35);
R_Simulate("Sim_BR5", -1332, 35);
R_Simulate("Sim_BR6", -1423, 35);
R_Simulate("Sim_BR7", -673, 35);
R_Simulate("Sim_BR8", -1823, 35);
R_Simulate("Sim_BR9", -1955, 35);
R_Simulate("Sim_BR10", -2472, 35);
R_Simulate("Sim_BR11", -1531, 35);
R_Simulate("Sim_BR12", -2445, 35);
R_Simulate("Sim_BR13", -305, 35);
R_Simulate("Sim_BR14", -1240, 35);
R_Simulate("Sim_BR15", -1247, 35);
R_Simulate("Sim_BR16", -1096, 35);
R_Simulate("Sim_BR17", -560, 35);
R_Simulate("Sim_BR18", -857, 35);
R_Simulate("Sim_BR19", -1298, 35);
R_Simulate("Sim_BR20", -2265, 35);
```

```
R_Simulate("Sim_BR21", -687, 35);
R_Simulate("Sim_BR22", -1719, 35);
R_Simulate("Sim_BR23", -1707, 35);
R_Simulate("Sim_BR24", -1039, 35);
R_Simulate("Sim_BR25", -908, 35);
R_Simulate("Sim_BR26", -258, 35);
R_Simulate("Sim_BR27", -908, 35);
R_Simulate("Sim_BR28", -1645, 35);
R_Simulate("Sim_BR29", -1961, 35);
R_Simulate("Sim_BR30", -2028, 35);
R_Simulate("Sim_BR31", -1290, 35);
R_Simulate("Sim_BR32", -887, 35);
R_Simulate("Sim_BR33", -1644, 35);
R_Simulate("Sim_BR34", -563, 35);
R_Simulate("Sim_BR35", -1327, 35);
R_Simulate("Sim_BR36", -1503, 35);
R_Simulate("Sim_BR37", -552, 35);
R_Simulate("Sim_BR38", -1347, 35);
R_Simulate("Sim_BR39", -1590, 35);
R_Simulate("Sim_BR40", -1255, 35);
R_Simulate("Sim_BR41", -1222, 35);
R_Simulate("Sim_BR42", -1471, 35);
R_Simulate("Sim_BR43", -856, 35);
R_Simulate("Sim_BR44", -132, 35);
R_Simulate("Sim_BR45", -667, 35);
R_Simulate("Sim_BR46", -1735, 35);
R_Simulate("Sim_BR47", -2279, 35);
R_Simulate("Sim_BR48", -1248, 35);
R_Simulate("Sim_BR49", -1600, 35);
R_Simulate("Sim_BR50", -1116, 35);
R_Simulate("Sim_BR51", -2390, 35);
R_Simulate("Sim_BR52", -2487, 35);
R_Simulate("Sim_BR53", -2259, 35);
R_Simulate("Sim_BR54", -78, 35);
R_Simulate("Sim_BR55", -204, 35);
R_Simulate("Sim_BR56", -1943, 35);
R_Simulate("Sim_BR57", -1563, 35);
R_Simulate("Sim_BR58", -1275, 35);
R_Simulate("Sim_BR59", -418, 35);
R_Simulate("Sim_BR60", -769, 35);
R_Simulate("Sim_BR61", -1613, 35);
R_Simulate("Sim_BR62", -1909, 35);
R_Simulate("Sim_BR63", -411, 35);
R_Simulate("Sim_BR64", -1371, 35);
R_Simulate("Sim_BR65", -1726, 35);
R_Simulate("Sim_BR66", -736, 35);
```

```
  R_Simulate("Sim_BR67", -2142, 35);
  R_Simulate("Sim_BR68", -1468, 35);
  R_Simulate("Sim_BR69", -2395, 35);
  R_Simulate("Sim_BR70", -866, 35);
  R_Simulate("Sim_BR71", -1783, 35);
  R_Simulate("Sim_BR72", -1771, 35);
  R_Simulate("Sim_BR73", -2259, 35);
  R_Simulate("Sim_BR74", -1609, 35);
  R_Simulate("Sim_BR75", -1778, 35);
  R_Simulate("Sim_BR76", -1868, 35);
  R_Simulate("Sim_BR77", -2471, 35);
  R_Simulate("Sim_BR78", -1825, 35);
  R_Simulate("Sim_BR79", -1988, 35);
  R_Simulate("Sim_BR80", -315, 35);
  Interval ("Burnt Rollways");
 };
 Boundary("End Burnt Rollways")
 {
  Start("Start of End Burnt Rollways");
  Transition("Period of End Burnt Rollways");
  End("End of End Burnt Rollways");
 };
};
Order()
{
};
};
```

Simulation Code: Plus 90 Simulated Dates for Each Tradition

```
Plot()
 {
  Outlier_Model("Charcoal",Exp(1,-10,0),U(0,3),"t");
  Outlier_Model("General",T(5),U(0,4),"t");
  Sequence("Red Ochre")
  {
   Boundary("Start Red Ochre")
   {
    Start("Start of Start Red Ochre");
    Transition("Period of Start Red Ochre");
    End("End of Start Red Ochre");
   };
   Phase("Red Ochre")
   {
    R_Date("M-658", 3040, 150)
    {
     Outlier("General", 0.05);
    };
    R_Date("AA19679/WG2405", 2960, 50)
    {
     Outlier("Charcoal", 1);
    };
    R_Date("AA19685/WG2411", 2850, 50)
    {
     Outlier("Charcoal", 1);
    };
    R_Date("AA19680/WG2406", 2790, 50)
    {
     Outlier("Charcoal", 1);
    };
    R_Date("AA19677/WG2403", 2780, 65)
    {
     Outlier("General", 0.05);
    };
    R_Date("AA19684/WG2410", 2710, 50)
    {
     Outlier("Charcoal", 1);
    };
    R_Date("AA19686/WG2412", 2690, 60)
    {
     Outlier("Charcoal", 1);
    };
    R_Date("AA19682/WG2408", 2605, 45)
    {
```

```
 Outlier("Charcoal", 1);
};
R_Date("AA19683/WG2409", 2605, 50)
{
 Outlier("General", 0.05);
};
R_Date("AA20282/WG2414", 2495, 65)
{
 Outlier("General", 0.05);
};
R_Date("M-1719", 2460, 140)
{
 Outlier("Charcoal", 1);
};
R_Date("AA19681/WG2407", 2380, 50)
{
 Outlier("Charcoal", 1);
};
R_Date("M-1717", 2190, 140)
{
 Outlier("General", 0.05);
};
R_Date("M-1718", 2080, 140)
{
 Outlier("Charcoal", 1);
};
R_Date("M-1716", 2050, 130)
{
 Outlier("Charcoal", 1);
};
R_Date("M-1715", 1949, 130)
{
 Outlier("Charcoal", 1);
};
R_Simulate("Sim_RO1", -794, 35);
R_Simulate("Sim_RO2", -495, 35);
R_Simulate("Sim_RO3", -1069, 35);
R_Simulate("Sim_RO4", -1027, 35);
R_Simulate("Sim_RO5", -1061, 35);
R_Simulate("Sim_RO6", -935, 35);
R_Simulate("Sim_RO7", -582, 35);
R_Simulate("Sim_RO8", -485, 35);
R_Simulate("Sim_RO9", -260, 35);
R_Simulate("Sim_RO10", -277, 35);
R_Simulate("Sim_RO11", -162, 35);
R_Simulate("Sim_RO12", -598, 35);
```

```
R_Simulate("Sim_RO13", -203, 35);
R_Simulate("Sim_RO14", -652, 35);
R_Simulate("Sim_RO15", -307, 35);
R_Simulate("Sim_RO16", -665, 35);
R_Simulate("Sim_RO17", -641, 35);
R_Simulate("Sim_RO18", -1052, 35);
R_Simulate("Sim_RO19", -110, 35);
R_Simulate("Sim_RO20", -847, 35);
R_Simulate("Sim_RO21", -1142, 35);
R_Simulate("Sim_RO22", -1122, 35);
R_Simulate("Sim_RO23", -971, 35);
R_Simulate("Sim_RO24", -880, 35);
R_Simulate("Sim_RO25", -723, 35);
R_Simulate("Sim_RO26", -531, 35);
R_Simulate("Sim_RO27", -533, 35);
R_Simulate("Sim_RO28", -973, 35);
R_Simulate("Sim_RO29", -841, 35);
R_Simulate("Sim_RO30", -704, 35);
R_Simulate("Sim_RO31", -475, 35);
R_Simulate("Sim_RO32", -867, 35);
R_Simulate("Sim_RO33", -508, 35);
R_Simulate("Sim_RO34", -727, 35);
R_Simulate("Sim_RO35", -203, 35);
R_Simulate("Sim_RO36", -208, 35);
R_Simulate("Sim_RO37", -602, 35);
R_Simulate("Sim_RO38", -211, 35);
R_Simulate("Sim_RO39", -549, 35);
R_Simulate("Sim_RO40", -355, 35);
R_Simulate("Sim_RO41", -1021, 35);
R_Simulate("Sim_RO42", -1140, 35);
R_Simulate("Sim_RO43", -125, 35);
R_Simulate("Sim_RO44", -555, 35);
R_Simulate("Sim_RO45", -961, 35);
R_Simulate("Sim_RO46", -896, 35);
R_Simulate("Sim_RO47", -310, 35);
R_Simulate("Sim_RO48", -883, 35);
R_Simulate("Sim_RO49", -835, 35);
R_Simulate("Sim_RO50", -545, 35);
R_Simulate("Sim_RO51", -765, 35);
R_Simulate("Sim_RO52", -1037, 35);
R_Simulate("Sim_RO53", -619, 35);
R_Simulate("Sim_RO54", -892, 35);
R_Simulate("Sim_RO55", -822, 35);
R_Simulate("Sim_RO56", -663, 35);
R_Simulate("Sim_RO57", -121, 35);
R_Simulate("Sim_RO58", -417, 35);
```

```
  R_Simulate("Sim_RO59", -689, 35);
  R_Simulate("Sim_RO60", -745, 35);
  R_Simulate("Sim_RO61", -207, 35);
  R_Simulate("Sim_RO62", -390, 35);
  R_Simulate("Sim_RO63", -367, 35);
  R_Simulate("Sim_RO64", -400, 35);
  R_Simulate("Sim_RO65", -951, 35);
  R_Simulate("Sim_RO66", -172, 35);
  R_Simulate("Sim_RO67", -634, 35);
  R_Simulate("Sim_RO68", -543, 35);
  R_Simulate("Sim_RO69", -345, 35);
  R_Simulate("Sim_RO70", -745, 35);
  R_Simulate("Sim_RO71", -878, 35);
  R_Simulate("Sim_RO72", -224, 35);
  R_Simulate("Sim_RO73", -388, 35);
  R_Simulate("Sim_RO74", -1130, 35);
  R_Simulate("Sim_RO75", -271, 35);
  R_Simulate("Sim_RO76", -340, 35);
  R_Simulate("Sim_RO77", -537, 35);
  R_Simulate("Sim_RO78", -667, 35);
  R_Simulate("Sim_RO79", -371, 35);
  R_Simulate("Sim_RO80", -193, 35);
  R_Simulate("Sim_RO81", -1146, 35);
  R_Simulate("Sim_RO82", -787, 35);
  R_Simulate("Sim_RO83", -954, 35);
  R_Simulate("Sim_RO84", -261, 35);
  R_Simulate("Sim_RO85", -288, 35);
  R_Simulate("Sim_RO86", -673, 35);
  R_Simulate("Sim_RO87", -946, 35);
  R_Simulate("Sim_RO88", -1116, 35);
  R_Simulate("Sim_RO89", -294, 35);
  R_Simulate("Sim_RO90", -226, 35);
  Interval ("Red Ochre");
  Interval("Red Ochre");
 };
 Boundary("End Red Ochre")
 {
  Start("Start of End Red Ochre");
  Transition("Period of End Red Ochre");
  End("End of End Red Ochre");
 };
};
Sequence("Old Copper")
{
 Boundary("Start Old Copper")
 {
```

```
 Start("Start of Start Old Copper");
 Transition("Period of Start Old Copper");
 End("End of Start Old Copper");
};
Phase("Old Copper")
{
 Sequence()
 {
  Boundary("Start Oconto");
  Phase("Oconto")
  {
   R_Date("AA19678/WG2404", 6020, 60)
   {
    Outlier("General", 0.05);
   };
   R_Date("AA20281/WG2413", 5250, 110)
   {
    Outlier("Charcoal", 1);
   };
  };
  Boundary("End Oconto");
 };
 Sequence()
 {
  Boundary("Start Allumette Island");
  Phase("Allumette Island")
  {
   R_Date("Beta-141985", 5440, 80)
   {
    Outlier("General", 0.05);
   };
   R_Date("S-509", 5240, 80)
   {
    Outlier("General", 0.05);
   };
   R_Date("Beta-141986", 5270, 40)
   {
    Outlier("General", 0.05);
   };
   R_Date("Beta-141987", 4680, 40)
   {
    Outlier("General", 0.05);
   };
  };
  Boundary("End Allumette Island");
 };
```

```
Sequence()
{
 Boundary("Start Morrison Island");
 Phase("Morrison Island")
 {
  R_Date("Beta-88851", 4860, 50)
  {
   Outlier("General", 0.05);
  };
  R_Date("Beta-215300", 4820, 40)
  {
   Outlier("General", 0.05);
  };
  R_Date("Beta-215302", 4730, 40)
  {
   Outlier("General", 0.05);
  };
  R_Date("GSC-162", 4700, 150)
  {
   Outlier("Charcoal", 1);
  };
  R_Date("Beta-88852", 4630, 40)
  {
   Outlier("General", 0.05);
  };
  R_Date("Beta-88725", 4620, 40)
  {
   Outlier("General", 0.05);
  };
  R_Date("Beta-215301", 4210, 40)
  {
   Outlier("General", 0.05);
  };
 };
 Boundary("End Morrison Island");
};
R_Date("S-1263", 5000, 80)
{
 Outlier("General", 0.05);
};
R_Date("WIS-1706", 4080, 70)
{
 Outlier("General", 0.05);
};
R_Date("Beta-247459", 4490, 40)
{
```

```
 Outlier("Charcoal", 1);
};
R_Simulate("Sim_OC1", -4424, 35);
R_Simulate("Sim_OC2", -4077, 35);
R_Simulate("Sim_OC3", -2527, 35);
R_Simulate("Sim_OC4", -4317, 35);
R_Simulate("Sim_OC5", -2651, 35);
R_Simulate("Sim_OC6", -5108, 35);
R_Simulate("Sim_OC7", -4070, 35);
R_Simulate("Sim_OC8", -4202, 35);
R_Simulate("Sim_OC9", -3570, 35);
R_Simulate("Sim_OC10", -2911, 35);
R_Simulate("Sim_OC11", -3378, 35);
R_Simulate("Sim_OC12", -3492, 35);
R_Simulate("Sim_OC13", -2687, 35);
R_Simulate("Sim_OC14", -3150, 35);
R_Simulate("Sim_OC15", -4375, 35);
R_Simulate("Sim_OC16", -4585, 35);
R_Simulate("Sim_OC17", -3717, 35);
R_Simulate("Sim_OC18", -4744, 35);
R_Simulate("Sim_OC19", -3564, 35);
R_Simulate("Sim_OC20", -4971, 35);
R_Simulate("Sim_OC21", -2743, 35);
R_Simulate("Sim_OC22", -3407, 35);
R_Simulate("Sim_OC23", -3473, 35);
R_Simulate("Sim_OC24", -4529, 35);
R_Simulate("Sim_OC25", -4516, 35);
R_Simulate("Sim_OC26", -5128, 35);
R_Simulate("Sim_OC27", -4389, 35);
R_Simulate("Sim_OC28", -3530, 35);
R_Simulate("Sim_OC29", -3771, 35);
R_Simulate("Sim_OC30", -3562, 35);
R_Simulate("Sim_OC31", -3073, 35);
R_Simulate("Sim_OC32", -2836, 35);
R_Simulate("Sim_OC33", -4059, 35);
R_Simulate("Sim_OC34", -4152, 35);
R_Simulate("Sim_OC35", -4951, 35);
R_Simulate("Sim_OC36", -2637, 35);
R_Simulate("Sim_OC37", -4632, 35);
R_Simulate("Sim_OC38", -4823, 35);
R_Simulate("Sim_OC39", -4383, 35);
R_Simulate("Sim_OC40", -3621, 35);
R_Simulate("Sim_OC41", -3206, 35);
R_Simulate("Sim_OC42", -3968, 35);
R_Simulate("Sim_OC43", -2606, 35);
R_Simulate("Sim_OC44", -3398, 35);
```

```
R_Simulate("Sim_OC45", -3846, 35);
R_Simulate("Sim_OC46", -2554, 35);
R_Simulate("Sim_OC47", -4228, 35);
R_Simulate("Sim_OC48", -2717, 35);
R_Simulate("Sim_OC49", -4823, 35);
R_Simulate("Sim_OC50", -2965, 35);
R_Simulate("Sim_OC51", -5027, 35);
R_Simulate("Sim_OC52", -5060, 35);
R_Simulate("Sim_OC53", -3018, 35);
R_Simulate("Sim_OC54", -5006, 35);
R_Simulate("Sim_OC55", -4651, 35);
R_Simulate("Sim_OC56", -3211, 35);
R_Simulate("Sim_OC57", -3842, 35);
R_Simulate("Sim_OC58", -5075, 35);
R_Simulate("Sim_OC59", -3178, 35);
R_Simulate("Sim_OC60", -4121, 35);
R_Simulate("Sim_OC61", -3974, 35);
R_Simulate("Sim_OC62", -3136, 35);
R_Simulate("Sim_OC63", -4064, 35);
R_Simulate("Sim_OC64", -3111, 35);
R_Simulate("Sim_OC65", -3122, 35);
R_Simulate("Sim_OC66", -2806, 35);
R_Simulate("Sim_OC67", -2822, 35);
R_Simulate("Sim_OC68", -3874, 35);
R_Simulate("Sim_OC69", -3926, 35);
R_Simulate("Sim_OC70", -4494, 35);
R_Simulate("Sim_OC71", -3347, 35);
R_Simulate("Sim_OC72", -3379, 35);
R_Simulate("Sim_OC73", -5060, 35);
R_Simulate("Sim_OC74", -4774, 35);
R_Simulate("Sim_OC75", -3679, 35);
R_Simulate("Sim_OC76", -4951, 35);
R_Simulate("Sim_OC77", -4159, 35);
R_Simulate("Sim_OC78", -2658, 35);
R_Simulate("Sim_OC79", -4016, 35);
R_Simulate("Sim_OC80", -4360, 35);
R_Simulate("Sim_OC81", -3628, 35);
R_Simulate("Sim_OC82", -4352, 35);
R_Simulate("Sim_OC83", -2670, 35);
R_Simulate("Sim_OC84", -3269, 35);
R_Simulate("Sim_OC85", -3652, 35);
R_Simulate("Sim_OC86", -3910, 35);
R_Simulate("Sim_OC87", -4765, 35);
R_Simulate("Sim_OC88", -2627, 35);
R_Simulate("Sim_OC89", -3643, 35);
R_Simulate("Sim_OC90", -4351, 35);
```

```
  Span("Old Copper");
  Interval("Old Copper");
 };
 Boundary("End Old Copper")
 {
  Start("Start of End Old Copper");
  Transition("Period of End Old Copper");
  End("End of End Old Copper");
 };
};
Sequence("Burnt Rollways")
{
 Boundary("Start Burnt Rollways")
 {
  Start("Start of Start Burnt Rollways");
  Transition("Period of Start Burnt Rollways");
  End("End of Start Burnt Rollways");
 };
 Phase("Burnt Rollways")
 {
  Sequence()
  {
   Boundary("Start Duck Lake");
   Phase("Duck Lake")
   {
    R_Date("Beta-099777", 3420, 50)
    {
     Outlier("Charcoal", 1);
    };
    R_Date("Beta-124454", 3400, 110)
    {
     Outlier("Charcoal", 1);
    };
   };
   Boundary("End Duck Lake");
  };
  R_Date("WIS-2269", 3630, 60)
  {
   Outlier("Charcoal", 1);
  };
  R_Date("WIS-2270", 3270, 80)
  {
   Outlier("Charcoal", 1);
  };
  R_Date("Beta-232440", 2280, 40)
  {
```

```
 Outlier("Charcoal", 1);
};
R_Simulate("Sim_BR1", -664, 35);
R_Simulate("Sim_BR2", -2253, 35);
R_Simulate("Sim_BR3", -1253, 35);
R_Simulate("Sim_BR4", -740, 35);
R_Simulate("Sim_BR5", -606, 35);
R_Simulate("Sim_BR6", -2255, 35);
R_Simulate("Sim_BR7", -1278, 35);
R_Simulate("Sim_BR8", -1550, 35);
R_Simulate("Sim_BR9", -116, 35);
R_Simulate("Sim_BR10", -2298, 35);
R_Simulate("Sim_BR11", -2055, 35);
R_Simulate("Sim_BR12", -1472, 35);
R_Simulate("Sim_BR13", -1101, 35);
R_Simulate("Sim_BR14", -1520, 35);
R_Simulate("Sim_BR15", -181, 35);
R_Simulate("Sim_BR16", -928, 35);
R_Simulate("Sim_BR17", -1885, 35);
R_Simulate("Sim_BR18", -1160, 35);
R_Simulate("Sim_BR19", -878, 35);
R_Simulate("Sim_BR20", -663, 35);
R_Simulate("Sim_BR21", -452, 35);
R_Simulate("Sim_BR22", -291, 35);
R_Simulate("Sim_BR23", -1733, 35);
R_Simulate("Sim_BR24", -664, 35);
R_Simulate("Sim_BR25", -1253, 35);
R_Simulate("Sim_BR26", -1970, 35);
R_Simulate("Sim_BR27", -809, 35);
R_Simulate("Sim_BR28", -1109, 35);
R_Simulate("Sim_BR29", -494, 35);
R_Simulate("Sim_BR30", -2358, 35);
R_Simulate("Sim_BR31", -2278, 35);
R_Simulate("Sim_BR32", -1357, 35);
R_Simulate("Sim_BR33", -241, 35);
R_Simulate("Sim_BR34", -484, 35);
R_Simulate("Sim_BR35", -62, 35);
R_Simulate("Sim_BR36", -1249, 35);
R_Simulate("Sim_BR37", -597, 35);
R_Simulate("Sim_BR38", -1780, 35);
R_Simulate("Sim_BR39", -2489, 35);
R_Simulate("Sim_BR40", -127, 35);
R_Simulate("Sim_BR41", -292, 35);
R_Simulate("Sim_BR42", -1100, 35);
R_Simulate("Sim_BR43", -1214, 35);
R_Simulate("Sim_BR44", -1229, 35);
```

```
R_Simulate("Sim_BR45", -2196, 35);
R_Simulate("Sim_BR46", -1824, 35);
R_Simulate("Sim_BR47", -294, 35);
R_Simulate("Sim_BR48", -2214, 35);
R_Simulate("Sim_BR49", -2417, 35);
R_Simulate("Sim_BR50", -1428, 35);
R_Simulate("Sim_BR51", -2403, 35);
R_Simulate("Sim_BR52", -873, 35);
R_Simulate("Sim_BR53", -702, 35);
R_Simulate("Sim_BR54", -223, 35);
R_Simulate("Sim_BR55", -454, 35);
R_Simulate("Sim_BR56", -1990, 35);
R_Simulate("Sim_BR57", -1243, 35);
R_Simulate("Sim_BR58", -2460, 35);
R_Simulate("Sim_BR59", -1917, 35);
R_Simulate("Sim_BR60", -1018, 35);
R_Simulate("Sim_BR61", -1622, 35);
R_Simulate("Sim_BR62", -306, 35);
R_Simulate("Sim_BR63", -277, 35);
R_Simulate("Sim_BR64", -1319, 35);
R_Simulate("Sim_BR65", -140, 35);
R_Simulate("Sim_BR66", -2352, 35);
R_Simulate("Sim_BR67", -870, 35);
R_Simulate("Sim_BR68", -2415, 35);
R_Simulate("Sim_BR69", -1790, 35);
R_Simulate("Sim_BR70", -2138, 35);
R_Simulate("Sim_BR71", -484, 35);
R_Simulate("Sim_BR72", -160, 35);
R_Simulate("Sim_BR73", -1871, 35);
R_Simulate("Sim_BR74", -2284, 35);
R_Simulate("Sim_BR75", -461, 35);
R_Simulate("Sim_BR76", -2067, 35);
R_Simulate("Sim_BR77", -1849, 35);
R_Simulate("Sim_BR78", -2310, 35);
R_Simulate("Sim_BR79", -1139, 35);
R_Simulate("Sim_BR80", -2422, 35);
R_Simulate("Sim_BR81", -316, 35);
R_Simulate("Sim_BR82", -583, 35);
R_Simulate("Sim_BR83", -283, 35);
R_Simulate("Sim_BR84", -923, 35);
R_Simulate("Sim_BR85", -1817, 35);
R_Simulate("Sim_BR86", -547, 35);
R_Simulate("Sim_BR87", -462, 35);
R_Simulate("Sim_BR88", -62, 35);
R_Simulate("Sim_BR89", -2021, 35);
R_Simulate("Sim_BR90", -1113, 35);
```

```
   Span("Burnt Rollways");
   Interval("Burnt Rollways");
  };
  Boundary("End Burnt Rollways")
  {
   Start("Start of End Burnt Rollways");
   Transition("Period of End Burnt Rollways");
   End("End of End Burnt Rollways");
  };
 };
 Order()
 {
 };
};
```

Simulation Code: Plus 100 Simulated Dates for Each Tradition

```
Plot()
 {
  Outlier_Model("Charcoal",Exp(1,-10,0),U(0,3),"t");
  Outlier_Model("General",T(5),U(0,4),"t");
  Sequence("Red Ochre")
  {
   Boundary("Start Red Ochre")
   {
    Start("Start of Start Red Ochre");
    Transition("Period of Start Red Ochre");
    End("End of Start Red Ochre");
   };
   Phase("Red Ochre")
   {
    R_Date("M-658", 3040, 150)
    {
     Outlier("General", 0.05);
    };
    R_Date("AA19679/WG2405", 2960, 50)
    {
     Outlier("Charcoal", 1);
    };
    R_Date("AA19685/WG2411", 2850, 50)
    {
     Outlier("Charcoal", 1);
    };
    R_Date("AA19680/WG2406", 2790, 50)
    {
     Outlier("Charcoal", 1);
    };
    R_Date("AA19677/WG2403", 2780, 65)
    {
     Outlier("General", 0.05);
    };
    R_Date("AA19684/WG2410", 2710, 50)
    {
     Outlier("Charcoal", 1);
    };
    R_Date("AA19686/WG2412", 2690, 60)
    {
     Outlier("Charcoal", 1);
    };
    R_Date("AA19682/WG2408", 2605, 45)
    {
```

```
 Outlier("Charcoal", 1);
};
R_Date("AA19683/WG2409", 2605, 50)
{
 Outlier("General", 0.05);
};
R_Date("AA20282/WG2414", 2495, 65)
{
 Outlier("General", 0.05);
};
R_Date("M-1719", 2460, 140)
{
 Outlier("Charcoal", 1);
};
R_Date("AA19681/WG2407", 2380, 50)
{
 Outlier("Charcoal", 1);
};
R_Date("M-1717", 2190, 140)
{
 Outlier("General", 0.05);
};
R_Date("M-1718", 2080, 140)
{
 Outlier("Charcoal", 1);
};
R_Date("M-1716", 2050, 130)
{
 Outlier("Charcoal", 1);
};
R_Date("M-1715", 1949, 130)
{
 Outlier("Charcoal", 1);
};
R_Simulate("Sim_RO1", -1158, 35);
R_Simulate("Sim_RO2", -140, 35);
R_Simulate("Sim_RO3", -962, 35);
R_Simulate("Sim_RO4", -304, 35);
R_Simulate("Sim_RO5", -990, 35);
R_Simulate("Sim_RO6", -943, 35);
R_Simulate("Sim_RO7", -718, 35);
R_Simulate("Sim_RO8", -578, 35);
R_Simulate("Sim_RO9", -144, 35);
R_Simulate("Sim_RO10", -158, 35);
R_Simulate("Sim_RO11", -778, 35);
R_Simulate("Sim_RO12", -778, 35);
```

```
R_Simulate("Sim_RO13", -517, 35);
R_Simulate("Sim_RO14", -1058, 35);
R_Simulate("Sim_RO15", -1140, 35);
R_Simulate("Sim_RO16", -775, 35);
R_Simulate("Sim_RO17", -739, 35);
R_Simulate("Sim_RO18", -352, 35);
R_Simulate("Sim_RO19", -799, 35);
R_Simulate("Sim_RO20", -745, 35);
R_Simulate("Sim_RO21", -1046, 35);
R_Simulate("Sim_RO22", -484, 35);
R_Simulate("Sim_RO23", -767, 35);
R_Simulate("Sim_RO24", -312, 35);
R_Simulate("Sim_RO25", -792, 35);
R_Simulate("Sim_RO26", -1072, 35);
R_Simulate("Sim_RO27", -1118, 35);
R_Simulate("Sim_RO28", -240, 35);
R_Simulate("Sim_RO29", -505, 35);
R_Simulate("Sim_RO30", -349, 35);
R_Simulate("Sim_RO31", -515, 35);
R_Simulate("Sim_RO32", -508, 35);
R_Simulate("Sim_RO33", -1082, 35);
R_Simulate("Sim_RO34", -885, 35);
R_Simulate("Sim_RO35", -629, 35);
R_Simulate("Sim_RO36", -600, 35);
R_Simulate("Sim_RO37", -606, 35);
R_Simulate("Sim_RO38", -242, 35);
R_Simulate("Sim_RO39", -743, 35);
R_Simulate("Sim_RO40", -994, 35);
R_Simulate("Sim_RO41", -618, 35);
R_Simulate("Sim_RO42", -1085, 35);
R_Simulate("Sim_RO43", -551, 35);
R_Simulate("Sim_RO44", -448, 35);
R_Simulate("Sim_RO45", -166, 35);
R_Simulate("Sim_RO46", -1027, 35);
R_Simulate("Sim_RO47", -750, 35);
R_Simulate("Sim_RO48", -516, 35);
R_Simulate("Sim_RO49", -237, 35);
R_Simulate("Sim_RO50", -1112, 35);
R_Simulate("Sim_RO51", -1063, 35);
R_Simulate("Sim_RO52", -417, 35);
R_Simulate("Sim_RO53", -691, 35);
R_Simulate("Sim_RO54", -857, 35);
R_Simulate("Sim_RO55", -243, 35);
R_Simulate("Sim_RO56", -1113, 35);
R_Simulate("Sim_RO57", -1033, 35);
R_Simulate("Sim_RO58", -298, 35);
```

```
 R_Simulate("Sim_RO59", -1138, 35);
 R_Simulate("Sim_RO60", -498, 35);
 R_Simulate("Sim_RO61", -1056, 35);
 R_Simulate("Sim_RO62", -909, 35);
 R_Simulate("Sim_RO63", -107, 35);
 R_Simulate("Sim_RO64", -968, 35);
 R_Simulate("Sim_RO65", -397, 35);
 R_Simulate("Sim_RO66", -504, 35);
 R_Simulate("Sim_RO67", -897, 35);
 R_Simulate("Sim_RO68", -356, 35);
 R_Simulate("Sim_RO69", -224, 35);
 R_Simulate("Sim_RO70", -1105, 35);
 R_Simulate("Sim_RO71", -560, 35);
 R_Simulate("Sim_RO72", -1113, 35);
 R_Simulate("Sim_RO73", -473, 35);
 R_Simulate("Sim_RO74", -184, 35);
 R_Simulate("Sim_RO75", -954, 35);
 R_Simulate("Sim_RO76", -1155, 35);
 R_Simulate("Sim_RO77", -1086, 35);
 R_Simulate("Sim_RO78", -771, 35);
 R_Simulate("Sim_RO79", -533, 35);
 R_Simulate("Sim_RO80", -1143, 35);
 R_Simulate("Sim_RO81", -1051, 35);
 R_Simulate("Sim_RO82", -820, 35);
 R_Simulate("Sim_RO83", -795, 35);
 R_Simulate("Sim_RO84", -948, 35);
 R_Simulate("Sim_RO85", -556, 35);
 R_Simulate("Sim_RO86", -286, 35);
 R_Simulate("Sim_RO87", -971, 35);
 R_Simulate("Sim_RO88", -568, 35);
 R_Simulate("Sim_RO89", -1136, 35);
 R_Simulate("Sim_RO90", -925, 35);
 R_Simulate("Sim_RO91", -1023, 35);
 R_Simulate("Sim_RO92", -258, 35);
 R_Simulate("Sim_RO93", -1009, 35);
 R_Simulate("Sim_RO94", -559, 35);
 R_Simulate("Sim_RO95", -743, 35);
 R_Simulate("Sim_RO96", -566, 35);
 R_Simulate("Sim_RO97", -730, 35);
 R_Simulate("Sim_RO98", -971, 35);
 R_Simulate("Sim_RO99", -998, 35);
 R_Simulate("Sim_RO100", -672, 35);
 Interval ("Red Ochre");
};
Boundary("End Red Ochre")
{
```

```
  Start("Start of End Red Ochre");
  Transition("Period of End Red Ochre");
  End("End of End Red Ochre");
 };
};
Sequence("Old Copper")
{
 Boundary("Start Old Copper")
 {
  Start("Start of Start Old Copper");
  Transition("Period of Start Old Copper");
  End("End of Start Old Copper");
 };
 Phase("Old Copper")
 {
  Sequence()
  {
   Boundary("Start Oconto");
   Phase("Oconto")
   {
    R_Date("AA19678/WG2404", 6020, 60)
    {
     Outlier("General", 0.05);
    };
    R_Date("AA20281/WG2413", 5250, 110)
    {
     Outlier("Charcoal", 1);
    };
   };
   Boundary("End Oconto");
  };
  Sequence()
  {
   Boundary("Start Allumette Island");
   Phase("Allumette Island")
   {
    R_Date("Beta-141985", 5440, 80)
    {
     Outlier("General", 0.05);
    };
    R_Date("S-509", 5240, 80)
    {
     Outlier("General", 0.05);
    };
    R_Date("Beta-141986", 5270, 40)
    {
```

```
   Outlier("General", 0.05);
  };
  R_Date("Beta-141987", 4680, 40)
  {
   Outlier("General", 0.05);
  };
 };
 Boundary("End Allumette Island");
};
Sequence()
{
 Boundary("Start Morrison Island");
 Phase("Morrison Island")
 {
  R_Date("Beta-88851", 4860, 50)
  {
   Outlier("General", 0.05);
  };
  R_Date("Beta-215300", 4820, 40)
  {
   Outlier("General", 0.05);
  };
  R_Date("Beta-215302", 4730, 40)
  {
   Outlier("General", 0.05);
  };
  R_Date("GSC-162", 4700, 150)
  {
   Outlier("Charcoal", 1);
  };
  R_Date("Beta-88852", 4630, 40)
  {
   Outlier("General", 0.05);
  };
  R_Date("Beta-88725", 4620, 40)
  {
   Outlier("General", 0.05);
  };
  R_Date("Beta-215301", 4210, 40)
  {
   Outlier("General", 0.05);
  };
 };
 Boundary("End Morrison Island");
};
R_Date("S-1263", 5000, 80)
```

```
{
 Outlier("General", 0.05);
};
R_Date("WIS-1706", 4080, 70)
{
 Outlier("General", 0.05);
};
R_Date("Beta-247459", 4490, 40)
{
 Outlier("Charcoal", 1);
};
R_Simulate("Sim_OC1", -5100, 35);
R_Simulate("Sim_OC2", -4334, 35);
R_Simulate("Sim_OC3", -3999, 35);
R_Simulate("Sim_OC4", -2675, 35);
R_Simulate("Sim_OC5", -3328, 35);
R_Simulate("Sim_OC6", -3499, 35);
R_Simulate("Sim_OC7", -4530, 35);
R_Simulate("Sim_OC8", -4009, 35);
R_Simulate("Sim_OC9", -4142, 35);
R_Simulate("Sim_OC10", -4462, 35);
R_Simulate("Sim_OC11", -2794, 35);
R_Simulate("Sim_OC12", -3116, 35);
R_Simulate("Sim_OC13", -2823, 35);
R_Simulate("Sim_OC14", -2511, 35);
R_Simulate("Sim_OC15", -3093, 35);
R_Simulate("Sim_OC16", -4204, 35);
R_Simulate("Sim_OC17", -3393, 35);
R_Simulate("Sim_OC18", -4733, 35);
R_Simulate("Sim_OC19", -3976, 35);
R_Simulate("Sim_OC20", -5011, 35);
R_Simulate("Sim_OC21", -2769, 35);
R_Simulate("Sim_OC22", -3866, 35);
R_Simulate("Sim_OC23", -2823, 35);
R_Simulate("Sim_OC24", -4215, 35);
R_Simulate("Sim_OC25", -4892, 35);
R_Simulate("Sim_OC26", -2720, 35);
R_Simulate("Sim_OC27", -4914, 35);
R_Simulate("Sim_OC28", -2683, 35);
R_Simulate("Sim_OC29", -4710, 35);
R_Simulate("Sim_OC30", -4418, 35);
R_Simulate("Sim_OC31", -4498, 35);
R_Simulate("Sim_OC32", -3422, 35);
R_Simulate("Sim_OC33", -4967, 35);
R_Simulate("Sim_OC34", -4333, 35);
R_Simulate("Sim_OC35", -3315, 35);
```

```
R_Simulate("Sim_OC36", -3442, 35);
R_Simulate("Sim_OC37", -2575, 35);
R_Simulate("Sim_OC38", -4178, 35);
R_Simulate("Sim_OC39", -3861, 35);
R_Simulate("Sim_OC40", -4991, 35);
R_Simulate("Sim_OC41", -2950, 35);
R_Simulate("Sim_OC42", -4743, 35);
R_Simulate("Sim_OC43", -3897, 35);
R_Simulate("Sim_OC44", -4716, 35);
R_Simulate("Sim_OC45", -3172, 35);
R_Simulate("Sim_OC46", -4652, 35);
R_Simulate("Sim_OC47", -3072, 35);
R_Simulate("Sim_OC48", -4988, 35);
R_Simulate("Sim_OC49", -2734, 35);
R_Simulate("Sim_OC50", -3085, 35);
R_Simulate("Sim_OC51", -4752, 35);
R_Simulate("Sim_OC52", -3589, 35);
R_Simulate("Sim_OC53", -4166, 35);
R_Simulate("Sim_OC54", -3008, 35);
R_Simulate("Sim_OC55", -2999, 35);
R_Simulate("Sim_OC56", -2553, 35);
R_Simulate("Sim_OC57", -5080, 35);
R_Simulate("Sim_OC58", -4448, 35);
R_Simulate("Sim_OC59", -4979, 35);
R_Simulate("Sim_OC60", -4398, 35);
R_Simulate("Sim_OC61", -4368, 35);
R_Simulate("Sim_OC62", -2939, 35);
R_Simulate("Sim_OC63", -4344, 35);
R_Simulate("Sim_OC64", -3721, 35);
R_Simulate("Sim_OC65", -4179, 35);
R_Simulate("Sim_OC66", -2998, 35);
R_Simulate("Sim_OC67", -4822, 35);
R_Simulate("Sim_OC68", -2789, 35);
R_Simulate("Sim_OC69", -2541, 35);
R_Simulate("Sim_OC70", -3025, 35);
R_Simulate("Sim_OC71", -4209, 35);
R_Simulate("Sim_OC72", -2539, 35);
R_Simulate("Sim_OC73", -3147, 35);
R_Simulate("Sim_OC74", -5120, 35);
R_Simulate("Sim_OC75", -3372, 35);
R_Simulate("Sim_OC76", -3099, 35);
R_Simulate("Sim_OC77", -2657, 35);
R_Simulate("Sim_OC78", -5086, 35);
R_Simulate("Sim_OC79", -3501, 35);
R_Simulate("Sim_OC80", -4754, 35);
R_Simulate("Sim_OC81", -3571, 35);
```

```
  R_Simulate("Sim_OC82", -3042, 35);
  R_Simulate("Sim_OC83", -4178, 35);
  R_Simulate("Sim_OC84", -2695, 35);
  R_Simulate("Sim_OC85", -2905, 35);
  R_Simulate("Sim_OC86", -4006, 35);
  R_Simulate("Sim_OC87", -3823, 35);
  R_Simulate("Sim_OC88", -3189, 35);
  R_Simulate("Sim_OC89", -3860, 35);
  R_Simulate("Sim_OC90", -4614, 35);
  R_Simulate("Sim_OC91", -3231, 35);
  R_Simulate("Sim_OC92", -3163, 35);
  R_Simulate("Sim_OC93", -4132, 35);
  R_Simulate("Sim_OC94", -4860, 35);
  R_Simulate("Sim_OC95", -3580, 35);
  R_Simulate("Sim_OC96", -4619, 35);
  R_Simulate("Sim_OC97", -4833, 35);
  R_Simulate("Sim_OC98", -3262, 35);
  R_Simulate("Sim_OC99", -4510, 35);
  R_Simulate("Sim_OC100", -5072, 35);
  Interval ("Old Copper");
 };
 Boundary("End Old Copper")
 {
  Start("Start of End Old Copper");
  Transition("Period of End Old Copper");
  End("End of End Old Copper");
 };
};
Sequence("Burnt Rollways")
{
 Boundary("Start Burnt Rollways")
 {
  Start("Start of Start Burnt Rollways");
  Transition("Period of Start Burnt Rollways");
  End("End of Start Burnt Rollways");
 };
 Phase("Burnt Rollways")
 {
  Sequence()
  {
   Boundary("Start Duck Lake");
   Phase("Duck Lake")
   {
    R_Date("Beta-099777", 3420, 50)
    {
     Outlier("Charcoal", 1);
```

```
  };
  R_Date("Beta-124454", 3400, 110)
  {
   Outlier("Charcoal", 1);
  };
 };
 Boundary("End Duck Lake");
};
R_Date("WIS-2269", 3630, 60)
{
 Outlier("Charcoal", 1);
};
R_Date("WIS-2270", 3270, 80)
{
 Outlier("Charcoal", 1);
};
R_Date("Beta-232440", 2280, 40)
{
 Outlier("Charcoal", 1);
};
R_Simulate("Sim_BR1", -1942, 35);
R_Simulate("Sim_BR2", -865, 35);
R_Simulate("Sim_BR3", -1662, 35);
R_Simulate("Sim_BR4", -1905, 35);
R_Simulate("Sim_BR5", -2113, 35);
R_Simulate("Sim_BR6", -2427, 35);
R_Simulate("Sim_BR7", -2093, 35);
R_Simulate("Sim_BR8", -2035, 35);
R_Simulate("Sim_BR9", -716, 35);
R_Simulate("Sim_BR10", -1865, 35);
R_Simulate("Sim_BR11", -1042, 35);
R_Simulate("Sim_BR12", -426, 35);
R_Simulate("Sim_BR13", -797, 35);
R_Simulate("Sim_BR14", -982, 35);
R_Simulate("Sim_BR15", -371, 35);
R_Simulate("Sim_BR16", -2250, 35);
R_Simulate("Sim_BR17", -195, 35);
R_Simulate("Sim_BR18", -312, 35);
R_Simulate("Sim_BR19", -1516, 35);
R_Simulate("Sim_BR20", -1437, 35);
R_Simulate("Sim_BR21", -1914, 35);
R_Simulate("Sim_BR22", -227, 35);
R_Simulate("Sim_BR23", -364, 35);
R_Simulate("Sim_BR24", -2315, 35);
R_Simulate("Sim_BR25", -744, 35);
R_Simulate("Sim_BR26", -2431, 35);
```

```
R_Simulate("Sim_BR27", -1211, 35);
R_Simulate("Sim_BR28", -790, 35);
R_Simulate("Sim_BR29", -1182, 35);
R_Simulate("Sim_BR30", -301, 35);
R_Simulate("Sim_BR31", -2191, 35);
R_Simulate("Sim_BR32", -1459, 35);
R_Simulate("Sim_BR33", -1876, 35);
R_Simulate("Sim_BR34", -398, 35);
R_Simulate("Sim_BR35", -1187, 35);
R_Simulate("Sim_BR36", -1091, 35);
R_Simulate("Sim_BR37", -1856, 35);
R_Simulate("Sim_BR38", -629, 35);
R_Simulate("Sim_BR39", -661, 35);
R_Simulate("Sim_BR40", -969, 35);
R_Simulate("Sim_BR41", -2003, 35);
R_Simulate("Sim_BR42", -1830, 35);
R_Simulate("Sim_BR43", -2281, 35);
R_Simulate("Sim_BR44", -881, 35);
R_Simulate("Sim_BR45", -1525, 35);
R_Simulate("Sim_BR46", -1825, 35);
R_Simulate("Sim_BR47", -1785, 35);
R_Simulate("Sim_BR48", -995, 35);
R_Simulate("Sim_BR49", -1603, 35);
R_Simulate("Sim_BR50", -652, 35);
R_Simulate("Sim_BR51", -1571, 35);
R_Simulate("Sim_BR52", -748, 35);
R_Simulate("Sim_BR53", -1827, 35);
R_Simulate("Sim_BR54", -928, 35);
R_Simulate("Sim_BR55", -493, 35);
R_Simulate("Sim_BR56", -2014, 35);
R_Simulate("Sim_BR57", -1646, 35);
R_Simulate("Sim_BR58", -1129, 35);
R_Simulate("Sim_BR59", -1901, 35);
R_Simulate("Sim_BR60", -1829, 35);
R_Simulate("Sim_BR61", -139, 35);
R_Simulate("Sim_BR62", -2305, 35);
R_Simulate("Sim_BR63", -2001, 35);
R_Simulate("Sim_BR64", -1075, 35);
R_Simulate("Sim_BR65", -279, 35);
R_Simulate("Sim_BR66", -2074, 35);
R_Simulate("Sim_BR67", -445, 35);
R_Simulate("Sim_BR68", -2308, 35);
R_Simulate("Sim_BR69", -321, 35);
R_Simulate("Sim_BR70", -2053, 35);
R_Simulate("Sim_BR71", -2230, 35);
R_Simulate("Sim_BR72", -472, 35);
```

```
  R_Simulate("Sim_BR73", -802, 35);
  R_Simulate("Sim_BR74", -1749, 35);
  R_Simulate("Sim_BR75", -1462, 35);
  R_Simulate("Sim_BR76", -1309, 35);
  R_Simulate("Sim_BR77", -1383, 35);
  R_Simulate("Sim_BR78", -1635, 35);
  R_Simulate("Sim_BR79", -507, 35);
  R_Simulate("Sim_BR80", -343, 35);
  R_Simulate("Sim_BR81", -1818, 35);
  R_Simulate("Sim_BR82", -2266, 35);
  R_Simulate("Sim_BR83", -2146, 35);
  R_Simulate("Sim_BR84", -695, 35);
  R_Simulate("Sim_BR85", -403, 35);
  R_Simulate("Sim_BR86", -446, 35);
  R_Simulate("Sim_BR87", -1206, 35);
  R_Simulate("Sim_BR88", -594, 35);
  R_Simulate("Sim_BR89", -335, 35);
  R_Simulate("Sim_BR90", -89, 35);
  R_Simulate("Sim_BR91", -1028, 35);
  R_Simulate("Sim_BR92", -1851, 35);
  R_Simulate("Sim_BR93", -1681, 35);
  R_Simulate("Sim_BR94", -162, 35);
  R_Simulate("Sim_BR95", -2109, 35);
  R_Simulate("Sim_BR96", -1544, 35);
  R_Simulate("Sim_BR97", -709, 35);
  R_Simulate("Sim_BR98", -587, 35);
  R_Simulate("Sim_BR99", -1805, 35);
  R_Simulate("Sim_BR100", -2336, 35);
  Interval ("Burnt Rollways");
 };
 Boundary("End Burnt Rollways")
 {
  Start("Start of End Burnt Rollways");
  Transition("Period of End Burnt Rollways");
  End("End of End Burnt Rollways");
 };
};
Order()
{
};
};
```

KDE Plot

The purpose of these summation efforts are simply to produce a general chronological framework for the copper-associated dates upon which we could organize and easily visualize temporal relationships between major cultural and natural changes across the region and time periods in question. Alongside other independent archaeological data sets, the dates serve as a very rough proxy for population and settlement fluctuations over time. The method used here to visualize fluctuations in the regional radiocarbon record, or the underlying distribution of dates across the archaeological record of the early copper cultures of the Great Lakes region, Kernel Density Estimation Modeling (e.g., Bronk Ramsey 2017; Brunner et al. 2020; Feeser et al. 2019; Hennius 2020), differs from traditional Summed Probability Distribution approaches in that it combines both frequentist and Bayesian approaches for modeling dates, but takes into account the density of dates at particular points in time. We recognize the complexities with any direct correlation between the underlying distribution of the regional radiocarbon record and fluctuations in population estimates (e.g., Armit et al. 2013; Bamforth and Grund 2012; Contreras and Meadows 2014; Timpson et al. 2015; Torfing 2015; Williams 2012). We simply offer that in a general manner the radiocarbon record may fluctuate in meaningful patterns that are useful for exploring and visualizing long term cultural histories.

Primary KDE Plot

Ages with greater than 150-year error ranges excluded. See alternative KDE plot for ages with error ranges of 150 years or more included.

```
KDE_Plot()
 {
  Sequence("All Copper")
  {
   Boundary("All Copper")
   {
    Start("Start of Start All Copper");
    Transition("Period of Start All Copper");
    End("End of Start All Copper");
   };
   Phase("All Copper")
   {
    Phase("Red Ochre")
    {
     R_Date("M-658", 3040, 150);
     R_Date("AA19679/WG2405", 2960, 50);
     R_Date("AA19685/WG2411", 2850, 50);
     R_Date("AA19680/WG2406", 2790, 50);
     R_Date("AA19677/WG2403", 2780, 65);
     R_Date("AA19684/WG2410", 2710, 50);
     R_Date("AA19686/WG2412", 2690, 60);
     R_Date("AA19682/WG2408", 2605, 45);
     R_Date("AA19683/WG2409", 2605, 50);
     R_Date("AA20282/WG2414", 2495, 65);
     R_Date("M-1719", 2460, 140);
     R_Date("AA19681/WG2407", 2380, 50);
     R_Date("M-1717", 2190, 140);
     R_Date("M-1718", 2080, 140);
     R_Date("M-1716", 2050, 130);
     R_Date("M-1715", 1949, 130);
    };
    Phase("Old Copper")
    {
     Phase("Oconto")
     {
      R_Date("AA19678/WG2404", 6020, 60);
      R_Date("AA20281/WG2413", 5250, 110);
     };
     Phase("Allumette Island")
     {
      R_Date("Beta-141985", 5440, 80);
      R_Date("S-509", 5240, 80);
      R_Date("Beta-141986", 5270, 40);
      R_Date("Beta-141987", 4680, 40);
```

```
 };
 Phase("Morrison Island")
 {
  R_Date("Beta-88851", 4860, 50);
  R_Date("Beta-215300", 4820, 40);
  R_Date("Beta-215302", 4730, 40);
  R_Date("GSC-162", 4700, 150);
  R_Date("Beta-88852", 4630, 40);
  R_Date("Beta-88725", 4620, 40);
  R_Date("Beta-215301", 4210, 40);
 };
 Phase("Osceola")
 {
  R_Date("WIS-1706", 4080, 70);
 };
 Phase("Reigh")
 {
  R_Date("Beta-247459", 4490, 40);
 };
 R_Date("S-1263", 5000, 80);
};
Phase("Burnt Rollways")
{
 Phase("Duck Lake")
 {
  R_Date("Beta-099777", 3420, 50);
  R_Date("Beta-124454", 3400, 110);
 };
 R_Date("WIS-2269", 3630, 60);
 R_Date("WIS-2270", 3270, 80);
 R_Date("Beta-232440", 2280, 40);
};
R_Date("Beta-29787/88-3-49", 3300, 60);
R_Date("Beta-29789/88-3-93", 3260, 70);
R_Date("Beta-24330/88-3-51", 1570, 100);
R_Date("Beta-485561", 3350, 30);
R_Date("Beta-243582", 1540, 40);
R_Date("Beta-134256", 7690, 40);
R_Date("WG613", 7305, 60);
R_Date("Beta-370311", 3730, 30);
R_Date("CAMS-174540", 4345, 30);
R_Date("Beta-511976", 6900, 30);
R_Date("Beta-511977", 3680, 30);
R_Date("M-1275d,e,f,g", 4110, 130);
R_Date("M-1275c", 2800, 120);
R_Date("M-1384", 4420, 150);
```

```
   R_Date("M-1390", 4400, 150);
   R_Date("M-1388", 3460, 130);
   R_Date("M-1385", 3360, 130);
   R_Date("M-1389", 3310, 130);
   R_Date("M-1387", 3220, 130);
   R_Date("UCIAMS-190517", 2235, 15);
   R_Date("Beta-511973", 7310, 30);
   R_Date("TO-2213", 4590, 50);
   R_Date("TO-2215", 4630, 60);
   R_Date("TO-2441", 4420, 60);
   R_Date("Beta-343669", 5690, 30);
   R_Date("M-1386", 3370, 130);
   R_Date("TO-2216", 5940, 90);
   R_Date("Beta-492176", 5730, 30);
   R_Date("Beta-511975", 6380, 30);
   R_Date("Beta-511974", 1770, 30);
   Interval ("All Copper");
  };
  Boundary("End All Copper")
  {
   Start("Start of End All Copper");
   Transition("Period of End All Copper");
   End("End of End All Copper");
  };
 };
};
```

Alternative KDE Plot

All ages incorporated into the plot, including those with error ranges of 150 years or more.

```
KDE_Plot()
 {
  Sequence("All Copper")
  {
   Boundary("All Copper")
   {
    Start("Start of Start All Copper");
    Transition("Period of Start All Copper");
    End("End of Start All Copper");
   };
   Phase("All Copper")
   {
    Phase("Red Ochre")
    {
     R_Date("M-658", 3040, 150);
     R_Date("AA19679/WG2405", 2960, 50);
     R_Date("AA19685/WG2411", 2850, 50);
     R_Date("AA19680/WG2406", 2790, 50);
     R_Date("AA19677/WG2403", 2780, 65);
     R_Date("AA19684/WG2410", 2710, 50);
     R_Date("AA19686/WG2412", 2690, 60);
     R_Date("AA19682/WG2408", 2605, 45);
     R_Date("AA19683/WG2409", 2605, 50);
     R_Date("AA20282/WG2414", 2495, 65);
     R_Date("M-1719", 2460, 140);
     R_Date("AA19681/WG2407", 2380, 50);
     R_Date("M-1717", 2190, 140);
     R_Date("M-1718", 2080, 140);
     R_Date("M-1716", 2050, 130);
     R_Date("M-1715", 1949, 130);
    };
    Phase("Old Copper")
    {
     Phase("Oconto")
     {
      R_Date("AA19678/WG2404", 6020, 60);
      R_Date("AA20281/WG2413", 5250, 110);
      R_Date("C-836", 5600, 600);
      R_Date("C-837/C-839", 7510, 600);
      R_Date("GAK", 4540, 400);
     };
     Phase("Allumette Island")
     {
      R_Date("Beta-141985", 5440, 80);
      R_Date("S-509", 5240, 80);
      R_Date("Beta-141986", 5270, 40);
```

```
  R_Date("Beta-141987", 4680, 40);
 };
 Phase("Morrison Island")
 {
  R_Date("Beta-88851", 4860, 50);
  R_Date("Beta-215300", 4820, 40);
  R_Date("Beta-215302", 4730, 40);
  R_Date("GSC-162", 4700, 150);
  R_Date("Beta-88852", 4630, 40);
  R_Date("Beta-88725", 4620, 40);
  R_Date("Beta-215301", 4210, 40);
 };
 Phase("Osceola")
 {
  R_Date("WIS-1706", 4080, 70);
  R_Date("M-643", 3450, 250);
 };
 Phase("Reigh")
 {
  R_Date("Beta-247459", 4490, 40);
  R_Date("M-644", 3660, 250);
 };
 R_Date("S-1263", 5000, 80);
};
Phase("Burnt Rollways")
{
 Phase("Duck Lake")
 {
  R_Date("Beta-099777", 3420, 50);
  R_Date("Beta-124454", 3400, 110);
 };
 R_Date("WIS-2269", 3630, 60);
 R_Date("WIS-2270", 3270, 80);
 R_Date("Beta-232440", 2280, 40);
};
R_Date("Beta-29787/88-3-49", 3300, 60);
R_Date("Beta-29789/88-3-93", 3260, 70);
R_Date("Beta-24330/88-3-51", 1570, 100);
R_Date("Beta-485561", 3350, 30);
R_Date("Beta-243582", 1540, 40);
R_Date("Beta-134256", 7690, 40);
R_Date("WG613", 7305, 60);
R_Date("Beta-370311", 3730, 30);
R_Date("CAMS-174540", 4345, 30);
R_Date("Beta-511976", 6900, 30);
R_Date("Beta-511977", 3680, 30);
```

```
   R_Date("M-1275d,e,f,g", 4110, 130);
   R_Date("M-1275c", 2800, 120);
   R_Date("M-1384", 4420, 150);
   R_Date("M-1390", 4400, 150);
   R_Date("M-1388", 3460, 130);
   R_Date("M-1385", 3360, 130);
   R_Date("M-1389", 3310, 130);
   R_Date("M-1387", 3220, 130);
   R_Date("UCIAMS-190517", 2235, 15);
   R_Date("Beta-511973", 7310, 30);
   R_Date("TO-2213", 4590, 50);
   R_Date("TO-2215", 4630, 60);
   R_Date("TO-2441", 4420, 60);
   R_Date("Beta-343669", 5690, 30);
   R_Date("M-1386", 3370, 130);
   R_Date("TO-2216", 5940, 90);
   R_Date("Beta-492176", 5730, 30);
   R_Date("Beta-511975", 6380, 30);
   R_Date("Beta-511974", 1770, 30);
   R_Date("Beta-29788/88-3-56", 7870, 350);
   R_Date("M-320", 3000, 350);
   R_Date("M-371e", 3800, 500);
   R_Date("W-291", 3310, 200);
   Interval ("All Copper");
  };
  Boundary("End All Copper")
  {
   Start("Start of End All Copper");
   Transition("Period of End All Copper");
   End("End of End All Copper");
  };
 };
};
```

References Cited

Armit, I., G.T. Swindles, and K. Becker

2013 From dates to demography in later prehistoric Ireland? Experimental approaches to the meta-analysis of large 14C datasets. *Journal of Archaeological Science* 40(1):433-438.

Bamforth, Douglas B. and B. Grund
2012 Radiocarbon calibration curves, summed probability distributions, and early Paleoindian population trends in North America. *Journal of Archaeological Science* 39(6):1768-1774.

Bayliss, Alex
2015 Quality in Bayesian chronological models in archaeology. *World Archaeology* 47(4):677-700.

2007 Bayesian buildings: an introduction for the numerically challenged. *Vernacular Architecture* 38(1):75-86.

Bayliss, Alex, Christopher Bronk Ramsey, Johannes Van der Plicht, and Alasdair Whittle
2007 Bradshaw and Bayes: towards a timetable for the Neolithic. *Cambridge Archaeological Journal* 17(S1):1-28.

Becerra-Valdivia, Lorena and Thomas Higham
2020 The timing and effect of the earliest human arrivals in North America. *Nature* 584.7819:93-97.

Bronk Ramsey, Christopher
2021 OxCal Program, Version 4.4.

2017 Methods for Summarizing Radiocarbon Datasets. *Radiocarbon* 59:1809-1833.

2009a Bayesian analysis of radiocarbon dates. *Radiocarbon* 51(1):337-360.

2009b Dealing with outliers and offsets in radiocarbon dating. *Radiocarbon* 51(3):1023-1045.

Brunner, Mirco, Jonas von Felton, Martin Hinz, and Albert Hafner

2020 Central European Early Bronze Age Chronology Revisited: A Bayesian Examination of Late-Scale Radiocarbon Dating. *PLOS One* 10.1371/journal.pone.0243719.

Buchanan, Briggs, et al.
2021 Bayesian revision of the Folsom age range using IntCal20. *PaleoAmerica* DOI:10.1080/20555563.2021.1890401.

Buck, Caitlin E., William G. Cavanagh, and Cliff Litton
1996 *Bayesian approach to interpreting archaeological data*. Wiley, New York. Buck,

Buck, Caitlin E., Cliff Litton, and E.M. Scott
1994 Making the most of radiocarbon dating: some statistical considerations. *Antiquity* 68(259):252-263.

Buck, Caitlin E., Cliff Litton, and A.F. Smith
1992 Calibration of radiocarbon results pertaining to related archaeological events. *Journal of Archaeological Science* 19(5):497-512.

Buck, Caitlin E., J.B. Kenworthy, Cliff Litton, and A.F. Smith
1991 Combining archaeological and radiocarbon information: a Bayesian approach to calibration. *Antiquity* 65(249):808-821.

Christen, J. Andres
1994 Summarizing a set of radiocarbon determinations: a robust approach. *Applied Statistics* 43(3):489-503.

Christen, J. Andres and Cliff Litton
1995 A Bayesian approach to wiggle-matching. *Journal of Archaeological Science* 22(6):719-725.

Christen, J. Andres., R.S. Clymo, and Cliff Litton
1995 A Bayesian approach to the use of 14C dates in the estimation of the age of peat. *Radiocarbon* 37(2):431-441.

Contreras, D.A. and J. Meadows
2014 Summed probability calibrations as a population proxy: a critical evaluation using a realistic simulation approach. *Journal of Archaeological Science* 52:591-608.

Feeser, Ingo, Walter Dorfler, Jutta Kneisel
2019 Human Impact and Population Dynamics in the Neolithic and Bronze Age: Multi-proxy Evidence from North-Western Central Europe. *The Holocene* 29(10):1596-1606.

Hennius, Andreas
2020 Towards a Refined Chronology of Prehistoric Pitfall Hunting in Sweden. *European Journal of Archaeology* 23(4):530-546.

Higham, Tom, et al.
2014 The timing and spatiotemporal patterning of Neanderthal disappearance. *Nature* 512.7514:306-309.

Lee, Sharen and Christopher Bronk Ramsey
2012 Development and application of the trapezoidal model for archaeological chronologies. *Radiocarbon* 54(1):107-122.

Reimer, P. J., W. E. Austin, E. Bard, A. Bayliss, P. G. Blackwell, C. B. Ramsey, M. Butzin, et al.
2020 The IntCal20 Northern Hemisphere Radiocarbon Age Calibration Curve (0–55 cal kBP. *Radiocarbon* 62 (4): 725–757.

Scott, Eric M. and Paul J. Reimer

2009 Calibration introduction. *Radiocarbon* 51(1):283-285.

Timpson, A., K. Manning, and S. Shennan
2015 Inferential mistakes in population proxies: A response to Torfing's "Neolithic population and summed probability distribution of 14 C dates." *Journal of Archaeological Science* 63:199-202.

Torfing, T.
2015 Neolithic population and summed probability distribution of 14 C dates. *Journal of Archaeological Science* 63:193-198.

Whittle, Alasdair W.R., Frances M.A. Healy, and Alex Bayliss
2011 *Gathering time: dating the early Neolithic enclosures of southern Britain and Ireland*. Oxbow Books, London.

Williams, A.N.
2012 The use of summed radiocarbon probability distributions in archaeology: a review of methods. *Journal of Archaeological Science* 39(3):578-589.
